# Supplementary material for: Evaluation of variation in special educational needs provision and its impact on health and education using administrative records for England: umbrella protocol for a mixed-methods research programme
Source: BMJ Open. 2023 Nov 2;13(11):e072531. doi: 10.1136/bmjopen-2023-072531 (PMC10626865; doi:10.1136/bmjopen-2023-072531)
Supplement: Supplementary data [file bmjopen-2023-072531supp001.pdf]

Appendix 1: Overview of Special Educational Needs provision in England

National policies in the UK and in many high-income countries require schools to make adaptations to meet the needs of children who have health, learning or behavioural problems, that impact their ability to learn; these children are referred to collectively as having special educational needs (SEN). In England, children with SEN "*have a significantly greater difficulty in learning than the majority of others of the same age, or have a disability which prevents them from making use of facilities generally provided by mainstream schools*" (see the study glossary in Appendix 2 below).[1]

There are four broad areas of SEN that state-funded schools are required to support: communication and social interactions, cognition and learning, social, emotional and mental health and sensory or physical disabilities (see Box 1). Interventions and adjustments in schools for children with SEN, referred to as *SEN provision*, are intended to improve inclusion and participation in education and support children's health and wellbeing.[1] Approximately 7% of children in England attend private schools each year,[2] which do not have the same legal obligations regarding SEN identification and provision.

*Box 1 – Broad areas of special educational needs (SEN) that schools should plan for, with sub-categories set out by the Special Educational Needs and Disability code of practice in England[1] (at the time of publication)**

Communication and social interactions
- Speech, language and communication needs
- Autistic Spectrum Disorders

Cognition and learning:
- Moderate learning difficulties
- Severe learning difficulties
- Profound and multiple learning difficulties
- Specific learning difficulties (that is learning difficulties affecting one or more specific aspects of learning, such as dyslexia, dyscalculia or dyspraxia)

Social, emotional and mental health*
- Examples could be attention-deficit/hyperactivity disorder, behavioural difficulties, anxiety, depression, eating disorders

Sensory and/or physical disabilities:
- Vision impairment
- Hearing impairment
- Multi-sensory impairment
- Physical disability requiring additional ongoing support and/or equipment to access all the opportunities available to their peers

**Note that the sub-categories changed in 2014/15 following reforms to SEN system: "Social, emotional and mental health difficulties" were introduced in 2014/15, while "Behaviour, Emotional & Social Difficulties" were removed.*

Since 2015, approximately one in six children in England are recorded by schools as receiving any SEN provision each year,[3] and one-third of all children have a record of any SEN provision at least once during their time in education.[4,5] There are two categories of SEN provision offered in England,

Zylbersztejn A, *et al. BMJ Open* 2023; 13:e072531. doi: 10.1136/bmjopen-2023-072531

SEN support and *Education, Health and Care Plans (EHCPs)*. These categories were introduced following Government education reforms in 2014/15, replacing the older categories of *School Action/School Action Plus* (together referred to as *SEN without Statement*) and *Statements of SEN*, respectively (see study glossary in Appendix 2 for details).

The majority of pupils with any recorded SEN provision receive *SEN support*. SEN support is arranged and funded by the schools and can include short-term interventions such as speech or language therapy or extra support for reading. The first assessment for SEN support is usually carried out by the school's teachers, Special Educational Needs Coordinator (SENCo), or after class teachers, who seek to identify children making less than expected educational progress or with additional social needs relative to their peers. In 2018/19 (the last academic year before the COVID-19 pandemic), 11.9% of pupils had SEN support recorded, with the vast majority provided in mainstream schools.[6]

A smaller proportion of children receive an EHCP, which involves additional and more intensive provision arranged and partly funded by local authorities for children whose needs cannot be fully met by SEN support.[3,7] Support may range from extra help by a part-time teaching assistant to full-time care by multiple staff in a special school.[1] An assessment for an EHCP can be requested by parents, schools or health or social care professionals. The assessment is carried out by the local authority, who are required to fill in a legal document setting out the special measures to be provided to meet the child's needs across education, health and social care.[3,7] In 2018/19, 3.1% of pupils had a record of an EHCP, half of whom were enrolled in a special school (1.6% of all children).[6]

Appendix 2: HOPE study glossary

Confounding: The bias caused by shared causes of exposure and outcome.[8]

Confounder: A variable that can be used to adjust for confounding.[8]

Disability: under the Equality Act 2010 a disability is "a physical or mental impairment which has a long-term and substantial adverse effect on their ability to carry out normal day-to-day activities". Not all children with SEN have a disability, and not all disabled children have SEN, but there is significant overlap.[1,9]

Education, health and care plan, EHCP (known as SEN Statement prior to education reform in 2014/15): more intense provision arranged by local authorities, involving a legal document setting special measures provided by local authorities to meet a child's needs across education, health and social care.[1]

Health phenotypes: health conditions which can be indicated in administrative health records using diagnostic data (such as recorded diagnoses or procedures). In HOPE study we focus on health conditions associated with higher need for additional support for SEN than for their peers

Recorded SEN provision: Schools record information on children identified as needing SEN provision in school censuses returned to the Department for Education (DfE). Recorded SEN indicates identification of a child's primary need using fixed categories (see Box 1) and level of appropriate intervention (either SEN support or EHCP).[2] However, there is no centrally collated data on when and what type of intervention was received. Note that for simplicity we refer to *recorded SEN provision* as *SEN provision* in the protocol, although recorded SEN provision does not evidence that SEN provision is actually received or whether it is appropriate.[2]

Reasonable adjustments: schools have a duty to support pupils with medical conditions and make reasonable adjustments for children with disabilities, including the provision of auxiliary aids and services for disabled children. Not all children with disabilities have SEN, therefore reasonable adjustments are not considered SEN intervention.[1]

Special educational needs, SEN: a child has SEN if they have a health, learning or behavioural problems, that impact their ability to learn and require for special educational provision to be made for him or her.[1] Disability included in definition of SEN is a disability that prevents the child from using facilities generally provided for their peers in mainstream settings.[1]

Special educational needs or disability (SEND): Children with disabilities do not inevitably have SEN, but sometimes the term SEND is used to include children whose disabilities do impact their ability to access the mainstream school curriculum. The term SEND is also used by government departments in England to encompass all children with SEN, disabilities, or both.

SEN support (known as School Action and School Action Plus prior to education reform in 2014/15): SEN provisions arranged and funded by the school and provided almost entirely in mainstream schools. Prior to education reform in 2014/15, children could receive support as part of School Action or more intense support as part of School Action Plus. We consider all of these categories together for all analyses in the HOPE study.

SEN provision: provision different from or additional to that normally available to pupils of the same age[1]

Stages of the national curriculum[10]

The typical educational journey in England is segmented into a variety of blocks called "key stages", during which children are expected to learn a set of subjects. At the end of each key stage, there is an assessment of child's performance.

Early Years Foundation Stage (EYFS): covers ages 3 to 5. At the end of EYFS (final term of *reception* i.e., the year when a child turns 5 years old) children are assessed by class teacher on the basis of classroom observations. Assessed areas currently include communication, physical, personal, social and emotional development, literacy, mathematics, understanding the world, and expressive arts and designs (note that assessment has changed over time).

Key Stage 1: covers Year 1 (age 5 to 6) and Year 2 (age 6 to 7). In Year 1 there is a phonics screening check, whilst in Year 2 children take national tests in English reading and maths, and are assessed in maths, science, and English reading and writing by teachers.

Key Stage 2: covers Year 3 (age 7 to 8) to Year 6 (age 10 to 11). At the end of Key Stage 2, children take national tests in English reading, maths, and grammar, punctuation and spelling, and are assessed by teachers in English writing and science.

Key Stage 3: covers Years 7 (Age 11 to 12) to 9 (age 13 to 14). Year 7 is considered the start of "secondary" school. After 2008, the national curriculum does not require any assessments. Before, 2008 there were Standardised Assessment Tests (SATs).

Key Stage 4: covers educational Years 10 (age 14 to 15) and 11 (age 15 to 16). For most students, this includes being examined using the (International) General Certificate of Secondary Education (GCSE). Special schools provide NCFE qualifications which are highly reputable vocational and work-related courses, designed to accommodate the needs of employers for immediate full-time employment and allow students to progress to higher education to degree level.

Appendix 3 - Additional Results & Methods

Appendix Table 1 – Overview of number of children entering primary school (Year 1) captured in ECHILD

Academic year	All children in school in Year 1 (recorded in NPD)	All children in school in Year 1 linked to a HES record	All children in school in Year 1 linked to a birth admission in HES
2009/10	574,833	543,135 (94.5%)	449,166 (78.1%)
2010/11	587,163	553,705 (94.3%)	474,295 (80.8%)
2011/12	600,455	565,764 (94.2%)	489,041 (81.4%)
2012/13	620,754	584,215 (94.1%)	498,376 (80.3%)
2013/14	647,299	605,217 (93.5%)	504,161 (77.9%)
2014/15	645,292	601,087 (93.1%)	527,385 (81.7%)
2015/16	659,361	613,953 (93.1%)	556,171 (84.3%)
2016/17	669,197	623,097 (93.1%)	570,284 (85.2%)
total	5,004,354	4,690,173 (93.7%)	4,068,879 (81.3%)

HES=Hospital Episode Statistics, NPD=National Pupil Database

Search terms for literature review for health phenotype definition

("cognitive impairment*" OR "cognitive delay*" OR "learning disabilit*" OR "intellectual disabilit*" OR "*developmental disability* " OR "developmental delay*" OR "special education" OR "special educational needs" OR "special educational need" OR "additional learning need*" OR "special school*" OR "additional learning support") AND ("systematic review" OR "meta-analysis" OR "meta analysis" OR "cohort study" OR "observational study" OR "population based" OR "population-based" OR "register study" OR "registry") AND (child* or adolescen* or pupil* or teeange* or "school student*") AND (congenital or "birth defect*" or chromosomal or "chronic disease" or "chronic condition*" or "long term condition*" or "long term disease" or "life limiting" or "end stage" or palliative or "liver disease" or asthma or cancer or malignan* or diabetes or obes* or encephalpath* or "cystic fibrosis" or "renal disease" or "kidney disease" or "heart disease" or cardiomyopathy or endocarditis or "lung disease" or "liver disease" or epilepsy or epileptic* or seizure* or "sickle cell" or "physical condition" or or illness or "brain injury" or "brain trauma" or cardiovascular or stroke or preterm or "birth weight" or birthweight or neonatal or gestation* or asphyxia or "spinal injury" or hydrocephalus or "nervous system" or autoimmune or eczema or arthritis or psoriasis or infection or medication)

Zylbersztejn A, *et al. BMJ Open* 2023; 13:e072531. doi: 10.1136/bmjopen-2023-072531

Appendix Table 2 – Roadmap for causal investigations in HOPE, with an exemplar of cleft lip and palate

	**Steps in the design of the study:**	**Exemplar: *Children with cleft lip and palate (CLP) abnormalities***
**1.**	Articulate the scientific question and specify the background knowledge:	*Does special educational needs (SEN) provision improve the health and educational outcomes of children with CLP?*
	a) define the population of interest	Children with CLP identified in HES before age 5, born between 2003 and 2012, who started compulsory education between 2008 and 2018, with linked HES and NPD data
	b) specify the outcomes	• Number of days in contact with an accident and emergency department by Year 6<br>• Number of unplanned school absences by Year 6
	c) specify the intervention ("exposure")	• SEN support vs no recorded SEN provision<br>• SEN support vs EHCP
	d) draw assumptions regarding exposure, outcome and their common causes	*Draw DAG that includes unmeasured variables (as relevant) and identifies a minimum set of confounding variables*
	e) translate the causal question in terms of a contrast of means of potential outcomes*	*What are the benefits for the children who did receive SEN provision?*<br>→ Average treatment effect in the treated (ATT)<br><br>*Would other children with CLP benefit from SEN?*<br>→ Average treatment effect in the non-treated (ATNT)<br><br>*What would be the consequence of a new policy that increases the provision of SEN for all children with CLP? Or for those with more severe CLP?*<br>→ Interventional treatment effect (ITE)
**2.**	Can the question be addressed with the data at hand?	
	a) is the exposure well-defined and available in the data?	We have access to two categories of recorded SEN provision (SEN support and EHCP) and treat each of them to represent delivered support (covering a variety of different interventions)
	b) is the exposure suitable/available for everyone in the population of interest?	Some groups of children appear not to be eligible for an EHCP
	c) is there an issue of selection bias?	Missing values affect some of the confounding variables
**3.**	Causal contrasts	

Zylbersztejn A, *et al. BMJ Open* 2023; 13:e072531. doi: 10.1136/bmjopen-2023-072531

	a) For which (sub-)population we wish to address the causal question?	Depending on the question:<br>• Children with CLP who have a record of any SEN provision;<br>• Children with CLP who do not have a record of any SEN provision;<br>• All children with CLP;<br>• Children with more severe CLP;
	b) On which scale?	For both outcomes we will examine:<br>– rate ratios<br>– rate differences
**4.**	Estimation:	
	a) Which estimation approach would target the causal contrast we are interested in?	For the ATT and ATNT:<br>• G-computation;<br>• Inverse probability weighting (IPW) of marginal structural models (MSMs), with alternative approaches to specify the propensity score;<br>• Doubly robust methods;<br>• Difference-in-Differences;<br><br>For the ITE:<br>• G-computation<br>• IPW of MSMs, with alternative approaches to specify the propensity score<br>• Doubly robust methods
	b) Are the assumptions invoked by alternative estimation approaches defensible?	• Unmeasured confounding may be at play;<br>• Parametric models used may be misspecified but robust methods can be used;
**5.**	Interpretation	
	a) Are the results comparable? And why not if not?	If comparable, results are more robust to misspecification and unmeasured confounding
	b) Triangulating results and compare with external evidence	Current evidence on impact is limited

**Potential outcomes are the outcomes that would occur under intervention on the exposure*
ATNT= Average treatment effect in the non-treated, ATT=Average treatment effect in the treated, CLP= cleft lip and palate, DAG=Directed Acyclic Graph, EHCP=Education, Health and Care Plan, HES=Hospital Episode Statistics, IPW=Inverse Probability Weighting, ITE=Interventional treatment effect, MSM=Marginal Structural Models, NPD=National Pupil Database, SEN=Special Educational Needs

Zylbersztejn A, *et al. BMJ Open* 2023; 13:e072531. doi: 10.1136/bmjopen-2023-072531

References

1. Department for Education. Special educational needs and disability code of practice: 0 to 25 years. Published online 2015. doi:DFE-00205-2013

2. Jay MA, Grath-Lone LM, Gilbert R. Data Resource: the National Pupil Database (NPD). *International Journal of Population Data Science*. 2019;4(1). doi:10.23889/ijpds.v4i1.1101

3. The National Audit Office. Support for pupils with special educational needs and disabilities in England. Published online 2019. Accessed January 4, 2023. https://www.nao.org.uk/wp-content/uploads/2019/09/Support-for-pupils-with-special-education-needs.pdf

4. Jay MA, Gilbert R. Special educational needs, social care and health. *Archives of Disease in Childhood*. 2021;106(1):83-85. doi:10.1136/archdischild-2019-317985

5. Hutchinson, Jo. Identifying pupils with special educational needs and disabilities. Published online 2021. Accessed October 5, 2021. https://epi.org.uk/wp-content/uploads/2021/03/SEND-Indentification_2021-EPI.pdf

6. Department for Education. 01 - Pupils in all schools, by type of SEN provision - including independent schools and general hospital schools - 2016 to 2022. Accessed January 23, 2023. https://explore-education-statistics.service.gov.uk/

7. Department for Education. Special educational needs in England, Academic Year 2021/22. Published June 16, 2022. Accessed January 23, 2023. https://explore-education-statistics.service.gov.uk/find-statistics/special-educational-needs-in-england#dataBlock-678da1e3-97e6-42f1-165e-08da47b0392d-tables

8. Hernan MA, Robins JM. *Causal Inference: What If*. Boca Raton: Chapman & Hall/CRC.; 2020.

9. Cullen MA, Lindsay G, Hastings R, Denne L, Stanford C. *Special Educational Needs in Mainstream Schools: Evidence Review*. Education Endowment Foundation; 2020. Accessed January 4, 2023. https://eric.ed.gov/?id=ED612232

10. Department for Education. The national curriculum. Accessed July 4, 2023. https://www.gov.uk/national-curriculum
