# Supplementary material for: Evaluation of variation in special educational needs provision and its impact on health and education using administrative records for England: umbrella protocol for a mixed-methods research programme
Source: BMJ Open. 2023 Nov 2;13(11):e072531. doi: 10.1136/bmjopen-2023-072531 (PMC10626865; doi:10.1136/bmjopen-2023-072531)
Supplement: Author's manuscript [file bmjopen-2023-072531.draft_revisions.pdf]

BMJ Open is committed to open peer review. As part of this commitment we make the peer review history of every article we publish publicly available.

When an article is published we post the peer reviewers' comments and the authors' responses online. We also post the versions of the paper that were used during peer review. These are the versions that the peer review comments apply to.

The versions of the paper that follow are the versions that were submitted during the peer review process. They are not the versions of record or the final published versions. They should not be cited or distributed as the published version of this manuscript.

BMJ Open is an open access journal and the full, final, typeset and author-corrected version of record of the manuscript is available on our site with no access controls, subscription charges or pay-per-view fees (http://bmjopen.bmj.com).

If you have any questions on BMJ Open's open peer review process please email info.bmjopen@bmj.com

BMJ Open

Evaluation of impact of special educational needs recording in schools on health outcomes: protocol for the Health Outcomes of young People throughout Education (HOPE) study, a mixed methods study using linked administrative education and hospital data for England

Journal:	*BMJ Open*
Manuscript ID	bmjopen-2023-072531
Article Type:	Protocol
Date Submitted by the Author:	08-Feb-2023

Complete List of Authors:	Zylbersztejn, Ania; UCL Great Ormond Street Institute of Child Health Population Policy and Practice<br>Nguyen, Vincent; UCL Great Ormond Street Institute of Child Health Population Policy and Practice<br>Matthews, Jacob; University of Cambridge, Department of Psychiatry<br>Lewis, Kate; UCL Great Ormond Street Institute of Child Health Population Policy and Practice<br>Winterburn, Isaac; University of Cambridge, Department of Psychiatry<br>Karwatowska, Lucy; UCL Great Ormond Street Institute of Child Health Population Policy and Practice<br>Barnes, Sarah; University of Cambridge, Department of Psychiatry<br>Lilliman, Matthew; UCL Great Ormond Street Institute of Child Health Population Policy and Practice<br>Saxton, Jennifer; University of Cambridge, Department of Psychiatry<br>Stone, Antony; UCL Great Ormond Street Institute of Child Health Population Policy and Practice<br>Boddy, Kate; University of Exeter Medical School, Department of Health and Community Sciences<br>Downs, Johnny; King's College London, Institute of Psychiatry, Psychology and Neuroscience; South London and Maudsley NHS Foundation Trust<br>Doyle, Martin; Riverside School<br>Logan, Stuart; University of Exeter Medical School, The Peninsula Childhood Disability Research Unit<br>Rahi, Jugnoo; UCL Great Ormond Street Institute of Child Health Population Policy and Practice; UCL Institute of Ophthalmology<br>Black-Hawkins, Kristine; University of Cambridge, Faculty of Education<br>Dearden, Lorraine; UCL Institute of Education<br>Ford, Tamsin; University of Cambridge, Psychiatry<br>Harron, Katie; UCL Great Ormond Street Institute of Child Health Population Policy and Practice<br>De Stavola, Bianca ; UCL Great Ormond Street Institute of Child Health Population Policy and Practice<br>Gilbert, Ruth; UCL Great Ormond Street Institute of Child Health Population Policy and Practice

Keywords:	EPIDEMIOLOGY, Health Equity, PUBLIC HEALTH, QUALITATIVE

	RESEARCH, STATISTICS & RESEARCH METHODS, Health informatics < BIOTECHNOLOGY & BIOINFORMATICS

SCHOLARONE™
Manuscripts

Evaluation of impact of special educational needs recording in schools on health outcomes: protocol for the Health Outcomes of young People throughout Education (HOPE) study, a mixed methods study using linked administrative education and hospital data for England

Ania Zylbersztejn[1], Vincent Nguyen[1], Jacob Matthews[2], Kate Lewis[1], Isaac Winterburn[2], Lucy Karwatowska[1], Sarah Barnes[2], Matthew Lilliman[1], Jennifer Saxton[2], Antony Stone[1], Kate Boddy[3], Johnny Downs[4,5], Martin Doyle[6], Stuart Logan[7], Jugnoo Rahi[1,8,9,10,11], Kristine Black-Hawkins[12], Lorraine Dearden[13], Tamsin Ford[2], Katie Harron[1], Bianca De Stavola[1], Ruth Gilbert[1]

[1]Population, Policy and Practice Research and Teaching Department, UCL Great Ormond Street Institute of Child Health, London, UK

[2]Department of Psychiatry, University of Cambridge, Cambridge, UK

[3]Department of Health and Community Sciences, University of Exeter Medical School

[4]Institute of Psychiatry, Psychology and Neuroscience, Kings College London, London, UK

[5]South London and Maudsley NHS Foundation Trust, London, UK

[6]Riverside School, London, UK

[7]The Peninsula Childhood Disability Research Unit, University of Exeter Medical School, Exeter, UK

[8]Great Ormond Street Hospital NHS Foundation Trust London, UK

[9]UCL Institute of Ophthalmology, London, UK

[10]NIHR Moorfields Biomedical Research Centre London, UK

[11]Ulverscroft Vision Research Group UCL London, UK

[12]Faculty of Education, University of Cambridge, Cambridge, UK

[13]UCL Institute of Education, London, UK

Corresponding author: Professor Ruth Gilbert,

Population, Policy and Practice Research and Teaching Department, UCL Great Ormond Street Institute of Child Health, 30 Guilford Street, WC1N 1EH, London, UK

r.gilbert@ucl.ac.uk

Word count: 3914/4000

For peer review only

For peer review only - http://bmjopen.bmj.com/site/about/guidelines.xhtml

Abstract

Introduction: One-third of all children in England are recorded as having special educational needs (SEN) during their school career. We hypothesise that having recorded SEN can improve health outcomes of children. SEN recording should reflect identification of need and appropriate intervention. The frequency of recorded SEN varies between schools and demographic groups, which may reflect true variations in need, inequitable provision or both. There is scant evidence about whether recorded SEN improves health outcomes.

Methods and analysis: In this mixed methods study, we will analyse linked administrative data from the Education and Health Insights from Linked Data – ECHILD database – including data from all state schools and state-funded hospitals in England, to assess inequity in recorded SEN and explore the impact of recorded SEN on health and education outcomes. Using clinical rules and data driven algorithms, we will define a range of "health phenotypes", that is groups of health conditions, expected to need SEN intervention. We will apply a range of causal inference methods to account for confounding factors and possible selection bias to draw conclusions about impact of recorded SEN, also considering timing, type or duration of SEN provision. We will use qualitative methods (surveys, interviews and focus groups with service users and providers) to understand variation in recorded SEN, the underlying processes of identification, assessment and planned intervention for SEN, and how these processes are experienced by families. We will triangulate results from these mixed methods to inform implications for policy.

Ethics and dissemination: Research ethics committees have approved the use of the ECHILD database and, separately, the survey, interviews and focus groups of young people, parents and service providers. These stakeholders will help us interpret, frame and communicate our findings to policy makers, health and education services and families in order to promote translation of our findings into practice.

Strengths and limitations of this study

Strengths:

- We use the ECHILD database, which records longitudinal histories of all National Health Service (NHS) hospital contacts and state schooling, to compare health and education outcomes in children with similar health needs according to whether or when they had SEN recorded.
- The population size (15 million) and geographic coverage of the ECHILD data allow exploration of outcomes for subgroups across different policy contexts and all areas in England.
- We apply different methods and triangulate evidence from qualitative and quantitative analyses to strengthen the robustness of our findings.

Limitations:

- Hospital records capture some but not all the reasons why children may have recorded SEN. We will not be able to identify the functional impact of health conditions or the social reasons that are not measured in ECHILD, nor SEN recording in children receiving private education.
- We can only measure health phenotypes and outcomes recorded in hospital admission records, whose quality may vary, and miss those solely recorded in primary care or mental health services.

For peer review only - http://bmjopen.bmj.com/site/about/guidelines.xhtml

Introduction

National policies in the UK and in many high-income countries require schools to make adaptations to meet the needs of children who have health, learning or behavioural problems, referred to as *special educational needs (SEN)*. In England, children with SEN "*have a significantly greater difficulty in learning than the majority of others of the same age, or have a disability which prevents them from making use of facilities generally provided by mainstream schools"* (see the study glossary in the appendix for details).[1] There are four broad areas of SENs that schools are required to support: communication and social interactions, cognition and learning, social, emotional and mental health and sensory or physical disabilities (see Box 1). Provisions and adjustments in schools due to SEN, referred to as *SEN intervention* throughout the protocol, are intended to improve inclusion and participation in education and support children's health and wellbeing.[1]

Box 1 – Broad areas of special educational needs that schools should plan for, with sub-categories set out by the Special Educational Needs and Disability code of practice in England[1]

Communication and social interactions
- Speech, language and communication needs
- Autistic Spectrum Disorders

Cognition and learning:
- Moderate learning difficulties
- Severe learning difficulties
- Profound and multiple learning difficulties
- Specific learning difficulties (that is learning difficulties affecting one or more specific aspects of learning, such as dyslexia, dyscalculia or dyspraxia)

Social, emotional and mental health

Sensory and/or physical disabilities:
- Vision impairment
- Hearing impairment
- Multi-sensory impairment
- Physical disability requiring additional ongoing support and/or equipment to access all the opportunities available to their peers

Each year, approximately one in six children in England are recorded by schools as having SEN[2] and one-third of all children have SEN recorded at least once during their time in school.[3,4] The intensity and type of SEN intervention vary. Around 4% of all children have a record of an *Education and Health Care Plan (EHCP)*, which reflects relatively intensive intervention arranged and funded by local authorities.[2,5] An EHCP involves a formal assessment of needs and a legal document setting out special measures provided by local authorities to meet a child's needs across education, health and social care. For some children an EHCP may cover support up until their 25th birthday. Support may range from extra help by a part-time teaching assistant to full-time care by multiple staff in a special school.[1] Approximately half of the children with an EHCP are enrolled in a special school (1.5% of all children).[2,5] Many more children have a record of *SEN support* which includes less intensive provision arranged and funded by the school and provided almost entirely in mainstream schools. SEN support can be a short-term intervention such as speech or language therapy or extra support for reading.

For peer review only - http://bmjopen.bmj.com/site/about/guidelines.xhtml

Schools report information on identified children with SEN who require additional support in termly school censuses returned to the Department for Education. We refer to this as *recorded SEN* throughout the protocol. Recorded SEN should indicate identification, assessment, and appropriate intervention for a specific need (e.g., speech, language, and communication needs, see Box 1). Although schools record which children are identified are needing SEN provision, the school census does not include information on what (if any) SEN intervention is received and whether it is considered to be appropriate.[6]

The frequency of recorded SEN varies between schools, regions and those experiencing deprivation and is widely regarded as unfair and inequitable.[2,7,8] Recorded SEN has declined in England since 2010 and is strongly determined by school, local authority and Department for Education policies, including funding arrangements (see Figure 1).[4] School-level factors (such as academisation), and wider social factors (such as deprivation, ethnic group, child's first language, contacts with social care) also appear to affect SEN recording, but evidence is limited to specific types of SEN recorded in school census (for example, autism, see Box 1).[3,7–11]

Compared to their peers, children with recorded SEN have higher rates of chronic physical and mental health conditions, hospitalisations, and have lower self-reported wellbeing.[12–14] SEN intervention is likely to improve health outcomes, although there is little evidence linking SEN intervention to health outcomes or healthcare utilisation. Classroom-based SEN interventions can improve children's social and emotional wellbeing and reduce challenging behaviour,[15,16] contributing to better mental health outcomes. For children with attention deficit hyperactivity disorder (ADHD), systematic reviews of randomised, or quasi randomised controlled trials (RCTs) of interventions similar to SEN interventions show improvements in behaviour.[12,17–19] Children receiving SEN intervention may also receive additional support with managing their co-existing chronic health needs, which may contribute to better health outcomes. Children in special schools may benefit from physiotherapy, speech and language therapy or on-site visit from community paediatrician, increasing the odds of early detection of emerging health problems. However, there is a lack of RCTs or robust observational comparisons that evaluate the impact of SEN intervention on health outcomes.

Robust evidence that SEN intervention effectively improves educational outcomes for pupils with SEN is also scarce.[20] There is moderate evidence that SEN interventions in primary schools improve literacy difficulties, socio-emotional development and language and communication.[15] A recent evidence review found a weak but positive impact of inclusive education on academic outcomes of pupils with no SEN.[20] Evidence from population-based observational studies suggests that recorded SEN is associated with fewer absences and exclusions in children and young people with neurodisabilities and mental health conditions.[21]

Given the large proportion of children with recorded SEN, the high costs of the SEN system, and increasing pressure on schools due to funding constraints,[2] evidence is needed to guide effective and equitable SEN interventions to groups of children most likely to benefit. SEN intervention may improve health and education outcomes more for children with certain types of health conditions, or social context, than others. Evidence that applies similar methods of evaluation across a range of subgroups of children with different health conditions (or phenotypes) and in different social contexts is needed to identify what works for whom.

This study aims to address these gaps in evidence by assessing whether access to SEN intervention is equitable, whether SEN intervention is effective and which type and timing of intervention works for whom. We will use the Education and Child Health Insights from Linked Data (ECHILD) database, which combines routinely collected administrative data from education, social care and publicly funded hospitals for all children in England over two decades.[22] We will explore which causal inference methods can be used to provide valid evidence of the impact of SEN intervention on health and education outcomes, and in which contexts.

For peer review only - http://bmjopen.bmj.com/site/about/guidelines.xhtml

We consulted children and young people who chose to name this study HOPE: Health Outcomes of young People in Education (HOPE).

Methods and analysis

Study design

The HOPE Study involves mixed qualitative and quantitative methods and input from key stakeholders (practitioners, managers and policy makers, education and health staff, parents/carers and young people). The study started in August 2021 and is expected to end in June 2024. The study involves four closely linked work packages (WPs), which underpin different parts of the conceptual framework for the study (illustrated in Figure 2).

In WP1, we will focus on defining the **population** of children who would stand to benefit from SEN intervention. We will define and validate *health phenotypes* associated with SEN intervention, that is groups of children with similar health conditions, who are expected to have higher need for SEN intervention than their peers. We will also define **outcomes** likely to be affected by SEN intervention. Analyses will explore whether there is evidence of **inequity** in SEN provision (measured by school records) across a range of phenotypes, and what child-level factors might be associated with it.

WP2 will examine factors associated with **variation in SEN** recording and provision using qualitative and quantitative methods. We will generate evidence on why and how recorded SEN varies across schools and local authorities in England, how the underlying processes of identification, assessment and intervention vary, and how these processes are experienced by families.

In WPs 3-4 we will use causal inference methods to assess the **impact** of recorded SEN on outcomes. WP3 will examine the impact of recorded SEN at a given point during school, e.g. in year 1. WP4 will assess the impact of the timing and duration of recorded SEN. We will apply different methods to account for **confounding factors**, that is sources of non-causal associations. Findings from WP1 and WP2 will inform the choice of causal methods to be used in WPs3 and 4, e.g. based on instrumental variables or propensity score-based control of measured confounders.

Data sources

ECHILD database

The ECHILD database links routinely collected administrative data on health and education in England.[22] Currently ECHILD follows all children and young people aged 0–24 years in England who were born between 1 September 1995 and 31 August 2021 (approximately 14.7 million individuals), and data collection is ongoing.[22] Health and education datasets were linked using a multi-step deterministic linkage algorithm. Linkage rates were high and increased over time (from 92% of school pupils born in academic year 1990/91 linking to at least one hospital record, to 99% of those born 2004/05).[23]

Health data comes from Hospital Episode Statistics (HES), a national database that includes dated information on all National Health Service (NHS) hospital care (including all admissions to hospitals from April 1997, outpatient appointments from April 2002 and accident and emergency (A&E) department attendances from April 2006) and mortality data (see Figure 3 for details of data coverage by academic year).[24] Information about children born in England and their birth characteristics can be obtained from recorded birth admissions.[25,26]

Education records are collated in the National Pupil Database (NPD) and include information on registration in school, school attainment scores at ages 5, 7 11, 16 and 18, and number of half-day absences and exclusions in each 13-week term. SEN is recorded each term (annually prior to 2005/6) for all children in state-funded education from the academic year starting in September 2001 onwards (see Figure 3).[6] Education data captures the level of recorded SEN intervention (EHCP or

For peer review only - http://bmjopen.bmj.com/site/about/guidelines.xhtml

SEN support) and main reason for the intervention. Most recent categories reflect language or communication, moderate or severe learning disability, autism, sensory impairment, physical disability, or social, emotional or mental health needs (see Box 1 for details). ECHILD also includes linked social care services records.[22,27,28] We will enhance ECHILD with school characteristics, such as type of school (e.g.: mainstream or special) or teacher-pupil ratio, from publicly available data.[29]

National online survey and qualitative interviews

We will conduct an online survey for service users and providers (including children, young people, parents/carers and education, health, education and local authority professionals) as part of WP2. The survey will collect data on local experiences of identification and assessment of need and provision of interventions.

We will also carry out qualitative interviews and focus groups to elicit perspectives of service users and providers about experience of the SEN process and which outcomes might be affected. These data will be synthesised with quantitative analyses to understand variation in SEN recording in the ECHILD database and key factors that are not captured by the administrative data.

Methods

Study populations

We will define health phenotypes, that is sub-populations of children who stand to benefit from SEN intervention because of additional health needs or conditions. Because indications of SEN used in education settings (such as learning disability) are likely to be under-recorded in hospital records,[30,31] we will use clinical evidence from the research literature, clinical experts, service users and providers, to identify health conditions likely to result in impairment or behaviours that meet criteria for SEN intervention. These phenotypes will be characterised by combinations of diagnostic codes indicating specific health conditions.[31–33] The phenotypes associated with SEN may be recorded in health records from birth (e.g. Down syndrome or other congenital anomalies,[34,35] or preterm birth[13]), throughout childhood (e.g.: cerebral palsy,[36] epilepsy[37]) or in adolescence (e.g.: mental health conditions[38]).

Where possible, we will define subgroups of children within each health phenotype, who are more severely affected or are expected to have more functional impairment and increased need for SEN intervention. For example, for phenotypes involving congenital anomalies, we will distinguish those with a more extensive malformation (e.g.: cleft lip and palate vs cleft lip alone), or multiple malformations (e.g.: Down syndrome with or without congenital heart malformation), to create sub-phenotypes with similar levels of SEN. Where clinically relevant, we will also explore indicators of severity of health needs recorded through childhood such as the frequency, duration or regularity of hospital contacts and measures of cognitive ability and behaviour reflected in school attainment tests, such as the EYFSP at age 5, key stage 1 at age 7, and key stage 2 at age 11.[4]

We will also consult stakeholders (including children, young people and their parents/carers) about the key points during school years when SEN intervention might have impact. Our public engagement activities suggest that the key time points include: start of primary school; transition to secondary school; and the final years of school, when young people take important public exams. This will inform how we derive the study cohorts (e.g. defining inception to the cohort, length of follow-up, and "look-back" windows for identifying health conditions) for each key time point.

Outcomes

We will synthesise findings from literature reviews and stakeholder consultation to define which health outcomes are likely to be impacted by SEN intervention. Our primary focus will be on outcomes related to health, which can be measured through contacts with NHS hospital services.

For peer review only - http://bmjopen.bmj.com/site/about/guidelines.xhtml

We hypothesise that SEN intervention may reduce unmet health need and stress and thereby reduce unplanned contacts with hospital, measured as unplanned admissions or visits to the A&E. We will analyse unplanned admissions due to specific causes that might reflect stress at school (e.g. mental health, stress-related presentations,[39] or self-harm, violence, drug or alcohol misuse in adolescence[40]), or due to complications of chronic conditions that might be prevented given additional support for managing health conditions at school.

Indicators of secondary health outcomes will include planned healthcare contacts (hospital admissions or outpatient visits). Planned healthcare might increase or decrease due to SEN intervention for some health phenotypes, we will therefore describe these outcomes to generate further hypotheses. An increase in planned healthcare contacts could reflect support through SEN intervention for engagement with health care, whereas a decrease in planned healthcare contacts might reflect proactive care, such as physiotherapy or help with medication in school, reducing need for hospital visits. Total mortality will be reported as an additional outcome.

As SEN interventions aim to improve inclusion, we will also analyse outcomes linked to participation in education, including rates of absence, exclusion, changing schools, and non-enrolment (potentially indicating off-rolling or 'pushing out' from school).[41] We will also analyse rates of participation in attainment tests at key stages (ages 5, 7, 11, 16 and 18 years old) and differences in attainment scores, according to SEN intervention status.

Exploring variation in recorded SEN

The ECHILD database contains records of SEN, but no information about the timing, type or quality of the SEN intervention received. Recorded SEN indicates a process of identification and assessment of need and intervention (see Figure 2). We will involve service users and providers, using qualitative methods to understand how these processes vary. First, we will analyse publicly available documents on the support available for local children with SEN ("local offer") and reports from inspections examining how effectively local authorities fulfil responsibilities for children and young people with SEN.

Second, as described in Study Design, we will design a quantitative national survey for key service users and providers (including children and young people, parents/carers and education, health and local authority professionals) which explores their local experiences of identification of need, and allocation and receipt of SEN intervention. We will conduct qualitative interviews with parents and young people and focus groups with service providers to explore varying criteria for identifying, assessing and providing SEN intervention and how these processes affect recording of SEN, and elicit perspectives of service users on their experiences of the SEN process. These qualitative findings will be triangulated with findings from the ECHILD analyses in a systems analysis of organisational factors affecting SEN recording and intervention.

Third, we will analyse the frequency of recorded SEN for different health phenotypes and explore variation according to co-existing health needs, social factors, demographic characteristics and organisational factors linked to schools and local authorities. Recorded SEN varies according to child's demographic and socioeconomic characteristics. For example, boys are more likely to have recorded SEN than girls,[5] numerous reports show disproportionate identification of SEN for pupils from ethnic minority groups,[7,8,10,11] and 85% of children who have been looked after in out-of-home care receive SEN support in England.[3]

Fourth, we will look at how recorded SEN varies over geographical area and time. Recorded SEN is not evenly distributed across schools, local authorities or regions and the levels of provision has changed over time (Figure 1). The annual (cross-sectional) prevalence of any SEN provision peaked in 2010 at 20% of children, and declined to 14% in 2016, in part because of the change in policy – the Children and Families Act in 2014 and Special Educational Needs and Disability Code of Practice implemented in 2015. We will examine how recorded SEN changed over time for children with

For peer review only - http://bmjopen.bmj.com/site/about/guidelines.xhtml

different health phenotypes, and the percentage of variation that is explained by factors at the individual-, school- and local authority-level.

Assessing the impact of recorded SEN on outcomes.

We will use a range of biostatistical and econometric methods to explore the impact of recorded SEN on outcomes across a range of health phenotypes. We will address the impact of recorded SEN at a given point in time, for example by comparing outcomes for pupils with recorded SEN versus none, or early versus deferred provision. We will use econometric methods that try to deal with unmeasured confounding by finding circumstances that create a natural experiment or instruments (exogenous drivers), that systematically change SEN intervention while not changing factors that could affect the outcome of interest apart from through SEN intervention. This could be due to changes in policy or funding, or policy variation between areas or schools.

Secondly, we will assess the impact of the duration of SEN intervention, appropriately controlling for likely time-varying factors that may be affected by SEN intervention and also influence future SEN provision. To avoid biases in the design stage of these investigations, such as immortal time bias and prevalent-case bias,[42] we will use the target trial emulation (TTE) framework to guide the creation of study cohorts that correspond to the various phenotypes and exposure levels (SEN intervention) to be compared. TTE consists of first designing an ideal pragmatic trial that would address the question of interest, and then emulate it as closely as possible using observational data. The analysis of these data, unlike those of the ideal trial, would nevertheless need to address issues of confounding using various methods from the causal inference literature, including g-methods and dynamic panel modelling.[43,44]

As we use observational data, a major challenge will be to address confounding. Potential confounders will vary between settings. Demographic data on sex, age, ethnic group and measures of socioeconomic circumstances (such as area-level deprivation, eligibility for free school meals) are recorded in health and education datasets. Baseline health data e.g. on other chronic health conditions or frequency of hospital contacts and baseline education data e.g. academic attainment scores, absences or exclusions (as measured at the start of follow-up, which could be at school year 1 (age 5) or year 6 (age 10) depending on the question) could also be used to control for the severity of the health phenotype. Some confounding factors will be unmeasured (e.g. disruptive behaviour, severity of the condition, safeguarding concerns, or ability of the parents to navigate the application system). We will therefore explore different ways to account for measured and unmeasured confounding.

Study size

We will analyse data for all children in England. Between 1st September 2018 and 31st August 2019 there were approximately 550,000 children in each school year, including those entering reception class at age 4 to 5, up until year 11 at age 15 to 16. As these numbers are large, we can focus on specific or even uncommon phenotypes. For example, we will be able to study approximately 5000 children with cleft lip and palate of whom we expect one third to have recorded SEN.

Impact of COVID pandemic

The COVID-19 pandemic had a significant impact on the wellbeing of young people.[45] Lockdowns in England affected children's access to school and the frequency of hospital contacts captured in ECHILD data. Planned and unplanned admissions, and outpatient appointments reduced substantially during the COVID-19 pandemic, with largest reductions in children with indicators of vulnerability (such as preterm birth, a chronic condition, recorded SEN or social care record).[14,46] School closures led to fewer stress-related presentations to health services, although rates returned

For peer review only - http://bmjopen.bmj.com/site/about/guidelines.xhtml

to pre-pandemic levels after re-opening, and increased for girls aged 11-15 years old.[47] To account for the impact of the COVID-19 pandemic we will use the ECHILD database to describe changes in health and education outcomes, including participation in schooling, in children with health phenotypes expected to need SEN intervention and comparator cohorts, before and after the onset of COVID-19.

Public involvement and engagement

The HOPE study was developed in response to consultations about the need for the ECHILD database with parents and charities supporting children with chronic health conditions and their families.[48,49] The study will establish groups of service users (parents and young people) across England, coordinated by co-investigators working on WP2. Parents/carers and young people will be consulted about their experiences of the SEN process, asked to complete an online survey, and invited to participate in interviews. We will collaborate with staff in schools to enable young people with additional learning needs or disabilities to contribute. We will also present our planned analyses and preliminary results to parents/carers and young people through research advisory groups at UCL and University of Exeter. These consultations will contribute evidence to all four work packages and dissemination of the research. The HOPE Study Steering Committee includes two parents of children with disability and will adhere to NIHR requirements for payment for time and expenses of lay contributors.

Ethics

Existing research ethics approval has been granted for analyses of the ECHILD database for the purposes set out in the HOPE study (20/EE/0180). Data access is also controlled by agreements with NHS Digital and the Department for Education. The data contain no identifiers or sensitive dates (e.g. date of birth) and data can only be used within the Office for National Statistics Secure Research Environment by approved researchers, with strict statistical disclosure controls of all outputs of analyses (e.g. tables or figures). Details are published here in our privacy notice (https://www.ucl.ac.uk/child-health/research/population-policy-and-practice-research-and-teaching-department/cenb-clinical-4#).

Separate ethics approval has been approved for research involving service users (young people and parents) and service providers (PRE:2021.058).

Dissemination

We will present preliminary findings to diverse audiences (academics, analysts at Department for Education and Department of Health and Social Care, and stakeholders of service users and providers) through seminars, question and answer sessions, workshops and consultations during the study. We will incorporate feedback into final outputs, which will include peer reviewed journal articles, the final study report to funder, and short briefing reports and infographics for non-academics published on the study website.

We will publish our methods and code to enable others to reproduce and extend our analyses using ECHILD. ECHILD Database will be made available for research for public benefit in 2023. Meta-data and code relating to the HOPE study will be signposted on the study website and made available in the ONS secure environment and in code repository (e.g. on GitHub). We will hold workshops to promote wider use of findings from the HOPE study for causal analyses of education interventions on health. Examples from the HOPE study will be incorporated into short courses on causal methods and on how to use the ECHILD database.

The HOPE study aims to build the evidence base for fairer and more effective SEN provision and, by informing national and local policy and the public and changing practice, to improve health and education outcomes of children with SEN.

For peer review only - http://bmjopen.bmj.com/site/about/guidelines.xhtml

For peer review only

For peer review only - http://bmjopen.bmj.com/site/about/guidelines.xhtml

Footnotes

Abbreviations

A&E: accident and emergency department

ADHD: attention deficit hyperactivity disorder

ASD: autistic spectrum disorders

ECHILD: Education and Child Health Insights from Linked Data

EHCP: education, health and care plan

HES: Hospital Episode Statistics

NHS: National Health Service

NPD: National Pupil Database

RCT: randomised controlled trial

SEN: special educational needs

TTE: target trial emulation

WP: work package

Author contributions

RG is the principal investigator of the study and drafted the original protocol, with input from KH, TF, BDS, LD, KBH, JR, JD and SL. AZ and RG produced the first draft of this manuscript; AZ, KL and ML created figures, AZ, VN and KL prepared the glossary, all authors edited the manuscript. All authors have contributed to and are responsible for the final design of the study. All authors read and approved the final manuscript.

Acknowledgments

We would also like to acknowledge the contribution of members of the HOPE study steering committee.

Competing Interests

We declare no competing interests.

Funding

This study/project is funded by the National Institute for Health Research (NIHR) under its Programme Grants for Applied Research Programme (Grant Reference number NIHR202025). The views expressed are those of the author(s) and not necessarily those of the NIHR or the Department of Health and Social Care.

Patient and public involvement

Patients and/or the public were involved in the design, or conduct, or reporting, or dissemination plans of this research. Refer to the Methods section for further details.

For peer review only - http://bmjopen.bmj.com/site/about/guidelines.xhtml

References

(1) Department for Education. Special Educational Needs and Disability Code of Practice: 0 to 25 Years. **2015**. https://doi.org/DFE-00205-2013.

(2) The National Audit Office. Support for Pupils with Special Educational Needs and Disabilities in England. **2019**.

(3) Jay, M. A.; Gilbert, R. Special Educational Needs, Social Care and Health. *Arch. Dis. Child.* **2021**, *106* (1), 83–85. https://doi.org/10.1136/archdischild-2019-317985.

(4) Hutchinson, Jo. Identifying Pupils with Special Educational Needs and Disabilities. **2021**.

(5) Department for Education. *Special educational needs in England, Academic Year 2021/22*. https://explore-education-statistics.service.gov.uk/find-statistics/special-educational-needs-in-england#dataBlock-678da1e3-97e6-42f1-165e-08da47b0392d-tables (accessed 2023-01-23).

(6) Jay, M. A.; Grath-Lone, L. M.; Gilbert, R. Data Resource: The National Pupil Database (NPD). *Int. J. Popul. Data Sci.* **2019**, *4* (1). https://doi.org/10.23889/ijpds.v4i1.1101.

(7) Roman-Urrestarazu, A.; van Kessel, R.; Allison, C.; Matthews, F. E.; Brayne, C.; Baron-Cohen, S. Association of Race/Ethnicity and Social Disadvantage With Autism Prevalence in 7 Million School Children in England. *JAMA Pediatr.* **2021**, *175* (6), e210054. https://doi.org/10.1001/jamapediatrics.2021.0054.

(8) Strand, S.; Lindorff, A. Ethnic Disproportionality in the Identification of Special Educational Needs (SEN) in England: Extent, Causes and Consequences.

(9) Black, A.; Bessudnov, A.; Liu, Y.; Norwich, B. Academisation of Schools in England and Placements of Pupils With Special Educational Needs: An Analysis of Trends, 2011–2017. *Front. Educ.* **2019**, *4*.

(10) Strand, S.; Lindorff, A. Ethnic Disproportionality in the Identification of High-Incidence Special Educational Needs: A National Longitudinal Study Ages 5 to 11. *Except. Child.* **2021**, *87* (3), 344–368. https://doi.org/10.1177/0014402921990895.

(11) Schmaus, M. Disproportionate Identification of Special Needs for Ethnic and Language Minority Students in England–Patterns and Explanations. *Br. J. Sociol. Educ.* **2022**, *43* (1), 40–62. https://doi.org/10.1080/01425692.2021.2002682.

(12) Barnes, M.; Harrison, E. The Wellbeing of Secondary School Pupils with Special Educational Needs: Reserach Report. *Dep. Educ.* **2017**.

(13) Libuy, N.; Gilbert, R.; Mc Grath-Lone, L.; Blackburn, R.; Etoori, D.; Harron, K. Gestational Age at Birth, Chronic Conditions and School Outcomes: A Population-Based Data Linkage Study of Children Born in England. *Int. J. Epidemiol.* **2022**, dyac105. https://doi.org/10.1093/ije/dyac105.

(14) Mc Grath-Lone, L.; Etoori, D.; Gilbert, R.; Harron, K. L.; Woodman, J.; Blackburn, R. Changes in Adolescents' Planned Hospital Care during the COVID-19 Pandemic: Analysis of Linked Administrative Data. *Arch. Dis. Child.* **2022**, *107* (10), e29–e29. https://doi.org/10.1136/archdischild-2021-323616.

(15) Carroll, J.; Bradley, L.; Hannant, P.; Thompson, A. SEN Support: A Rapid Evidence Assessment.

(16) Cullen, M. A.; Lindsay, G.; Hastings, R.; Denne, L.; Stanford, C.; Beqiraq, L.; Elahi, F.; Gemegah, E.; Hayden, N.; Kander, I.; Lykomitrou, F.; Zander, J. Special Educational Needs in Mainstream Schools: Evidence Review.

For peer review only

(17) Moore, D. A.; Russell, A. E.; Matthews, J.; Ford, T. J.; Rogers, M.; Ukoumunne, O. C.; Kneale, D.; Thompson-Coon, J.; Sutcliffe, K.; Nunns, M.; Shaw, L.; Gwernan-Jones, R. School-Based Interventions for Attention-Deficit/Hyperactivity Disorder: A Systematic Review with Multiple Synthesis Methods. *Rev. Educ.* **2018**, *6* (3), 209–263. https://doi.org/10.1002/rev3.3149.

(18) Moore, D.; Benham-Clarke, S.; Kenchington, R.; Boyle, C.; Ford, T.; Hayes, R.; Rogers, M. *Improving Behaviour in Schools: Evidence Review*; Education Endowment Foundation, 2019. https://eric.ed.gov/?id=ED612210 (accessed 2023-01-05).

(19) Richardson, M.; Moore, D. A.; Gwernan-Jones, R.; Thompson-Coon, J.; Ukoumunne, O.; Rogers, M.; Whear, R.; Newlove-Delgado, T. V.; Logan, S.; Morris, C.; Taylor, E.; Cooper, P.; Stein, K.; Garside, R.; Ford, T. J. Non-Pharmacological Interventions for Attention-Deficit/Hyperactivity Disorder (ADHD) Delivered in School Settings: Systematic Reviews of Quantitative and Qualitative Research. *Health Technol. Assess. Winch. Engl.* **2015**, *19* (45), 1–470. https://doi.org/10.3310/hta19450.

(20) Cullen, M. A.; Lindsay, G.; Hastings, R.; Denne, L.; Stanford, C. *Special Educational Needs in Mainstream Schools: Evidence Review*; Education Endowment Foundation, 2020. https://eric.ed.gov/?id=ED612232 (accessed 2023-01-04).

(21) John, A.; Friedmann, Y.; DelPozo-Banos, M.; Frizzati, A.; Ford, T.; Thapar, A. Association of School Absence and Exclusion with Recorded Neurodevelopmental Disorders, Mental Disorders, or Self-Harm: A Nationwide, Retrospective, Electronic Cohort Study of Children and Young People in Wales, UK. *Lancet Psychiatry* **2022**, *9* (1), 23–34. https://doi.org/10.1016/S2215-0366(21)00367-9.

(22) Mc Grath-Lone, L.; Libuy, N.; Harron, K.; Jay, M. A.; Wijlaars, L.; Etoori, D.; Lilliman, M.; Gilbert, R.; Blackburn, R. Data Resource Profile: The Education and Child Health Insights from Linked Data (ECHILD) Database. *Int. J. Epidemiol.* **2022**, *51* (1), 17–17f. https://doi.org/10.1093/ije/dyab149.

(23) Libuy, N.; Harron, K.; Gilbert, R.; Caulton, R.; Cameron, E.; Blackburn, R. Linking Education and Hospital Data in England: Linkage Process and Quality. *Int. J. Popul. Data Sci.* **2021**, *6* (1). https://doi.org/10.23889/ijpds.v6i1.1671.

(24) Herbert, A.; Wijlaars, L.; Zylbersztejn, A.; Cromwell, D.; Hardelid, P. Data Resource Profile: Hospital Episode Statistics Admitted Patient Care (HES APC). *Int. J. Epidemiol.* **2017**, *46* (4), 1093–1093i. https://doi.org/10.1093/ije/dyx015.

(25) Zylbersztejn, A.; Gilbert, R.; Hardelid, P. Developing a National Birth Cohort for Child Health Research Using a Hospital Admissions Database in England: The Impact of Changes to Data Collection Practices. *PLOS ONE* **2020**, *15* (12), e0243843. https://doi.org/10.1371/journal.pone.0243843.

(26) Harron, K.; Gilbert, R.; Cromwell, D.; Meulen, J. van der. Linking Data for Mothers and Babies in De-Identified Electronic Health Data. *PLOS ONE* **2016**, *11* (10), e0164667. https://doi.org/10.1371/journal.pone.0164667.

(27) Mc Grath-Lone, L.; Harron, K.; Dearden, L.; Nasim, B.; Gilbert, R. Data Resource Profile: Children Looked After Return (CLA). *Int. J. Epidemiol.* **2016**, *45* (3), 716–717f. https://doi.org/10.1093/ije/dyw117.

(28) Emmott, E. H.; Jay, M. A.; Woodman, J. Cohort Profile: Children in Need Census (CIN) Records of Children Referred for Social Care Support in England. *BMJ Open* **2019**, *9* (2), e023771. https://doi.org/10.1136/bmjopen-2018-023771.

(29) Department for Education. *Get Information about Schools (GIAS)*. https://www.get-information-schools.service.gov.uk/ (accessed 2023-01-05).

For peer review only - http://bmjopen.bmj.com/site/about/guidelines.xhtml

(30) Bourke, J.; Wong, K.; Leonard, H. Validation of Intellectual Disability Coding through Hospital Morbidity Records Using an Intellectual Disability Population-Based Database in Western Australia. *BMJ Open* **2018**, *8* (1), e019113. https://doi.org/10.1136/bmjopen-2017-019113.

(31) Zylbersztejn, A.; Stilwell, P. A.; Zhu, H.; Ainsworth, V.; Allister, J.; Horridge, K.; Stephenson, T.; Wijlaars, L.; Gilbert, R.; Heys, M.; Hardelid, P. Trends in Hospital Admissions during Transition from Paediatric to Adult Services for Young People with Learning Disabilities or Autism: Population-Based Cohort Study. *Lancet Reg. Health – Eur.* **2023**, *24*. https://doi.org/10.1016/j.lanepe.2022.100531.

(32) Zylbersztejn, A.; Verfürden, M.; Hardelid, P.; Gilbert, R.; Wijlaars, L. Phenotyping Congenital Anomalies in Administrative Hospital Records. *Paediatr. Perinat. Epidemiol.* **2020**, *34* (1), 21–28. https://doi.org/10.1111/ppe.12627.

(33) Hardelid, P.; Dattani, N.; Gilbert, R. Estimating the Prevalence of Chronic Conditions in Children Who Die in England, Scotland and Wales: A Data Linkage Cohort Study. *BMJ Open* **2014**, *4* (8), e005331. https://doi.org/10.1136/bmjopen-2014-005331.

(34) Glinianaia, S. V.; McLean, A.; Moffat, M.; Shenfine, R.; Armaroli, A.; Rankin, J. Academic Achievement and Needs of School-Aged Children Born with Selected Congenital Anomalies: A Systematic Review and Meta-Analysis. *Birth Defects Res.* **2021**, *113* (20), 1431–1462. https://doi.org/10.1002/bdr2.1961.

(35) Petterson, B.; Bourke, J.; Leonard, H.; Jacoby, P.; Bower, C. Co-Occurrence of Birth Defects and Intellectual Disability. *Paediatr. Perinat. Epidemiol.* **2007**, *21* (1), 65–75. https://doi.org/10.1111/j.1365-3016.2007.00774.x.

(36) Reid, S. M.; Meehan, E. M.; Arnup, S. J.; Reddihough, D. S. Intellectual Disability in Cerebral Palsy: A Population-Based Retrospective Study. *Dev. Med. Child Neurol.* **2018**, *60* (7), 687–694. https://doi.org/10.1111/dmcn.13773.

(37) Fleming, M.; Fitton, C. A.; Steiner, M. F. C.; McLay, J. S.; Clark, D.; King, A.; Mackay, D. F.; Pell, J. P. Educational and Health Outcomes of Children and Adolescents Receiving Antiepileptic Medication: Scotland-Wide Record Linkage Study of 766 244 Schoolchildren. *BMC Public Health* **2019**, *19* (1), 595. https://doi.org/10.1186/s12889-019-6888-9.

(38) Fleming, M.; Fitton, C. A.; Steiner, M. F. C.; McLay, J. S.; Clark, D.; King, A.; Mackay, D. F.; Pell, J. P. Educational and Health Outcomes of Children and Adolescents Receiving Antidepressant Medication: Scotland-Wide Retrospective Record Linkage Cohort Study of 766 237 Schoolchildren. *Int. J. Epidemiol.* **2020**, *49* (4), 1380–1391. https://doi.org/10.1093/ije/dyaa002.

(39) Blackburn, R.; Ajetunmobi, O.; Mc Grath-Lone, L.; Hardelid, P.; Shafran, R.; Gilbert, R.; Wijlaars, L. Hospital Admissions for Stress-Related Presentations among School-Aged Adolescents during Term Time versus Holidays in England: Weekly Time Series and Retrospective Cross-Sectional Analysis. *BJPsych Open* **2021**, *7*. https://doi.org/10.1192/bjo.2021.1058.

(40) Herbert, A.; Gilbert, R.; Cottrell, D.; Li, L. Causes of Death up to 10 Years after Admissions to Hospitals for Self-Inflicted, Drug-Related or Alcohol-Related, or Violent Injury during Adolescence: A Retrospective, Nationwide, Cohort Study. *The Lancet* **2017**, *390* (10094), 577–587. https://doi.org/10.1016/S0140-6736(17)31045-0.

(41) Jay, M. A.; Grath-Lone, L. M.; De Stavola, B.; Gilbert, R. Evaluation of Pushing out of Children from All English State Schools: Administrative Data Cohort Study of Children Receiving Social Care and Their Peers. *Child Abuse Negl.* **2022**, *127*, 105582. https://doi.org/10.1016/j.chiabu.2022.105582.

For peer review only - http://bmjopen.bmj.com/site/about/guidelines.xhtml

(42) Hernán, M. A.; Robins, J. M. Using Big Data to Emulate a Target Trial When a Randomized Trial Is Not Available. *Am. J. Epidemiol.* **2016**, *183* (8), 758–764. https://doi.org/10.1093/aje/kwv254.

(43) Blundell, R.; Bond, S. Initial Conditions and Moment Restrictions in Dynamic Panel Data Models. *J. Econom.* **1998**.

(44) Daniel, R. m.; Cousens, S. n.; De Stavola, B. l.; Kenward, M. G.; Sterne, J. a. C. Methods for Dealing with Time-Dependent Confounding. *Stat. Med.* **2013**, *32* (9), 1584–1618. https://doi.org/10.1002/sim.5686.

(45) Office for Health Improvement and Disparities. 4. Children and Young People. In *COVID-19 mental health and wellbeing surveillance: report*; 2022.

(46) Etoori, D.; Harron, K. L.; Mc Grath-Lone, L.; Verfürden, M. L.; Gilbert, R.; Blackburn, R. Reductions in Hospital Care among Clinically Vulnerable Children Aged 0–4 Years during the COVID-19 Pandemic. *Arch. Dis. Child.* **2022**, *107* (10), e31–e31. https://doi.org/10.1136/archdischild-2021-323681.

(47) Blackburn, R. M.; Owen, J. P.; Downs, J.; Gilbert, R. COVID-19-Related School Closures and Patterns of Hospital Admissions with Stress-Related Presentations in Secondary School-Aged Adolescents: Weekly Time Series. *Br. J. Psychiatry* **2022**, *221* (5), 655–657. https://doi.org/10.1192/bjp.2022.113.

(48) UCL. *The ECHILD project website*. https://www.ucl.ac.uk/child-health/research/population-policy-and-practice-research-and-teaching-department/cenb-clinical-20 (accessed 2023-01-19).

(49) Waind, E.; Mc Grath-Lone, L. The Education & Child Health Insights from Linked Data (ECHILD) Database Key Messages from Stakeholders. *ADR UK* **2021**.

For peer review only - http://bmjopen.bmj.com/site/about/guidelines.xhtml

Figure 1 – Percentage of total children with recorded SEN per school year, January 2003 to 2022

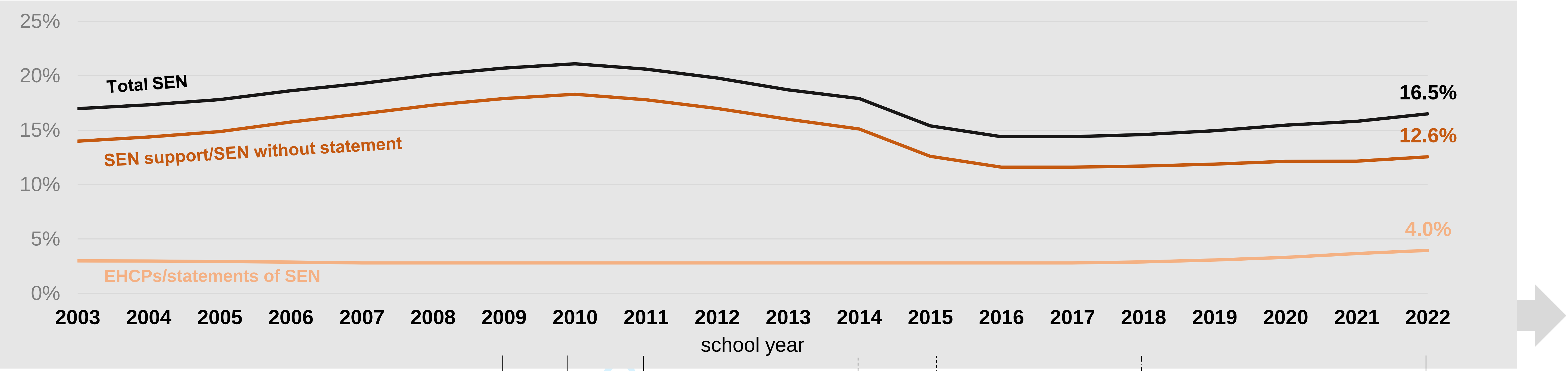

The Special Educational Needs and Disability Act 2001: legislation on rights of disabled and special educational needs (SEN) children in compulsory and post-16 education, training and other student services

Special Educational Needs Code of Practice: statutory guidance for Local Authorities, maintained schools, early years settings and others on carrying out their statutory duties to identify, assess and make provision for children's special educational needs (SEN); effective from **1 January 2002**

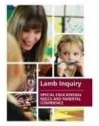

2009: Lamb enquiry
UK Government commissioned enquiry focusing on improving parental confidence in the SEN system

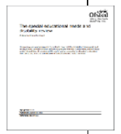

2010: Special education needs and disability review: a statement is not enough
The Office for Standards in Education, Children's Services and Skills (Ofsted) was commissioned by UK Government to evaluate how well the legislative framework serves children with SEN. Review identified wide variation in the identification of SEN and provision across schools and local authorities and argued that there is an over identification of SEN in lieu of good teaching.

2010: Salt review
Independent review of teaching for children with severe/profound and multiple learning disabilities

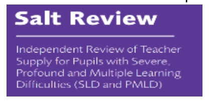

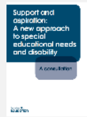

2011: Support and Aspiration: A new approach to SEND

Consultation and subsequent green paper (2012). Confirmed a focus on early identification and a better experience for parents with less 'fighting for' support, and better coordination between health, social care and education services.

2014: Children and Families Act

Legislation on rights of children, families, and people with special educational needs or disabilities

2015: SEND Code of Practice: 0 to 25 years

statutory guidance for organisations working with young people with SEND. Introduced Education, Health and Care Plans (EHCPs) instead of Statements of SEN.

2018: Deadline for Local authorities to **replace statements of SEN with EHCPs**

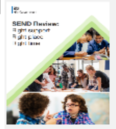

2022: SEND review: right support, right place, right time

Green paper proposing the establishment of "a new national SEND and alternative provision system setting nationally consistent standards for how needs are identified and met at every stage of a child's journey across education, health and care"

EHCPs: education, health and care plans; SEN: special educational needs; SEND: special educational needs or disability;

Figure 1 – Illustration of conceptual framework for exploring equity and impact of SEN interventions (in grey we mark factors not captured in ECHILD database)

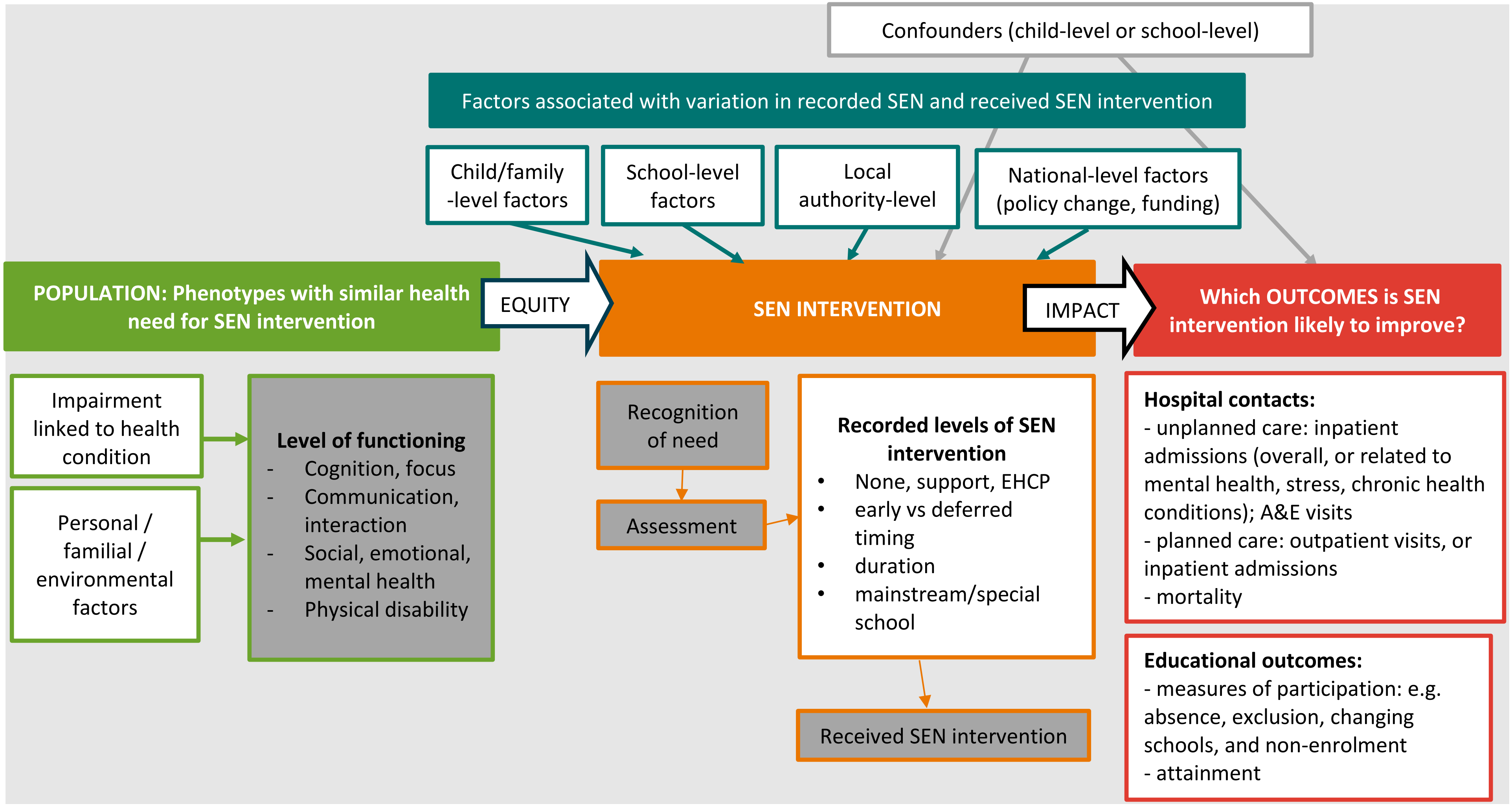

A&E: accident and emergency department; ECHILD: Education and Child Health Insights from Linked Data; EHCP: education, health and care plan; SEN: special educational needs;

Figure 3 – Coverage of ECHILD datasets, by academic year with refreshes

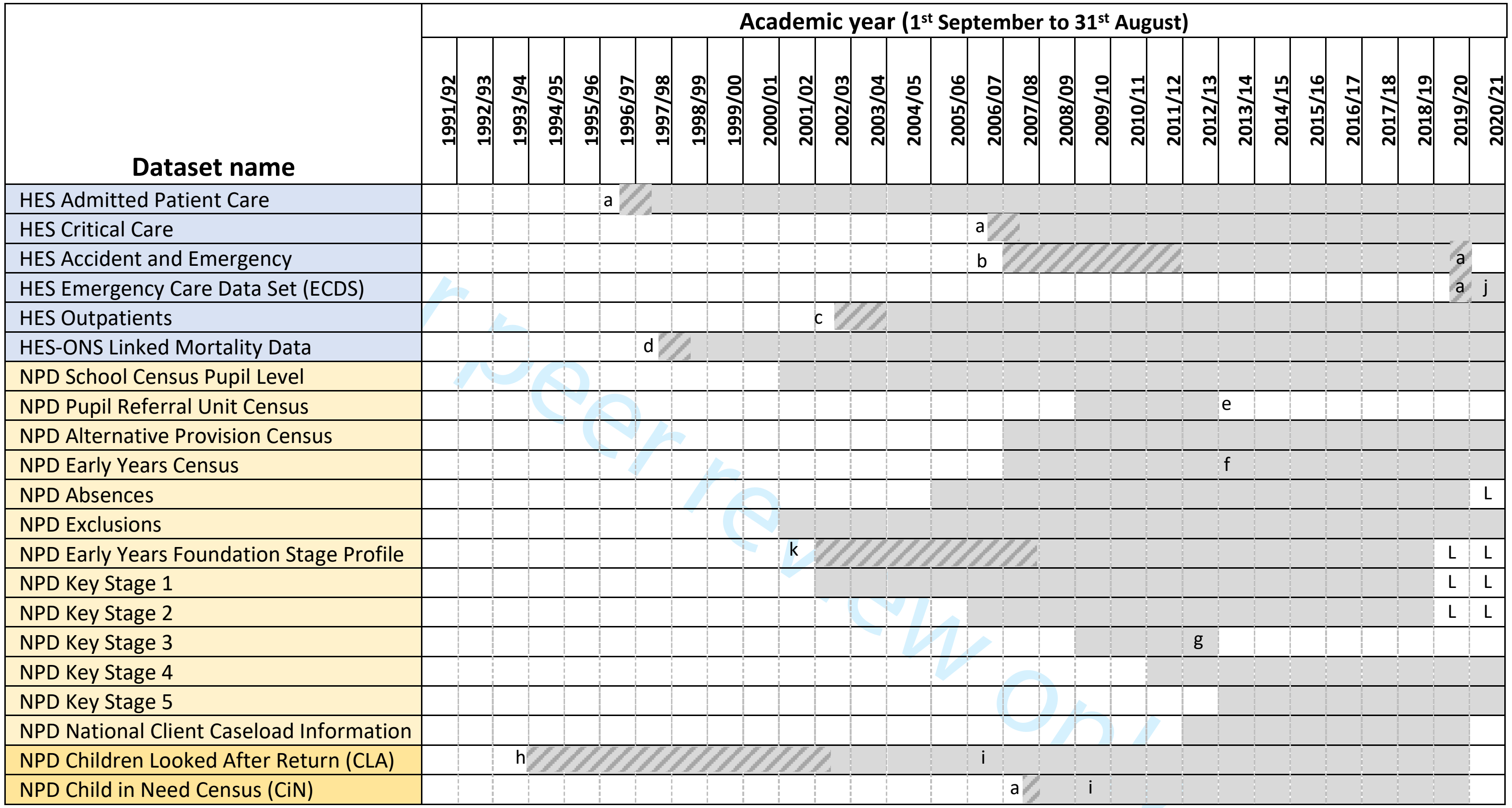

CiN: Child in Need Census, CLA: Children Looked After Return, HES: ECHILD: Education and Health Insights from Linked Data, Hospital Episode Statistics, NPD: National Pupil Database, ONS: Office for National Statistics

a: Partial coverage of an academic year as NPD social care data and HES data is collated by financial year (1st April to 31st March). b: Partial coverage as HES Accident and Emergency data was experimental and did not have full national coverage. c: Partial coverage as HES outpatient data was experimental and did not have full national coverage. d: Partial coverage of an academic year as ONS mortality data was first linked to HES in January 1998. e: The Pupil Referral Unit Census was subsumed in the School Census Pupil Level from 2013/14. f: The Early Years Census included 3- and 4-year olds between 2007/08 and 2012/13. From 2013/14, it includes 2-4 year olds. g: Key Stage 3 assessments ceased after 2012/13. h: Partial coverage of population as between 1st April 1998 and 31st March 2003, Children Looked After Return data was only collected for a one-third sample (i.e. children with a day of birth divisible by 3). i: Linkage between education and social care modules of NPD began in April 2006 for CLA and April 2009 for CIN. j: not currently linkable. k: Partial coverage as between the 2002/3 and 2005/6 academic years, data only on a 10% sample of children. L: not collected to help reduce the burden on educational and care settings during the coronavirus (COVID-19) pandemic.

Appendix: HOPE study glossary

Confounding: The bias caused by shared causes of exposure and outcome.[1]

Confounder: A variable that can be used to adjust for confounding.[1]

Disability: under the Equality Act 2010 a disability is "a physical or mental impairment which has a long-term and substantial adverse effect on their ability to carry out normal day-to-day activities". Not all children with SEN have a disability, and not all disabled children have SEN, but there is significant overlap.[2,3]

Education, health and care plan, EHCP: more intense provision arranged by local authorities, involving a legal document setting special measures provided by local authorities to meet a child's needs across education, health and social care.[2]

Health phenotypes: health conditions which can be indicated in administrative health records using diagnostic data (such as recorded diagnoses or procedures). In HOPE study we focus on health conditions associated with higher need for additional support for SEN than for their peers

Recorded SEN: information on children with SEN recorded by schools in termly school censuses. Recorded SEN indicates identification of a child's primary need using fixed categories (see Box 1) and level of appropriate intervention (either SEN support or EHCP).[2] However, there is no centrally collated data on when and what type of intervention was received.

Reasonable adjustments: schools have a duty to support pupils with medical conditions and make reasonable adjustments for children with disabilities, including the provision of auxiliary aids and services for disabled children. Not all children with disabilities have SEN, therefore reasonable adjustments are not considered SEN intervention.[2]

Special educational needs, SEN: a child has SEN if they have a learning difficulty or disability which calls for special educational provision (SEP) to be made for him or her.[2] Disability included in definition of SEN is a disability that prevents the child from using facilities generally provided for their peers in mainstream settings.[2]

SEN support: less intensive support arranged and funded by the school and provided almost entirely in mainstream schools

SEN intervention, or special educational provision, that is "provision different from or additional to that normally available to pupils of the same age"[2]

References

1. Hernan MA, Robins JM. *Causal Inference: What If*. Boca Raton: Chapman & Hall/CRC.; 2020.

2. Department for Education. Special educational needs and disability code of practice: 0 to 25 years. Published online 2015. doi:DFE-00205-2013

3. Cullen MA, Lindsay G, Hastings R, et al. Special Educational Needs in Mainstream Schools: Evidence Review.

For peer review only

BMJ Open

Evaluation of the variation in special educational needs provision and its impact on health and education: protocol for a mixed methods research programme in England

Journal:	*BMJ Open*
Manuscript ID	bmjopen-2023-072531.R1
Article Type:	Protocol
Date Submitted by the Author:	07-Jul-2023
Complete List of Authors:	Zylbersztejn, Ania; UCL Great Ormond Street Institute of Child Health Population Policy and Practice<br>Lewis, Kate; UCL Great Ormond Street Institute of Child Health Population Policy and Practice<br>Nguyen, Vincent; UCL Great Ormond Street Institute of Child Health Population Policy and Practice<br>Matthews, Jacob; University of Cambridge, Department of Psychiatry<br>Winterburn, Isaac; University of Cambridge, Department of Psychiatry<br>Karwatowska, Lucy; UCL Great Ormond Street Institute of Child Health Population Policy and Practice<br>Barnes, Sarah; University of Cambridge, Department of Psychiatry<br>Lilliman, Matthew; UCL Great Ormond Street Institute of Child Health Population Policy and Practice<br>Saxton, Jennifer; University of Cambridge, Department of Psychiatry<br>Stone, Antony; UCL Great Ormond Street Institute of Child Health Population Policy and Practice<br>Boddy, Kate; University of Exeter Medical School, Department of Health and Community Sciences<br>Downs, Johnny; King's College London, Institute of Psychiatry, Psychology and Neuroscience; South London and Maudsley NHS Foundation Trust<br>Logan, Stuart; University of Exeter Medical School, The Peninsula Childhood Disability Research Unit<br>Rahi, Jugnoo; UCL Great Ormond Street Institute of Child Health Population Policy and Practice; UCL Institute of Ophthalmology<br>Black-Hawkins, Kristine; University of Cambridge, Faculty of Education<br>Dearden, Lorraine; UCL Social Research Institute<br>Ford, Tamsin; University of Cambridge, Psychiatry<br>Harron, Katie; UCL Great Ormond Street Institute of Child Health Population Policy and Practice<br>De Stavola, Bianca ; UCL Great Ormond Street Institute of Child Health Population Policy and Practice<br>Gilbert, Ruth; UCL Great Ormond Street Institute of Child Health Population Policy and Practice
**Primary Subject Heading**:	Epidemiology
Secondary Subject Heading:	Public health, Qualitative research, Research methods, Paediatrics

For peer review only - http://bmjopen.bmj.com/site/about/guidelines.xhtml

Keywords:	EPIDEMIOLOGY, Health Equity, PUBLIC HEALTH, QUALITATIVE RESEARCH, STATISTICS & RESEARCH METHODS, Health informatics < BIOTECHNOLOGY & BIOINFORMATICS

SCHOLARONE™
Manuscripts

Evaluation of the variation in special educational needs provision and its impact on health and education: protocol for a mixed methods research programme in England

Ania Zylbersztejn[1], Kate Lewis[1], Vincent Nguyen[1], Jacob Matthews[2], Isaac Winterburn[2], Lucy Karwatowska[1], Sarah Barnes[2], Matthew Lilliman[1], Jennifer Saxton[2], Antony Stone[1], Kate Boddy[3], Johnny Downs[4,5], Stuart Logan[7], Jugnoo Rahi[1,8,9,10,11], Kristine Black-Hawkins[12], Lorraine Dearden[13], Tamsin Ford[2], Katie Harron[1], Bianca De Stavola[1], Ruth Gilbert[1]

[1]Population, Policy and Practice Research and Teaching Department, UCL Great Ormond Street Institute of Child Health, London, UK

[2]Department of Psychiatry, University of Cambridge, Cambridge, UK

[3]Department of Health and Community Sciences, University of Exeter Medical School

[4]Institute of Psychiatry, Psychology and Neuroscience, Kings College London, London, UK

[5]South London and Maudsley NHS Foundation Trust, London, UK

[7]The Peninsula Childhood Disability Research Unit, University of Exeter Medical School, Exeter, UK

[8]Great Ormond Street Hospital NHS Foundation Trust London, UK

[9]UCL Institute of Ophthalmology, London, UK

[10]NIHR Moorfields Biomedical Research Centre London, UK

[11]Ulverscroft Vision Research Group UCL London, UK

[12]Faculty of Education, University of Cambridge, Cambridge, UK

[13]UCL Social Research Institute, London, UK

Corresponding author: Dr Ania Zylbersztejn,

Population, Policy and Practice Research and Teaching Department, UCL Great Ormond Street Institute of Child Health, 30 Guilford Street, WC1N 1EH, London, UK

ania.zylbersztejn@ucl.ac.uk

Word count: 4500/4000

For peer review only

For peer review only - http://bmjopen.bmj.com/site/about/guidelines.xhtml

Abstract

Introduction: One-third of all children in England are recorded as having special educational needs (SEN) provision during their school career. The proportion of children with SEN provision varies between schools and demographic groups, which may reflect variation in need, inequitable provision or a combination of both. There is scant evidence on whether SEN provision improves health and education outcomes.

Methods: In this mixed methods study, we will analyse linked administrative data from the Education and Health Insights from Linked Data – ECHILD database – including data from all state schools, and contacts with NHS hospitals in England, to explore the impact of SEN provision on health and education outcomes. We will define a range of "health phenotypes", that is health conditions, expected to need SEN provision. We will describe how SEN provision recorded in education data (used as a proxy for receiving SEN provision) and health and educational outcomes vary for different health phenotypes. We will apply a range of causal inference methods to account for confounding factors and possible selection bias to draw conclusions about the impact of SEN provision on these outcomes, also considering timing, duration and level of provision. We will use mixed methods (surveys, interviews and focus groups with service users and providers, and document analyses) to understand variation in policies and in identification, assessment and provision for SEN, and how these processes are experienced by families. We will triangulate results to generate evidence to inform policy.

Ethics and dissemination: Research ethics committees have approved the use of the ECHILD database and, separately, the survey, interviews and focus groups of young people, parents and service providers. We are working with these stakeholders to help us interpret, frame and communicate our findings to policy makers, health and education services and families in order to promote translation of our findings into practice.

For peer review only

Strengths and limitations of this study

Strengths:

- We use the ECHILD database, which comprises longitudinal histories of all hospital contacts funded by the National Health Service (NHS) and state-funded schooling, to compare health and education outcomes in children with similar health phenotypes according to whether or when they had a record of SEN provision. We apply different methods and triangulate evidence from qualitative and quantitative analyses to strengthen the robustness of our findings.
- The population size (14.7 million children) and geographic coverage of the ECHILD data allow exploration of outcomes for different health phenotypes over time, by geographic area and sociodemographic characteristics of children in England.

Limitations:

- We can only measure health phenotypes as indicated in hospital admission records, meaning that we likely undercount children with less severe disease (not requiring hospitalisation). Severity of health phenotypes, which may impact on the child's function and need for SEN provision, can only be indicated using proxy measures such as co-existing chronic conditions, the frequency and type of hospital contacts and/or attainment scores.
- The ECHILD data does not capture NHS healthcare outside acute hospital settings, or education support at home or in the non-state funded sector.
- A record of SEN provision in school data does not evidence what (if any) SEN provision was received or whether it was appropriate, as these details are not recorded by schools.

For peer review only

For peer review only - http://bmjopen.bmj.com/site/about/guidelines.xhtml

Introduction

National policies in the UK and in many high-income countries require schools to make adaptations to meet the needs of children who have health, learning or behavioural problems, that impact their ability to learn; these children are referred to collectively as having special educational needs (SEN). In England, children with SEN "*have a significantly greater difficulty in learning than the majority of others of the same age, or have a disability which prevents them from making use of facilities generally provided by mainstream schools*" (see the study glossary in the appendix for details).[1]

There are four broad areas of SEN that state-funded schools are required to support: communication and social interactions, cognition and learning, social, emotional and mental health and sensory or physical disabilities (see Box 1). Interventions and adjustments in schools for children with SEN, referred to as *SEN provision*, are intended to improve inclusion and participation in education and support children's health and wellbeing.[1] Approximately 7% of children in England attend private schools each year,[2] which do not have the same legal obligations regarding SEN identification and provision.

Box 1 – Broad areas of special educational needs that schools should plan for, with sub-categories set out by the Special Educational Needs and Disability code of practice in England[1]

Communication and social interactions
- Speech, language and communication needs
- Autistic Spectrum Disorders

Cognition and learning:
- Moderate learning difficulties
- Severe learning difficulties
- Profound and multiple learning difficulties
- Specific learning difficulties (that is learning difficulties affecting one or more specific aspects of learning, such as dyslexia, dyscalculia or dyspraxia)

Social, emotional and mental health
- Examples could be attention-deficit/hyperactivity disorder, behavioural difficulties, anxiety, depression, eating disorders

Sensory and/or physical disabilities:
- Vision impairment
- Hearing impairment
- Multi-sensory impairment
- Physical disability requiring additional ongoing support and/or equipment to access all the opportunities available to their peers

Since 2015, approximately one in six children in England are recorded by schools as receiving any SEN provision each year (see Figure 1),[3] and one-third of all children have a record of any SEN provision at least once during their time in education.[4,5] There are two categories of SEN provision offered in England, *SEN support* and *Education, Health and Care Plans (EHCPs)*. These categories were introduced following Government education reforms in 2014/15, replacing the older categories of *School Action/School Action Plus* (together referred to as *SEN without Statement*) and *Statements of SEN*, respectively (see study glossary for details).

For peer review only - http://bmjopen.bmj.com/site/about/guidelines.xhtml

The majority of pupils with any recorded SEN provision receive *SEN support* (Figure 1). SEN support is arranged and funded by the schools and can include short-term interventions such as speech or language therapy or extra support for reading. The first assessment for SEN support is usually carried out by the school's teachers, Special Educational Needs Coordinator (SENCo), or after class teachers, who seek to identify children making less than expected educational progress or with additional social needs relative to their peers. In 2018/19 (the last academic year before the COVID-19 pandemic), 11.9% of pupils had SEN support recorded, with the vast majority provided in mainstream schools.[6]

A smaller proportion of children receive an EHCP, which involves additional and more intensive provision arranged and partly funded by local authorities for children whose needs cannot be fully met by SEN support.[3,7] Support may range from extra help by a part-time teaching assistant to full-time care by multiple staff in a special school.[1] An assessment for an EHCP can be requested by parents, schools or health or social care professionals. The assessment is carried out by the local authority, who are required to fill in a legal document setting out the special measures to be provided to meet the child's needs across education, health and social care.[3,7] In 2018/19, 3.1% of pupils had a record of an EHCP, half of whom were enrolled in a special school (1.6% of all children).[6]

Schools record information on children identified as needing SEN provision (SEN support or EHCP) in school censuses returned to the Department for Education (DfE). We refer to this recording as *SEN provision* throughout the protocol, although we acknowledge that an indication of "SEN provision" in educational records does not evidence that SEN provision is actually received or whether it is appropriate, as these data are not recorded by schools.[2]

SEN provision across England is widely regarded as inequitable.[3,5,8,9] The proportion of pupils with SEN support ranged from 7.3% to 17.1% and the proportion with EHCPs from 0.8% to 5.0% across local authorities in 2018/19.[6] Allocation of SEN provision is associated with a variety of factors. According to a recent report, a key factor determining SEN provision is the school, particularly school's previous rates of SEN provision, academy status and previous school inspection outcomes.[5] Other factors include the proportion of academised primary schools and rates of pupils eligible for free school meals at local authority level-and pupil-level factors such as attainment at school entry (age 5), ethnic group, child's first language and contacts with social care.[5,8,9] The annual proportion of children with recorded SEN provision has also declined over time, from 20% in 2010 to 14% in 2016. This change seems partly related to the Children and Families Act in 2014 and Special Educational Needs and Disability Code of Practice implemented in 2015, and to reduced funding to local authorities from 2010 (Figure 1).[3,10]

Compared to their peers, children with SEN provision have higher rates of chronic physical and mental health conditions and hospitalisations, and have lower self-reported wellbeing.[11–13] Recent evidence reviews report that classroom-based SEN interventions can improve children's social and emotional wellbeing and reduce challenging behaviour and contribute to better mental health outcomes.[14,15] For children with attention deficit hyperactivity disorder (ADHD), systematic reviews of randomised, or quasi randomised controlled trials (RCTs) of interventions similar to SEN provision found improvements in behaviour.[11,16–18] However, there is a lack of RCTs or representative observational comparative studies of the impact of SEN provision on health outcomes for a range of health phenotypes.

Robust evidence that SEN provision improves educational outcomes for pupils with SEN is also scarce.[14] There is moderate evidence that SEN provision in primary schools improve literacy difficulties, socio-emotional development and language and communication.[15] A recent evidence review found a weak but positive impact of inclusive education involving additional support for those with additional learning needs on academic outcomes among pupils without SEN provision.[14] Evidence from population-based observational studies suggests that SEN provision is associated with

For peer review only - http://bmjopen.bmj.com/site/about/guidelines.xhtml

fewer absences and exclusions among children and young people with neurodisability or mental health conditions.[19]

Given the large proportion of children with SEN provision indicated in school records, the high costs of SEN provision, and static school funding per pupil since 2010,[10] evidence is needed to guide effective intervention to groups of children who are most likely to benefit. The HOPE research programme aims to address these gaps in evidence by assessing two central research questions: i) how has SEN provision changed over time, and how does it vary between geographical areas and by child characteristics; and ii) what is the impact of SEN provision on health and education outcomes? We will address these questions for a range of health conditions associated with increased need for SEN provision, which we refer to as *health phenotypes*.

We hypothesise that the impact of SEN provision on health and education outcomes will vary for children with different health phenotypes. For example, children with health phenotypes linked to delayed communication and conduct problems may experience improved communication, less frustration and distress and fewer unplanned hospital contacts as a result.[20] Children with complex health needs who require an EHCP may benefit from additional physio- or occupational therapy, which reduces risks of health complications and thereby, presentations to hospital. Children from more socioeconomically deprived backgrounds may benefit more from SEN provision than less deprived children, who are likely to have more resources available at home. Hence, the impact of SEN provision may vary according to health phenotype and social context. Evidence based on similar methods of evaluation across a range of health phenotypes is needed to identify what works for whom and in which contexts.

This protocol sets out a mixed methods research programme, with multiple component studies (conceptual framework and proposed research questions are illustrated in Figure 2). The programme integrates quantitative analyses of the Education and Child Health Insights from Linked Data (ECHILD) database (see data resources) with mixed quantitative and qualitative methods to understand variation in identification, assessment and provision for SEN, and how these processes are experienced by families. We will explore which causal inference methods can be used to provide valid evidence of the impact of SEN provision on health and education outcomes, and in which contexts. Each component study in the programme will be reported using relevant reporting guidelines from the EQUATOR Network (https://www.equator-network.org/, e.g.: analyses using ECHILD will be reported using RECORD guidelines for studies using linked administrative data).[21] The programme started in August 2021 and is expected to end in March 2025. We consulted children and young people who chose to name this research programme HOPE: Health Outcomes of young People in Education (HOPE).

Methods and analysis

Data sources

ECHILD database

The ECHILD database links routinely collected administrative data on health and education in England. Currently ECHILD includes all children and young people aged 0–24 years in England who were born between 1 September 1995 and 31 August 2021 (approximately 14.7 million individuals).[22] Health and education datasets were linked by NHS England using a multi-step deterministic linkage algorithm, described in detail elsewhere. Linkage rates were high and increased over time (99% of pupils born in 2004/05 were linked to a hospital record).[23]

Health data consists of Hospital Episode Statistics (HES), a national database that includes dated information on all National Health Service (NHS) acute hospital care and mortality data (see Figure 3 for details of data coverage by academic year).[24] Nearly all children born in England are born in NHS

For peer review only - http://bmjopen.bmj.com/site/about/guidelines.xhtml

hospitals (97%) and can be followed from their birth admission through all subsequent hospital contacts.[25,26]

Education records are collated in the National Pupil Database (NPD) and include information on children's registrations in schools, attainment scores at ages 5, 7 11, 16 and 18 (see Study Glossary in the appendix for details), and number of half-day absences and exclusions in each 13-week term. SEN provision is recorded each term (annually prior to 2005/6) for all children in state-funded education (93% of all children) from the academic year starting in September 2001 onwards (see Figure 3).[2] Education data captures the category of recorded SEN provision (SEN support or EHCP) and main reason for SEN provision. Most recent categories reflect language or communication, moderate or severe learning disability, autism, sensory impairment, physical disability, or social, emotional or mental health needs (see Box 1 for details). We are in the process of enhancing ECHILD with school characteristics, such as type of school (e.g.: mainstream or special) or teacher-pupil ratio, from publicly available data.[27]

National online survey

We conducted three online surveys aimed at (1) children and young people, (2) parents/carers and (3) service providers (health, education and local authority professionals) to document variation in local experiences of identification and assessment of need, and provision of SEN interventions. The surveys were developed through a scoping review to identify previous questionnaires and co-designed with stakeholder groups of young people, parents / carers and professionals working in education or health with children who have SEN. Data were collected using REDCap. The surveys were disseminated via social media (Twitter, Instagram, and Facebook), and through professional networks (GOV.UK Notify service, Parent and Carer forums, and stakeholder group contacts). These networks were used to maximise the recruitment of all three groups from each of the nine regions in England. We received ethics approval for this study (see Ethics section for details).

In total 1,714 participants took part from across England including: 77 young people aged 11-27 years, 772 parents and carers, and 865 service providers (those working in/closely with education settings, the health services, local authorities). Short summaries of the key findings from initial analyses are published on the study website (https://dev.psychiatry.cam.ac.uk/hope-study-health-outcomes-for-young-people-throughout-education/) and more detailed papers are in preparation. These data will inform further qualitative components of the study.

Study population: health phenotypes

We will use clusters of coded information in the ECHILD database to define *health phenotypes* that represent health conditions associated with learning impairment or need for additional educational support. Initially, we will focus on primary school aged children (enrolled in school in year 1 aged 5/6 and followed to year 6 aged 10/11), who were born in England and had a birth admission recorded in HES data from school year 2002/03 onwards. This approach ensures follow up from the same point (birth) for all children and captures information about risk factors at birth (such as gestational age, birth weight).[25,26]

Between 1st September 2009 and 31st August 2017 there were 5,004,354 children entering primary school (recorded in school census in Year 1, aged 5-6 years old), of whom 94% had a linked HES record and 80% had a linked birth record (the linkage rate increased over time, see Appendix Table 1). As these numbers are large, we can focus on specific or even uncommon phenotypes.

We propose three sets of health phenotypes which capture populations with different levels of need for SEN provision:

For peer review only - http://bmjopen.bmj.com/site/about/guidelines.xhtml

a) Neurodisabilities and other high-risk conditions

The first group comprises neurologic conditions or complex systemic health problems reported to be associated with learning impairment or behaviours that require SEN provision. These include neurodevelopmental conditions such as autism or learning disabilities,[28,29] cerebral palsy,[30] or epilepsy.[31] The list of health phenotypes will be derived from an overview of systematic reviews and population-based observational studies (see appendix for overview of search terms) and discussions with clinical experts and service providers. We will then develop coding algorithms for these health phenotypes based on combinations of diagnostic and procedure codes, where possible from previously validated code lists. We will compare the cumulative incidence and mortality rates by age for each health phenotype with external population studies (for example, from national surveys and disease registries) to assess consistency of recorded diagnoses in hospital records.

Our preliminary findings for approximately 50 high-risk health phenotypes, show that children with these conditions recorded in hospital records before the age of 5 account for approximately 5% of all children starting primary school in 2008/9-2018/19, 10% of children with any recorded SEN provision during primary school and 30% of those with an EHCP. Some of the included conditions (such as autism or learning disability) are likely to be under-reported in hospital records.[32]

b) Major congenital anomalies

The second group includes children with major congenital anomalies (MCAs), as children with MCAs are likely to require support from specialist services and have a diagnosis recorded in hospital admissions records, creating a reliable phenotype with a variation in need for SEN provision.[33] We will draw from a code list of ICD-10 diagnoses developed by EUROCAT – a European Congenital Anomaly Registry (https://eu-rd-platform.jrc.ec.europa.eu/eurocat),[34,35] which groups MCAs into 12 body system groups and includes 25 specific conditions.

Our preliminary findings suggest that children with MCAs recorded in first year of life account for 3.5% of the primary school population in 2008/9-2018/19, 5.5% of children with any recorded SEN provision during primary school, and 13.6% of those receiving EHCPs in mainstream school or attending specialist provision.

c) Whole population phenotype: gestational age

Finally, we will use week of gestational age at birth to assess the gradient in impact of SEN provision across the whole population of children, stratified according to different levels of underlying need. This approach is supported by the finding that each week of birth before 40 weeks of gestation is associated with reduced school attainment scores and an increased risk of SEN intervention.[12,36,37] Approximately 4.5% of children in primary school in 2008/9-2018/19 were born preterm (at <37 weeks' gestation), accounting for 6.0% of children with any recorded SEN provision during primary school, and 8.4% of those receiving EHCPs.

Study measures

Health and education outcomes

We focus on outcomes that can be measured in hospital and education data: planned and unplanned hospital contacts, school attainment (as proxy measure for cognitive function), and rates of school absences (see Box 2 for study measure definitions). Outcomes that are evaluated will depend on the specific phenotypes being assessed.

For peer review only - http://bmjopen.bmj.com/site/about/guidelines.xhtml

The COVID-19 pandemic had a significant impact on the wellbeing of young people.[38] Lockdowns in England affected children's access to school and the frequency of hospital contacts captured in ECHILD data. Planned and unplanned admissions, and outpatient appointments reduced substantially during the COVID-19 pandemic, with the largest reductions in children with indicators of vulnerability (such as preterm birth, a chronic condition, recorded SEN or social care record).[13,39] School attainment measures were not collected during the pandemic to help reduce the burden on educational and care settings. In the HOPE programme, we will therefore limit our analyses to outcomes recorded before the start of COVID-19 pandemic, until more data after COVID-19 become available to describe changes in health and education outcomes, including participation in schooling, in children with health phenotypes expected to need SEN intervention and comparator cohorts, before and after the onset of COVID-19.

Recorded SEN provision

We will categorise SEN provision in a descending hierarchy for a specified time period (e.g., during school Year 1 or across all of primary school; details shown in Box 2), separating any enrolment in a special school or alternative provision (where the vast majority of children have recorded SEN provision), an EHCP in mainstream school, SEN support in mainstream school, and no recorded SEN provision. These categories were selected due to differences in the presumed type of provision, in the criteria for provision (e.g.: formal assessment is required for EHCP but not for SEN support) and substantial differences in associated costs.[3]

For peer review only

Box 2: key study measures derived from ECHILD database

Accident & Emergency (A&E) department contact rate: defined as the number of days with at least one A&E contact, divided by person-time at risk during the study period (e.g.: time from start of Year 1 until the end of Year 6 or death).

Unplanned/planned admission rate: defined as the number of unplanned or planned hospital admissions in NHS-funded hospitals in England, divided by person-time at risk during the study period. Admissions will be classified as planned/unplanned according to the admission method recorded in the first episode of care. Consecutive admissions with re-admission on the same day as discharge (for example, hospital transfers) will be treated as part of the same admission. Time spent in hospital during an admission will be taken out of the person-time at risk as once a child is in hospital they cannot be at risk of a new admission.

Outpatient department (OPD) appointments and attendances: defined as the number of days with at least one OPD contact, divided by person-time at risk during the study period (e.g.: time from start of Year 1 until the end of Year 6 or death).

Absence rate: schools are required to take attendance registers twice a day, for morning and afternoon sessions. Absence rate will be defined in line with definition used by Department for Education as the total number of absent sessions (including authorised and unauthorised absences) divided by the total number of possible sessions during the study period**.**

Standardised attainment measures: We will derive standardised attainment measures using recorded scores from national tests in reading, writing and maths at the end of Year 2 (aged 7, Key Stage 1) and at the end of Year 6 (aged 11, Key Stage 2). Standardised test scores will be calculated using mean and standard deviation of the test scores of all pupils in a given academic year. We will present the proportion of children not assessed (i.e.: did not have an assessment record) and average score for those assessed by study population.

School readiness indicators: we will use scores from teacher assessments of children's development across multiple areas of learning, carried out in the final term before Year 1 (Early Year Foundation Stage Profile, EYFSP). Standardised EYFSP scores will be calculated using mean and standard deviation of the EYFSP scores of all pupils in a given academic year. We will present the percentage of children who were not assessed (i.e.: did not have an assessment record), the proportion of children not reaching Good Level of Development (GLD, defined by Department for Education using a subset of EYFPS scores) and average scores for those assessed by study population.

SEN provision: we will look at 4 categories (which may be merged for some analyses) in the following descending hierarchy for a specified time period (e.g.: Year 1):

1) No SEN provision,
2) SEN support (including 'School Action', 'School Action Plus' or 'SEN support') in mainstream school,
3) EHCP (including 'Statement of SEN' or 'EHCP') in mainstream school,
4) Enrolment in specialist provision (including special school or alternative provision such as pupil referral units).

Analysis plan

Describing health and education outcomes

For each set of phenotypes, we will estimate rates of planned and unplanned hospital contacts and educational outcomes during primary school. We will use appropriate generalised linear models for

For peer review only - http://bmjopen.bmj.com/site/about/guidelines.xhtml

each outcome (e.g.: Poisson regression for rates, logistic regression for binary outcomes). Findings from these analyses will inform the design of causal questions.

Variation in recorded SEN provision

We will examine how proportions of children with recorded SEN provision changed over time for children with different health phenotypes (e.g.: MCAs), and the percentage in their variation that is explained by factors at the individual-, school- and local authority-level using appropriate multi-level regression models. Data analysis plans for specific studies examining time trends and geographic variation will be pre-registered on NIHR Open Research platform (https://openresearch.nihr.ac.uk/).

Understanding lived experiences of SEN provision

The ECHILD database contains termly records indicating provision for SEN, but no information about whether any provision was actually received, when, or its type or quality. We will apply mixed methods to understand geographic variation in the underlying processes of identification, assessment and provision, and how these processes are experienced by families.

First, we will review publicly available documents on the support available for local children with SEN, referred to as "local offer", against 51 criteria outlined within the SEND Code of Practice in order to assess the extent that Local Authorities in England are providing clear, comprehensive, accessible and up-to-date information about available SEN provision and how to access it. Second, we will examine reports from all of the local area SEND inspections published by the Office for Standards in Education, Children's Services and Skills (OFSTED) over the past seven years to examine how effectively local authorities fulfil responsibilities for children and young people with SEN. By collating these documents and assessing their commonalities and differences, we aim to gain an understanding of good and bad practice in SEN provision.

Third, based on results of our national survey of experiences of SEN provision, we will undertake two qualitative studies. The first will use a time-line approach with young people (20-25) and parents (20-25) to gather their experience about the identification, assessment and provision of SEN. Secondly, we will complete nine focus groups with SEN professionals (those working in/closely with education settings, health services, and the local authority) to explore concepts of best practice in SEN and barriers to implementation. There will be three focus groups that will focus on each stage in the process (identification, assessment and provision). We will train and support one parent from our stakeholder group to help facilitate each theme, working with three parents in total. We will recruit participants for both studies from national online survey respondents who have consented to be contacted about further qualitative analyses as part of the survey. Data from each set of stakeholder focus groups and for parents / carers and young people will be analysed separately using a Framework Analysis approach and then compared to look for consensus and differences in views and experiences across informants. We received ethics approval for this study (see Ethics section for details).

Impact of SEN provision on outcomes

We will use a range of biostatistical and econometric methods to explore the impact of SEN provision on health and educational outcomes across selected health phenotypes, and triangulate findings from analyses using different methods. First, we will examine the impact of recorded SEN provision at a given point in time, for example in the first year of compulsory education (Year 1). Second, we will assess the impact of the duration of SEN provision, appropriately controlling for likely time-varying factors that may be affected by SEN provision and additionally influence future SEN provision. Specific study protocols will be registered NIHR Open Research.

For peer review only - http://bmjopen.bmj.com/site/about/guidelines.xhtml

For all causal analyses, we will use the target trial emulation (TTE) framework to guide the creation of study cohorts that correspond to the specific phenotypes and exposure levels (categories of SEN provision) of interest.[40,41] TTE consists of first designing an ideal pragmatic trial that would address the question of interest, and then emulating it as closely as possible using observational data. The advantage of this approach is the avoidance of biases in the design stage, e.g. immortal time bias and prevalent-case bias, that have affected real-world-data studies in the past.[40] Directed Acyclic Graphs (DAGs) will be used to draw our assumptions about the causal structures influencing what we are studying and to identify relevant confounding variables.[42] An outline of the components of our causal investigations is given in Table 1 where these steps are illustrated using the exemplar of one MCA phenotypes: children born with cleft lip and palate. The study protocol for this study has been registered as a preprint.[43]

As ECHILD comprises observational data, a major challenge for each of our causal investigations will be how to address the bias introduced by confounding by indication, as well as possible selection bias due to incomplete linkage across health and education databases. We will contrast estimates of the impact of SEN provision on health and educational outcomes using a variety of complementary methods that rely on measuring all of the confounders, such as regression adjustment, g-computation, inverse probability weighting of marginal structural models (with different approaches to modelling the propensity score), or econometric methods that try to deal with unmeasured confounding by exploiting natural experiments (such as differences-in-differences or interrupted time series), or instrumental variables. The latter set of approaches will be possible if we can identify indicators of exogenous drivers (i.e.: circumstances that create a natural experiment or instruments) that systematically change SEN provision while not changing factors that could affect the outcome of interest apart from through SEN provision. This could be due to changes in policy or funding, or policy variation between areas or schools.

Potential confounders will vary between settings. We will use the rich longitudinal records from health and education data before the start of any intervention period (e.g.: Year 1) to account for the severity of health conditions or functional impairment. For example, school attainment tests at age 5 (see Box 2) could provide proxy measures of baseline cognitive ability.[5] Baseline health data, for example, indicating other chronic health conditions or frequency of hospital contacts can be used to distinguish children who are expected to have more functional impairment and increased need for SEN provision within the same phenotype group. For phenotypes involving congenital anomalies, we will distinguish those with a more extensive malformation (e.g.: cleft lip and palate vs cleft lip alone), or multiple malformations (e.g.: Down syndrome with or without congenital heart malformation), to create sub-phenotypes with similar need for SEN provision.

For peer review only - http://bmjopen.bmj.com/site/about/guidelines.xhtml

Table 1 – Roadmap for causal investigations in HOPE, with an exemplar of cleft lip and palate

	**Steps in the design of the study:**	**Exemplar: *Children with cleft lip and palate (CLP) abnormalities***
**1.**	Articulate the scientific question and specify the background knowledge:	*Does special educational needs (SEN) provision improve the health and educational outcomes of children with CLP?*
	a) define the population of interest	Children with CLP identified in HES before age 5, born between 2003 and 2012, who started compulsory education between 2008 and 2018, with linked HES and NPD data
	b) specify the outcomes	• Number of days in contact with an accident and emergency department by Year 6<br>• Number of unplanned school absences by Year 6
	c) specify the intervention ("exposure")	• SEN support vs no recorded SEN provision<br>• SEN support vs EHCP
	d) draw assumptions regarding exposure, outcome and their common causes	*Draw DAG that includes unmeasured variables (as relevant) and identifies a minimum set of confounding variables*
	e) translate the causal question in terms of a contrast of means of potential outcomes*	*What are the benefits for the children who did receive SEN provision?*<br>→ Average treatment effect in the treated (ATT)<br><br>*Would other children with CLP benefit from SEN?*<br>→ Average treatment effect in the non-treated (ATNT)<br><br>*What would be the consequence of a new policy that increases the provision of SEN for all children with CLP? Or for those with more severe CLP?*<br>→ Interventional treatment effect (ITE)
**2.**	Can the question be addressed with the data at hand?	
	a) is the exposure well-defined and available in the data?	We have access to two categories of recorded SEN provision (SEN support and EHCP) and treat each of them to represent delivered support (covering a variety of different interventions)
	b) is the exposure suitable/available for everyone in the population of interest?	Some groups of children appear not to be eligible for an EHCP
	c) is there an issue of selection bias?	Missing values affect some of the confounding variables
**3.**	Causal contrasts	
	a) For which (sub-)population we wish to address the causal question?	Depending on the question:<br>• Children with CLP who have a record of any SEN provision;<br>• Children with CLP who do not have a record of any SEN provision;<br>• All children with CLP;<br>• Children with more severe CLP;
	b) On which scale?	For both outcomes we will examine:<br>– rate ratios

For peer review only - http://bmjopen.bmj.com/site/about/guidelines.xhtml

		– rate differences
**4.**	Estimation:	
	a) Which estimation approach would target the causal contrast we are interested in?	For the ATT and ATNT:<br>• G-computation;<br>• Inverse probability weighting (IPW) of marginal structural models (MSMs), with alternative approaches to specify the propensity score;<br>• Doubly robust methods;<br>• Difference-in-Differences;<br><br>For the ITE:<br>• G-computation<br>• IPW of MSMs, with alternative approaches to specify the propensity score<br>• Doubly robust methods
	b) Are the assumptions invoked by alternative estimation approaches defensible?	• Unmeasured confounding may be at play;<br>• Parametric models used may be misspecified but robust methods can be used;
**5.**	Interpretation	
	a) Are the results comparable? And why not if not?	If comparable, results are more robust to misspecification and unmeasured confounding
	b) Triangulating results and compare with external evidence	Current evidence on impact is limited

**Potential outcomes are the outcomes that would occur under intervention on the exposure ATNT= Average treatment effect in the non-treated, ATT=Average treatment effect in the treated, CLP= cleft lip and palate, DAG=Directed Acyclic Graph, EHCP=Education, Health and Care Plan, HES=Hospital Episode Statistics, IPW=Inverse Probability Weighting, ITE=Interventional treatment effect, MSM=Marginal Structural Models, NPD=National Pupil Database, SEN=Special Educational Needs*

Public involvement and engagement

The HOPE study was developed in response to consultations about the need for the ECHILD database with parents and charities supporting children with chronic health conditions and their families.[44,45] We have established three stakeholder groups: young people, parents / carers and professionals working in education or health with children who have SEN. We will collaborate with staff in schools to enable young people with additional learning needs or disabilities to contribute to these advisory group. In addition, we will present our planned analyses and preliminary results to parents/carers and young people through research advisory groups at UCL and University of Exeter. These consultations will contribute evidence to all four work packages and dissemination of the research. The HOPE Study Steering Committee includes two parents of children with disability and will adhere to NIHR requirements for payment for time and expenses of lay contributors.

Ethics

Existing research ethics approval has been granted for analyses of the ECHILD database for the purposes set out in the HOPE study (20/EE/0180). Data access is also controlled by agreements with NHS Digital and the DfE. The data contain no identifiers or sensitive dates and data can only be used within the Office for National Statistics Secure Research Environment by approved researchers, with strict statistical disclosure controls of all outputs of analyses (e.g. tables or figures). As these are

For peer review only - http://bmjopen.bmj.com/site/about/guidelines.xhtml

deidentified data, no consent is required for use. Details are published here in our privacy notice (https://www.ucl.ac.uk/child-health/research/population-policy-and-practice-research-and-teaching-department/cenb-clinical-4#).

Separate ethics approval has been approved for the mixed-methods research (national survey, interviews and focus groups) involving service users (young people and parents) and service providers (PRE:2021.058). Parents consented for their own involvement and also for their child if under the age of 16. Young people aged 16 or over consented and younger children were asked for assent to their participation using a similar process.

Dissemination

We will present preliminary findings to diverse audiences (academics, analysts at DfE and Department of Health and Social Care, and our stakeholder groups as well as other groups of service users and providers) through seminars, question and answer sessions, workshops and consultations during the study. We will incorporate feedback into final outputs, which will include peer reviewed journal articles, the final study report to funder, and short briefing reports and infographics for non-academics published on the study website.

We will publish our methods and code to enable others to reproduce and extend our analyses using ECHILD. ECHILD can be accessed by accredited researchers through application via the ECHILD team (www.ucl.ac.uk/child-health/echild) and the Research Accreditation Panel. Meta-data and code relating to the HOPE study will be signposted on the study website and made available in the ONS secure environment and in code repository (including on ECHILD GitHub page: https://github.com/UCL-ECHILD). We will hold workshops to promote wider use of findings from the HOPE study for causal analyses of education interventions on health. Examples from the HOPE study will be incorporated into short courses on causal methods and on how to use the ECHILD database.

The HOPE study aims to build the evidence base for fairer and more effective SEN provision and, by informing national and local policy and the public and changing practice, to improve health and education outcomes of children with SEN.

For peer review only - http://bmjopen.bmj.com/site/about/guidelines.xhtml

Figure captions

Figure 1 – Percentage of total children with recorded SEN provision per school year, January 2003 to 2022

Legend: EHCPs: education, health and care plans; SEN: special educational needs; SEND: special educational needs or disability;

Figure 2 – Conceptual framework bringing together research questions (RQ) to be addressed by component studies of the HOPE research programme

Legend: ECHILD: Education and Child Health Insights from Linked Data; EHCP: education, health and care plan; SEN: special educational needs;

Figure 3 – Coverage of ECHILD datasets, by academic year with refreshes

Legend:
CiN: Child in Need Census, CLA: Children Looked After Return, HES: ECHILD: Education and Health Insights from Linked Data, Hospital Episode Statistics, NPD: National Pupil Database, ONS: Office for National Statistics.

a Partial coverage of an academic year as NPD social care data and HES data is collated by financial year (1st April to 31st March). b Partial coverage as HES Accident and Emergency data was experimental and did not have full national coverage. c Partial coverage as HES outpatient data was experimental and did not have full national coverage. d: Partial coverage of an academic year as ONS mortality data was first linked to HES in January 1998. e: The Pupil Referral Unit Census was subsumed in the School Census Pupil Level from 2013/14. f: The Early Years Census included 3- and 4-year olds between 2007/08 and 2012/13. From 2013/14, it includes 2-4 year olds. g: not collected to help reduce the burden on educational and care settings during the coronavirus (COVID-19) pandemic. h: Partial coverage as between the 2002/3 and 2005/6 academic years, data only on a 10% sample of children. i: to be included, but not available yet. j: Key Stage 3 assessments ceased after 2012/13. k: data not provided with standard institutional identifiers in 2019/20 – 2020/21, as evaluation of individual institutional performance is not permitted.

For peer review only

Footnotes

Abbreviations

A&E: accident and emergency department

ADHD: attention deficit hyperactivity disorder

ASD: autistic spectrum disorders

ATNT: Average treatment effect in the non-treated

ATT: Average treatment effect in the treated

CLP: cleft lip and palate

DAG: Directed Acyclic Graph

DfE: Department for Education

ECHILD: Education and Child Health Insights from Linked Data

EHCP: education, health and care plan

HES: Hospital Episode Statistics

IPW: Inverse Probability Weighting

ITE: Interventional treatment effect

MCA: Major Congenital Anomaly

MSM: Marginal Structural Models

NHS: National Health Service

NPD: National Pupil Database

OFSTED: the Office for Standards in Education, Children's Services and Skills

RCT: randomised controlled trial

SEN: special educational needs

TTE: target trial emulation

Author contributions

RG is the principal investigator of the HOPE research programme and KH, TF, LD and BDS lead each of the four work packages, KBH, JD, SL, JR are co-investigators of the programme. AZ and RG produced the first draft of this manuscript, AZ, KL and ML created figures for the manuscript. AZ, KL, VN, AS, ML are responsible for preparing ECHILD data for analyses. JM, IW, SB, JS, KBH and TF are responsible for conceptualisation and implementation of mixed methods studies. BDS, LD, KL, VN, and LK are responsible for design and implementation of quantitative causal inference analyses using ECHILD database. AZ, KH, RG, KL are responsible for design and implementation of descriptive analyses using ECHILD database, with clinical input from JD, SL, JR on defining study populations. JS, JM, IW, KB, KBH, TF, RG, KH, AZ, VN were involved in public engagement activities to inform the study. All authors edited the manuscript. All authors have contributed to and are responsible for the final design of the study. All authors read and approved the final manuscript.

For peer review only - http://bmjopen.bmj.com/site/about/guidelines.xhtml

Acknowledgments

We would like to acknowledge the contribution of members of the HOPE study steering committee.

We are grateful to the Office for National Statistics (ONS) for providing the trusted research environment for the ECHILD Database. We gratefully acknowledge all children and families whose de-identified data are used in this research.

The ECHILD Database uses data from the Department for Education (DfE). The DfE does not accept responsibility for any inferences or conclusions derived by the authors. This work also uses data provided by patients and collected by the National Health Service as part of their care and support. Source data can also be accessed by researchers by applying to NHS Digital. This work was produced using statistical data accessed via the ONS Secure Research Service. The use of this data in this work does not imply the endorsement of the ONS in relation to the interpretation or analysis of the statistical data. This work uses research datasets which may not exactly reproduce National Statistics aggregates. This presentation has been cleared for publication by ONS (STATS17409, STATS15494).

Competing Interests

We declare no competing interests.

Funding

This study/project is funded by the National Institute for Health Research (NIHR) under its Programme Grants for Applied Research Programme (Grant Reference number NIHR202025). The views expressed are those of the author(s) and not necessarily those of the NIHR or the Department of Health and Social Care.

Patient and public involvement

Patients and/or the public were involved in the design, or conduct, or reporting, or dissemination plans of this research. Refer to the Methods section for further details.

For peer review only

For peer review only - http://bmjopen.bmj.com/site/about/guidelines.xhtml

References

1 Department for Education. Special educational needs and disability code of practice: 0 to 25 years. 2015. DOI:DFE-00205-2013.

2 Jay MA, Grath-Lone LM, Gilbert R. Data Resource: the National Pupil Database (NPD). *Int J Popul Data Sci* 2019; **4**. DOI:10.23889/ijpds.v4i1.1101.

3 The National Audit Office. Support for pupils with special educational needs and disabilities in England. 2019. https://www.nao.org.uk/wp-content/uploads/2019/09/Support-for-pupils-with-special-education-needs.pdf (accessed Jan 4, 2023).

4 Jay MA, Gilbert R. Special educational needs, social care and health. *Arch Dis Child* 2021; **106**: 83–5.

5 Hutchinson, Jo. Identifying pupils with special educational needs and disabilities. 2021. https://epi.org.uk/wp-content/uploads/2021/03/SEND-Indentification_2021-EPI.pdf (accessed Oct 5, 2021).

6 Department for Education. 01 - Pupils in all schools, by type of SEN provision - including independent schools and general hospital schools - 2016 to 2022. https://explore-education-statistics.service.gov.uk/ (accessed Jan 23, 2023).

7 Department for Education. Special educational needs in England, Academic Year 2021/22. 2022; published online June 16. https://explore-education-statistics.service.gov.uk/find-statistics/special-educational-needs-in-england#dataBlock-678da1e3-97e6-42f1-165e-08da47b0392d-tables (accessed Jan 23, 2023).

8 Roman-Urrestarazu A, van Kessel R, Allison C, Matthews FE, Brayne C, Baron-Cohen S. Association of Race/Ethnicity and Social Disadvantage With Autism Prevalence in 7 Million School Children in England. *JAMA Pediatr* 2021; **175**: e210054.

9 Strand S, Lindorff A. Ethnic disproportionality in the identification of Special Educational Needs (SEN) in England: Extent, causes and consequences. .

10 School funding statistics, Financial year 2022-23. https://explore-education-statistics.service.gov.uk/find-statistics/school-funding-statistics (accessed June 16, 2023).

11 Barnes M, Harrison E. The wellbeing of secondary school pupils with special educational needs: Reserach report. *Dep Educ* 2017. https://assets.publishing.service.gov.uk/government/uploads/system/uploads/attachment_data/file/626636/Wellbeing_and_SEN.pdf.

12 Libuy N, Gilbert R, Mc Grath-Lone L, Blackburn R, Etoori D, Harron K. Gestational age at birth, chronic conditions and school outcomes: a population-based data linkage study of children born in England. *Int J Epidemiol* 2022; : dyac105.

13 Mc Grath-Lone L, Etoori D, Gilbert R, Harron KL, Woodman J, Blackburn R. Changes in adolescents' planned hospital care during the COVID-19 pandemic: analysis of linked administrative data. *Arch Dis Child* 2022; **107**: e29–e29.

For peer review only - http://bmjopen.bmj.com/site/about/guidelines.xhtml

14 Cullen MA, Lindsay G, Hastings R, Denne L, Stanford C. Special Educational Needs in Mainstream Schools: Evidence Review. Education Endowment Foundation, 2020 https://eric.ed.gov/?id=ED612232 (accessed Jan 4, 2023).

15 Carroll J, Bradley L, Hannant P, Thompson A. SEN support: A rapid evidence assessment. *Dep Educ* 2017; published online July.

16 Moore DA, Russell AE, Matthews J, *et al.* School-based interventions for attention-deficit/hyperactivity disorder: A systematic review with multiple synthesis methods. *Rev Educ* 2018; **6**: 209–63.

17 Moore D, Benham-Clarke S, Kenchington R, *et al.* Improving Behaviour in Schools: Evidence Review. Education Endowment Foundation, 2019 https://eric.ed.gov/?id=ED612210 (accessed Jan 5, 2023).

18 Richardson M, Moore DA, Gwernan-Jones R, *et al.* Non-pharmacological interventions for attention-deficit/hyperactivity disorder (ADHD) delivered in school settings: systematic reviews of quantitative and qualitative research. *Health Technol Assess Winch Engl* 2015; **19**: 1–470.

19 John A, Friedmann Y, DelPozo-Banos M, Frizzati A, Ford T, Thapar A. Association of school absence and exclusion with recorded neurodevelopmental disorders, mental disorders, or self-harm: a nationwide, retrospective, electronic cohort study of children and young people in Wales, UK. *Lancet Psychiatry* 2022; **9**: 23–34.

20 Blackburn R, Ajetunmobi O, Mc Grath-Lone L, *et al.* Hospital admissions for stress-related presentations among school-aged adolescents during term time versus holidays in England: Weekly time series and retrospective cross-sectional analysis. *BJPsych Open* 2021; **7**. DOI:10.1192/bjo.2021.1058.

21 Benchimol EI, Smeeth L, Guttmann A, *et al.* The REporting of studies Conducted using Observational Routinely-collected health Data (RECORD) Statement. *PLOS Med* 2015; **12**: e1001885.

22 Mc Grath-Lone L, Libuy N, Harron K, *et al.* Data Resource Profile: The Education and Child Health Insights from Linked Data (ECHILD) Database. *Int J Epidemiol* 2022; **51**: 17–17f.

23 Libuy N, Harron K, Gilbert R, Caulton R, Cameron E, Blackburn R. Linking education and hospital data in England: linkage process and quality. *Int J Popul Data Sci* 2021; **6**. DOI:10.23889/ijpds.v6i1.1671.

24 Herbert A, Wijlaars L, Zylbersztejn A, Cromwell D, Hardelid P. Data Resource Profile: Hospital Episode Statistics Admitted Patient Care (HES APC). *Int J Epidemiol* 2017; **46**: 1093–1093i.

25 Zylbersztejn A, Gilbert R, Hardelid P. Developing a national birth cohort for child health research using a hospital admissions database in England: The impact of changes to data collection practices. *PLOS ONE* 2020; **15**: e0243843.

26 Harron K, Gilbert R, Cromwell D, Meulen J van der. Linking Data for Mothers and Babies in De-Identified Electronic Health Data. *PLOS ONE* 2016; **11**: e0164667.

27 Department for Education. Get Information about Schools (GIAS). https://www.get-information-schools.service.gov.uk/ (accessed Jan 5, 2023).

For peer review only

28 Fleming M, Salim EE, Mackay DF, *et al.* Neurodevelopmental multimorbidity and educational outcomes of Scottish schoolchildren: A population-based record linkage cohort study. *PLoS Med* 2020; **17**: e1003290.

29 Fleming M, Fitton CA, Steiner MFC, *et al.* Educational and Health Outcomes of Children Treated for Attention-Deficit/Hyperactivity Disorder. *JAMA Pediatr* 2017; **171**: e170691.

30 Reid SM, Meehan EM, Arnup SJ, Reddihough DS. Intellectual disability in cerebral palsy: a population-based retrospective study. *Dev Med Child Neurol* 2018; **60**: 687–94.

31 Fleming M, Fitton CA, Steiner MFC, *et al.* Educational and health outcomes of children and adolescents receiving antiepileptic medication: Scotland-wide record linkage study of 766 244 schoolchildren. *BMC Public Health* 2019; **19**: 595.

32 Zylbersztejn A, Stilwell PA, Zhu H, *et al.* Trends in hospital admissions during transition from paediatric to adult services for young people with learning disabilities or autism: population-based cohort study. *Lancet Reg Health – Eur* 2023; **24**. DOI:10.1016/j.lanepe.2022.100531.

33 Peppa M. The safety of influenza vaccination in pregnancy: Examining major congenital malformations as potential adverse outcomes using UK electronic health records. 2020; published online Oct 9. DOI:10.17037/PUBS.04658569.

34 Kinsner-Ovaskainen A, Lanzoni M, Garne E, *et al.* A sustainable solution for the activities of the European network for surveillance of congenital anomalies: EUROCAT as part of the EU Platform on Rare Diseases Registration. *Eur J Med Genet* 2018; **61**: 513–7.

35 Boyd PA, Haeusler M, Barisic I, Loane M, Garne E, Dolk H. Paper 1: The EUROCAT network—organization and processes†. *Birt Defects Res A Clin Mol Teratol* 2011; **91**: S2–15.

36 Burger RJ, Roseboom TJ, Ganzevoort W, *et al.* Gestational age and socio demographic factors associated with school performance at the age of 12 years, a population based study. *Paediatr Perinat Epidemiol*; **n/a**. DOI:10.1111/ppe.12990.

37 MacKay DF, Smith GCS, Dobbie R, Pell JP. Gestational Age at Delivery and Special Educational Need: Retrospective Cohort Study of 407,503 Schoolchildren. *PLOS Med* 2010; **7**: e1000289.

38 Office for Health Improvement and Disparities. 4. Children and young people. In: COVID-19 mental health and wellbeing surveillance: report. 2022. https://www.gov.uk/government/publications/covid-19-mental-health-and-wellbeing-surveillance-report/7-children-and-young-people (accessed Feb 1, 2023).

39 Etoori D, Harron KL, Mc Grath-Lone L, Verfürden ML, Gilbert R, Blackburn R. Reductions in hospital care among clinically vulnerable children aged 0–4 years during the COVID-19 pandemic. *Arch Dis Child* 2022; **107**: e31–e31.

40 Hernán MA, Robins JM. Using Big Data to Emulate a Target Trial When a Randomized Trial Is Not Available. *Am J Epidemiol* 2016; **183**: 758–64.

41 Hernán MA, Wang W, Leaf DE. Target Trial Emulation: A Framework for Causal Inference From Observational Data. *JAMA* 2022; **328**: 2446–7.

42 Greenland S, Pearl J, Robins JM. Causal Diagrams for Epidemiologic Research. *Epidemiology* 1999; **10**: 37.

For peer review only - http://bmjopen.bmj.com/site/about/guidelines.xhtml

43 Nguyen VG, Zylbergsztejn A, Harron K, *et al.* SEN support from the start of school and its impact on unplanned hospital utilisation in children with cleft lip and palate: a demonstration target trial emulation protocol using ECHILD. 2022; : 2022.04.01.22273280.

44 UCL. The ECHILD project website. 2020; published online Nov 17. https://www.ucl.ac.uk/child-health/research/population-policy-and-practice-research-and-teaching-department/cenb-clinical-20 (accessed Jan 19, 2023).

45 Waind E, Mc Grath-Lone L. The Education & Child Health Insights from Linked Data (ECHILD) Database Key messages from stakeholders. *ADR UK* 2021. https://www.adruk.org/fileadmin/uploads/adruk/Documents/ECHILD_Database_Key_messages_from_stakeholders_Aug_2021.pdf.

For peer review only

Figure 1 – Percentage of total children with recorded SEN provision per school year, January 2003 to 2022

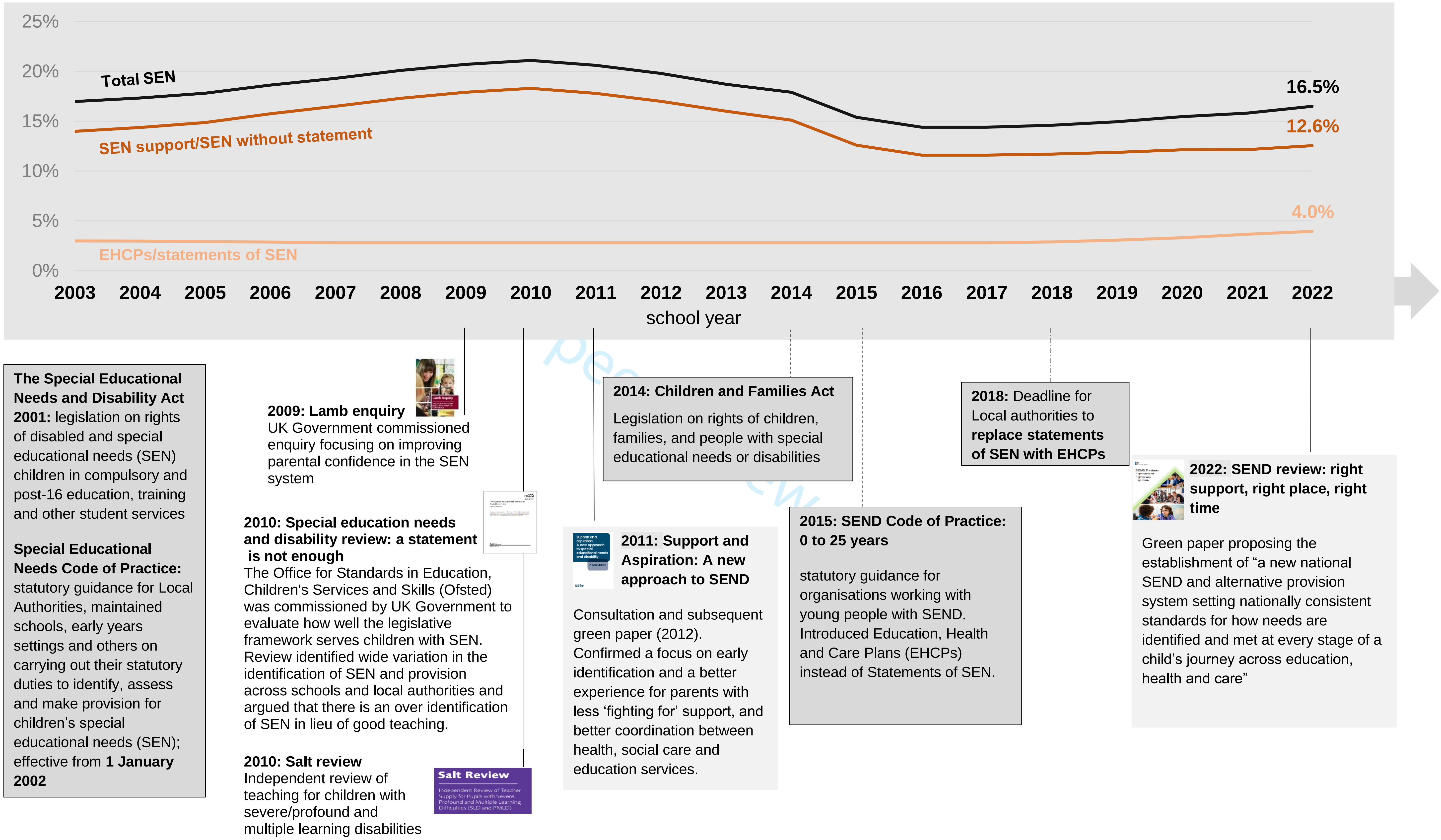

EHCPs: education, health and care plans; SEN: special educational needs; SEND: special educational needs or disability;

Figure 2 – Conceptual framework bringing together research questions (RQ) to be addressed by component studies of the HOPE research programme

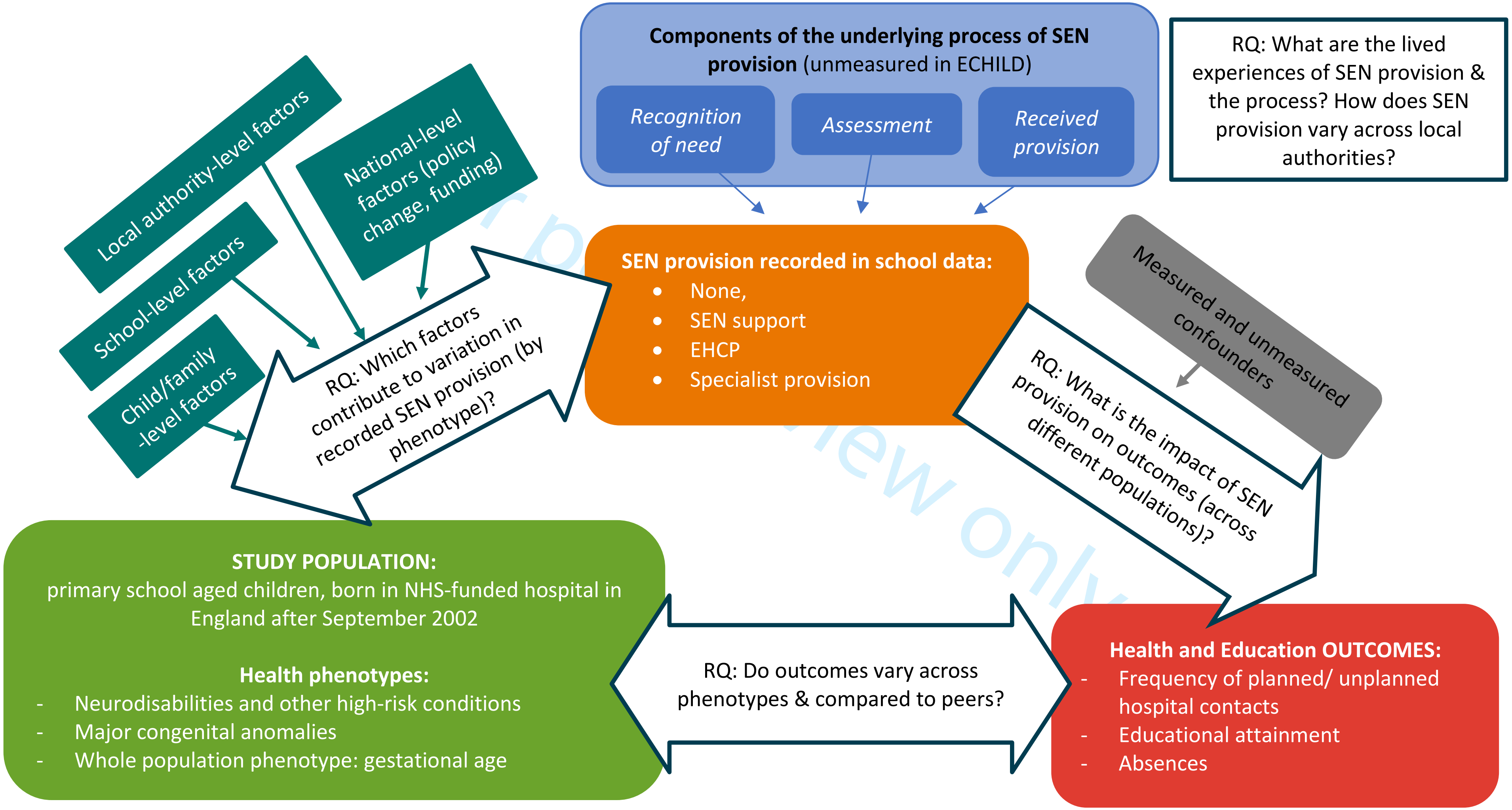

ECHILD: Education and Child Health Insights from Linked Data; EHCP: education, health and care plan; SEN: special educational needs;

Figure 3 – Coverage of ECHILD datasets, by academic year with refreshes

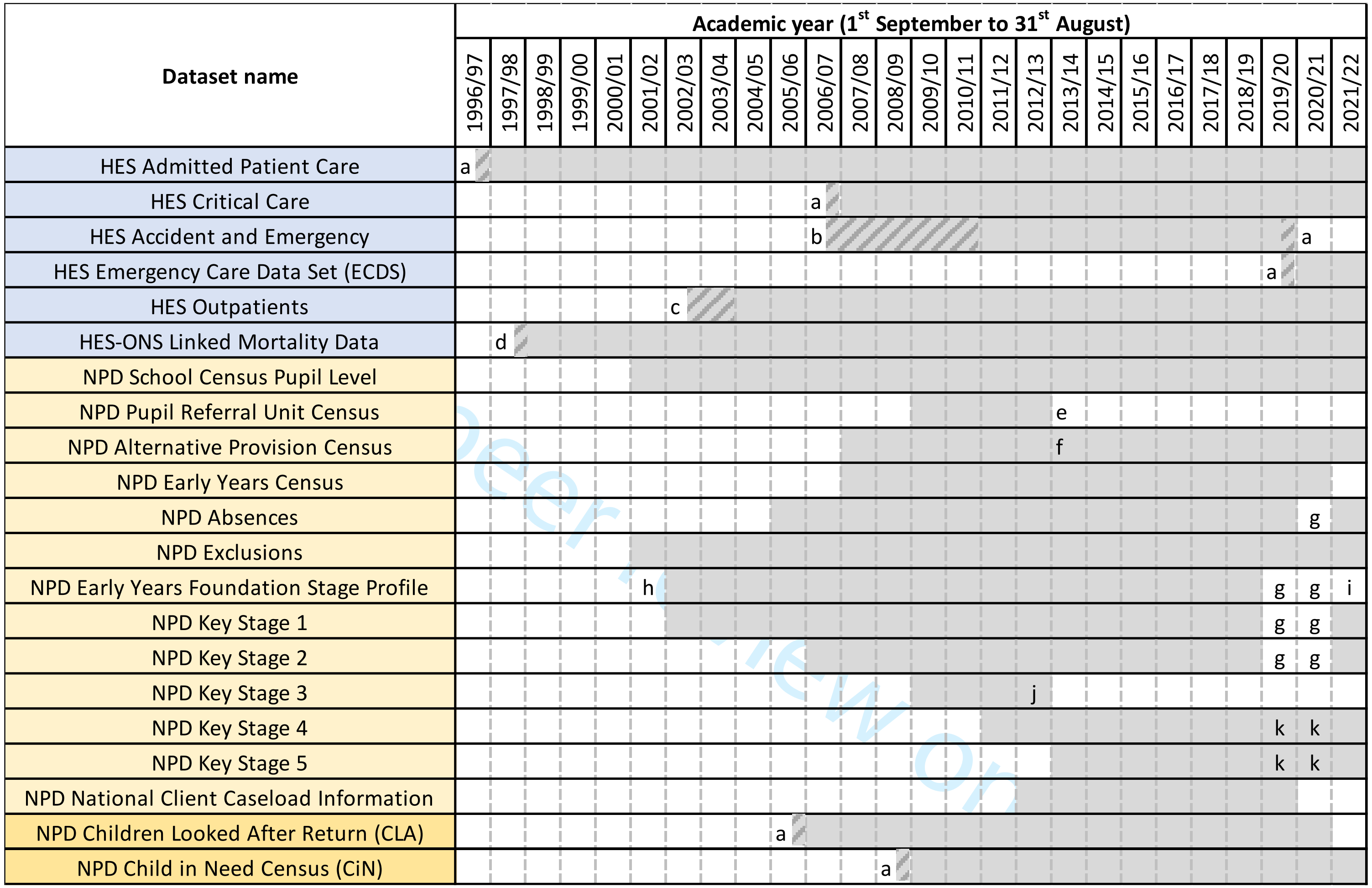

CiN: Child in Need Census, CLA: Children Looked After Return, HES: ECHILD: Education and Health Insights from Linked Data, Hospital Episode Statistics, NPD: National Pupil Database, ONS: Office for National Statistics.

a Partial coverage of an academic year as NPD social care data and HES data is collated by financial year (1st April to 31st March). b Partial coverage as HES Accident and Emergency data was experimental and did not have full national coverage. c Partial coverage as HES outpatient data was experimental and did not have full national coverage. d: Partial coverage of an academic year as ONS mortality data was first linked to HES in January 1998. e: The Pupil Referral Unit Census was subsumed in the School Census Pupil Level from 2013/14. f: The Early Years Census included 3- and 4-year olds between 2007/08 and 2012/13. From 2013/14, it includes 2-4 year olds. g: not collected to help reduce the burden on educational and care settings during the coronavirus (COVID-19) pandemic. h: Partial coverage as between the 2002/3 and 2005/6 academic years, data only on a 10% sample of children. i: to be included, but not available yet. j: Key Stage 3 assessments ceased after 2012/13. k: data not provided with standard institutional identifiers in 2019/20 – 2020/21, as evaluation of individual institutional performance is not permitted.

Appendix

HOPE study glossary

Confounding: The bias caused by shared causes of exposure and outcome.[1]

Confounder: A variable that can be used to adjust for confounding.[1]

Disability: under the Equality Act 2010 a disability is "a physical or mental impairment which has a long-term and substantial adverse effect on their ability to carry out normal day-to-day activities". Not all children with SEN have a disability, and not all disabled children have SEN, but there is significant overlap.[2,3]

Education, health and care plan, EHCP (known as SEN Statement prior to education reform in 2014/15): more intense provision arranged by local authorities, involving a legal document setting special measures provided by local authorities to meet a child's needs across education, health and social care.[2]

Health phenotypes: health conditions which can be indicated in administrative health records using diagnostic data (such as recorded diagnoses or procedures). In HOPE study we focus on health conditions associated with higher need for additional support for SEN than for their peers

Recorded SEN provision: Schools record information on children identified as needing SEN provision in school censuses returned to the Department for Education (DfE). Recorded SEN indicates identification of a child's primary need using fixed categories (see Box 1) and level of appropriate intervention (either SEN support or EHCP).[2] However, there is no centrally collated data on when and what type of intervention was received. Note that for simplicity we refer to *recorded SEN provision* as *SEN provision* in the protocol, although recorded SEN provision does not evidence that SEN provision is actually received or whether it is appropriate.[4]

Reasonable adjustments: schools have a duty to support pupils with medical conditions and make reasonable adjustments for children with disabilities, including the provision of auxiliary aids and services for disabled children. Not all children with disabilities have SEN, therefore reasonable adjustments are not considered SEN intervention.[2]

Special educational needs, SEN: a child has SEN if they have a health, learning or behavioural problems, that impact their ability to learn and require for special educational provision to be made for him or her.[2] Disability included in definition of SEN is a disability that prevents the child from using facilities generally provided for their peers in mainstream settings.[2]

Special educational needs or disability (SEND): Children with disabilities do not inevitably have SEN, but sometimes the term SEND is used to include children whose disabilities do impact their ability to access the mainstream school curriculum. The term SEND is also used by government departments in England to encompass all children with SEN, disabilities, or both.

SEN support (known as School Action and School Action Plus prior to education reform in 2014/15): SEN provisions arranged and funded by the school and provided almost entirely in mainstream schools. Prior to education reform in 2014/15, children could receive support as part of School Action or more intense support as part of School Action Plus. We consider all of these categories together for all analyses in the HOPE study.

SEN provision: provision different from or additional to that normally available to pupils of the same age[2]

For peer review only - http://bmjopen.bmj.com/site/about/guidelines.xhtml

Stages of the national curriculum[5]

The typical educational journey in England is segmented into a variety of blocks called “key stages”, during which children are expected to learn a set of subjects. At the end of each key stage, there is an assessment of child’s performance.

Key Stage 1: covers Year 1 (age 5 to 6) and Year 2 (age 6 to 7). In Year 1 there is a phonics screening check, whilst in Year 2 children take national tests in English reading and maths, and are assessed in maths, science, and English reading and writing by teachers.

Key Stage 2: covers Year 3 (age 7 to 8) to Year 6 (age 10 to 11). At the end of Key Stage 2, children take national tests in English reading, maths, and grammar, punctuation and spelling, and are assessed by teachers in English writing and science.

Key Stage 3: covers Years 7 (Age 11 to 12) to 9 (age 13 to 14). Year 7 is considered the start of “secondary” school. After 2008, the national curriculum does not require any assessments. Before, 2008 there were Standardised Assessment Tests (SATs).

Key Stage 4: covers educational Years 10 (age 14 to 15) and 11 (age 15 to 16). For most students, this includes being examined using the (International) General Certificate of Secondary Education (GCSE). Special schools provide NCFE qualifications which are highly reputable vocational and work-related courses, designed to accommodate the needs of employers for immediate full-time employment and allow students to progress to higher education to degree level.

Additional Results

Appendix Table 1 – Overview of number of children entering primary school (Year 1) captured in ECHILD

Academic year	All children in school in Year 1 (recorded in NPD)	All children in school in Year 1 linked to a HES record	All children in school in Year 1 linked to a birth admission in HES
2009/10	574,833	543,135 (94.5%)	449,166 (78.1%)
2010/11	587,163	553,705 (94.3%)	474,295 (80.8%)
2011/12	600,455	565,764 (94.2%)	489,041 (81.4%)
2012/13	620,754	584,215 (94.1%)	498,376 (80.3%)
2013/14	647,299	605,217 (93.5%)	504,161 (77.9%)
2014/15	645,292	601,087 (93.1%)	527,385 (81.7%)
2015/16	659,361	613,953 (93.1%)	556,171 (84.3%)
2016/17	669,197	623,097 (93.1%)	570,284 (85.2%)
total	5,004,354	4,690,173 (93.7%)	4,068,879 (81.3%)

HES=Hospital Episode Statistics, NPD=National Pupil Database

Search terms for literature review

("cognitive impairment*" OR "cognitive delay*" OR "learning disabilit*" OR "intellectual disabilit*" OR "*developmental disability* " OR "developmental delay*" OR "special education" OR "special educational needs" OR "special educational need" OR "additional learning need*" OR "special school*" OR "additional learning support")

AND ("systematic review" OR "meta-analysis" OR "meta analysis" OR "cohort study" OR "observational study" OR "population based" OR "population-based" OR "register study" OR "registry")

AND (child* or adolescen* or pupil* or teeange* or "school student*")

For peer review only - http://bmjopen.bmj.com/site/about/guidelines.xhtml

AND (congenital or "birth defect*" or chromosomal or "chronic disease" or "chronic condition*" or "long term condition*" or "long term disease" or "life limiting" or "end stage" or palliative or "liver disease" or asthma or cancer or malignan* or diabetes or obes* or encephalpath* or "cystic fibrosis" or "renal disease" or "kidney disease" or "heart disease" or cardiomyopathy or endocarditis or "lung disease" or "liver disease" or epilepsy or epileptic* or seizure* or "sickle cell" or "physical condition" or or illness or "brain injury" or "brain trauma" or cardiovascular or stroke or preterm or "birth weight" or birthweight or neonatal or gestation* or asphyxia or "spinal injury" or hydrocephalus or "nervous system" or autoimmune or eczema or arthritis or psoriasis or infection or medication)

References

1. Hernan MA, Robins JM. *Causal Inference: What If*. Boca Raton: Chapman & Hall/CRC.; 2020.
2. Department for Education. Special educational needs and disability code of practice: 0 to 25 years. Published online 2015. doi:DFE-00205-2013
3. Cullen MA, Lindsay G, Hastings R, et al. Special Educational Needs in Mainstream Schools: Evidence Review.
4. Jay MA, Grath-Lone LM, Gilbert R. Data Resource: the National Pupil Database (NPD). *International Journal of Population Data Science*. 2019;4(1). doi:10.23889/ijpds.v4i1.1101
5. Department for Education. The national curriculum. Accessed July 4, 2023. https://www.gov.uk/national-curriculum

For peer review only

BMJ Open

Evaluation of variation in special educational needs provision and its impact on health and education using administrative records for England: umbrella protocol for a mixed methods research programme

Journal:	*BMJ Open*
Manuscript ID	bmjopen-2023-072531.R2
Article Type:	Protocol
Date Submitted by the Author:	04-Oct-2023
Complete List of Authors:	Zylbersztejn, Ania; UCL Great Ormond Street Institute of Child Health Population Policy and Practice<br>Lewis, Kate; UCL Great Ormond Street Institute of Child Health Population Policy and Practice<br>Nguyen, Vincent; UCL Great Ormond Street Institute of Child Health Population Policy and Practice<br>Matthews, Jacob; University of Cambridge, Department of Psychiatry<br>Winterburn, Isaac; University of Cambridge, Department of Psychiatry<br>Karwatowska, Lucy; UCL Great Ormond Street Institute of Child Health Population Policy and Practice<br>Barnes, Sarah; University of Cambridge, Department of Psychiatry<br>Lilliman, Matthew; UCL Great Ormond Street Institute of Child Health Population Policy and Practice<br>Saxton, Jennifer; University of Cambridge, Department of Psychiatry<br>Stone, Antony; UCL Great Ormond Street Institute of Child Health Population Policy and Practice<br>Boddy, Kate; University of Exeter Medical School, Department of Health and Community Sciences<br>Downs, Johnny; King's College London, Institute of Psychiatry, Psychology and Neuroscience; South London and Maudsley NHS Foundation Trust<br>Logan, Stuart; University of Exeter Medical School, The Peninsula Childhood Disability Research Unit<br>Rahi, Jugnoo; UCL Great Ormond Street Institute of Child Health Population Policy and Practice; UCL Institute of Ophthalmology<br>Black-Hawkins, Kristine; University of Cambridge, Faculty of Education<br>Dearden, Lorraine; UCL Social Research Institute<br>Ford, Tamsin; University of Cambridge, Psychiatry<br>Harron, Katie; UCL Great Ormond Street Institute of Child Health Population Policy and Practice<br>De Stavola, Bianca ; UCL Great Ormond Street Institute of Child Health Population Policy and Practice<br>Gilbert, Ruth; UCL Great Ormond Street Institute of Child Health Population Policy and Practice
**Primary Subject Heading**:	Epidemiology

Secondary Subject Heading:	Public health, Qualitative research, Research methods, Paediatrics
Keywords:	EPIDEMIOLOGY, Health Equity, PUBLIC HEALTH, QUALITATIVE RESEARCH, STATISTICS & RESEARCH METHODS, Health informatics < BIOTECHNOLOGY & BIOINFORMATICS

SCHOLARONE™
Manuscripts

Evaluation of variation in special educational needs provision and its impact on health and education using administrative records for England: umbrella protocol for a mixed methods research programme

Ania Zylbersztejn[1], Kate Lewis[1], Vincent Nguyen[1], Jacob Matthews[2], Isaac Winterburn[2], Lucy Karwatowska[1], Sarah Barnes[2], Matthew Lilliman[1], Jennifer Saxton[2], Antony Stone[1], Kate Boddy[3], Johnny Downs[4,5], Stuart Logan[7], Jugnoo Rahi[1,8,9,10,11], Kristine Black-Hawkins[12], Lorraine Dearden[13], Tamsin Ford[2], Katie Harron[1], Bianca De Stavola[1], Ruth Gilbert[1]

[1]Population, Policy and Practice Research and Teaching Department, UCL Great Ormond Street Institute of Child Health, London, UK

[2]Department of Psychiatry, University of Cambridge, Cambridge, UK

[3]Department of Health and Community Sciences, University of Exeter Medical School

[4]Institute of Psychiatry, Psychology and Neuroscience, Kings College London, London, UK

[5]South London and Maudsley NHS Foundation Trust, London, UK

[7]The Peninsula Childhood Disability Research Unit, University of Exeter Medical School, Exeter, UK

[8]Great Ormond Street Hospital NHS Foundation Trust London, UK

[9]UCL Institute of Ophthalmology, London, UK

[10]NIHR Moorfields Biomedical Research Centre London, UK

[11]Ulverscroft Vision Research Group UCL London, UK

[12]Faculty of Education, University of Cambridge, Cambridge, UK

[13]UCL Social Research Institute, London, UK

Corresponding author: Dr Ania Zylbersztejn,

Population, Policy and Practice Research and Teaching Department, UCL Great Ormond Street Institute of Child Health, 30 Guilford Street, WC1N 1EH, London, UK

ania.zylbersztejn@ucl.ac.uk

Word count: 4500/4000

For peer review only

For peer review only - http://bmjopen.bmj.com/site/about/guidelines.xhtml

Abstract

Introduction: One-third of children in England have special educational needs (SEN) provision recorded during their school career. The proportion of children with SEN provision varies between schools and demographic groups, which may reflect variation in need, inequitable provision and/or system factors. There is scant evidence on whether SEN provision improves health and education outcomes.

Methods: The HOPE research programme uses administrative data from the Education and Health Insights from Linked Data – ECHILD – which contains data from all state schools, and contacts with NHS hospitals in England, to explore variation in SEN provision and its impact on health and education outcomes. This umbrella protocol sets out analyses across four work packages (WP). WP1 defined a range of "*health phenotypes*", that is health conditions expected to need SEN provision in primary school. Next, we describe health and education outcomes (WP1) and individual, school- and area-level factors affecting variation in SEN provision across different phenotypes (WP2). WP3 assesses the impact of SEN provision on health and education outcomes for specific health phenotypes using a range of causal inference methods to account for confounding factors and possible selection bias. In WP4 we review local policies and synthesise findings from surveys, interviews and focus groups of service users and providers to understand factors associated with variation in and experiences of identification, assessment, and provision for SEN. Triangulation of findings on outcomes, variation and impact of SEN provision for different health phenotypes in ECHILD, with experiences of SEN provision will inform interpretation of findings for policy, practice and families and methods for future evaluation.

Ethics and dissemination: Research ethics committees have approved the use of the ECHILD database and, separately, the survey, interviews and focus groups of young people, parents and service providers. These stakeholders will contribute to the design, interpretation and communication of findings.

For peer review only

Strengths and limitations of this study

Strengths:

- We use the ECHILD database, which comprises longitudinal histories of all hospital contacts funded by the National Health Service (NHS) and state-funded schooling. The population size (14.7 million children) and whole-of-England coverage of the ECHILD database allow exploration of outcomes for different health phenotypes over time, and by geographic area and sociodemographic characteristics.
- We define phenotypes in health data, which are recorded independently from processes in schools that lead to SEN provision. We apply different biostatistical and econometric methods to address potential confounding and selection bias.
- We use evidence from service users and providers to understand varying processes for identifying children who need and are provided with interventions for SEN. We triangulate evidence from analyses of ECHILD and mixed methods studies to strengthen the robustness of our findings and interpretation.

Limitations:

- The provision of SEN is based on organisational factors and on social and additional learning needs of children that are not objectively measured in health or education data before intervention. For example, the ECHILD database has few measures of the severity of impairment or behaviour problems.
- The ECHILD database does not capture NHS healthcare outside acute hospital settings, or education support at home or in the non-state funded sector. A record of SEN provision in school data does not evidence what (if any) SEN provision was received or whether it was appropriate, as these details are not recorded by schools.

For peer review only

For peer review only - http://bmjopen.bmj.com/site/about/guidelines.xhtml

Introduction

National policies in the UK and in many high-income countries require schools to make adaptations to meet the needs of children who have health, learning or behavioural problems, that impact their ability to learn. These children are referred to collectively as having special educational needs (SEN). Interventions and adjustments in schools for children with SEN are referred to as *SEN provision*, and are intended to improve inclusion and participation in education and support children's health and wellbeing (see Appendix 1 for details of SEN provision in England).[1] Since 2015, approximately one in six children in England are recorded as receiving any SEN provision each year (see Figure 1),[2] and one-third of all children have a record of any SEN provision at least once during their time in education.[3,4]

SEN provision across England is widely regarded as inequitable.[2,4–6] The proportion of pupils with SEN support (more common type of provision, arranged and funded by the schools, see Appendix 1) ranged from 7.3% to 17.1% across local authorities in 2018/19. The proportion with Education, Health and Care Plans (EHCPs, which involve additional, more intensive, and higher cost provision for children whose needs cannot be fully met by SEN support, arranged and partly funded by local authorities[2,7]) ranged from 0.8% to 5.0%.[8] Allocation of SEN provision is associated with a variety of factors. According to a recent report, a key factor determining SEN provision is the school, particularly school's previous rates of SEN provision, academy status and previous school inspection outcomes.[4] Other factors include the proportion of academised primary schools and rates of pupils eligible for free school meals at local authority level-and pupil-level factors such as attainment at school entry (age 5), ethnic group, child's first language and contacts with social care.[4–6] The annual proportion of children with recorded SEN provision has declined over time, from 20% in school year ending in August 2010 to 14% in 2016. This change seems partly related to the Children and Families Act in 2014 and Special Educational Needs and Disability Code of Practice implemented in 2015, and to reduced funding to local authorities from 2010 (Figure 1).[2,9]

Compared to their peers, children with recorded SEN provision have higher rates of chronic physical and mental health conditions and hospitalisations, and have lower self-reported wellbeing.[10–12] Recent evidence reviews found that classroom-based SEN interventions improved children's social, emotional wellbeing and reduced challenging behaviour, and contributed to better mental health outcomes.[13,14] For children with attention deficit hyperactivity disorder (ADHD), systematic reviews of randomised, or quasi randomised controlled trials (RCTs) of interventions similar to SEN provision found improvements in behaviour.[10,15–17] However, there is a lack of RCTs or representative observational comparative studies of the impact of SEN provision, as delivered in routine practice, on health outcomes for a range of health conditions.

Robust evidence that SEN provision improves educational outcomes for pupils with SEN is also scarce.[13] There is moderate evidence that SEN provision in primary schools improves literacy difficulties, socio-emotional development and language and communication.[14] A recent evidence review found a weak but positive impact of inclusive education involving additional support for those with additional learning needs on academic outcomes among pupils without SEN provision.[13] Evidence from population-based observational studies suggests that SEN provision is associated with fewer absences and exclusions among children and young people with neurodisability or mental health conditions.[18]

Given the large proportion of children with SEN provision indicated in school records, the high costs of SEN provision, and static school funding per pupil since 2010,[9] evidence is needed to quantify how SEN provision varies across England and to guide effective intervention to groups of children who are most likely to benefit. The Health Outcomes of young People in Education (HOPE) research programme aims to address these gaps through novel proof-of-concept analyses of the Education and Child Health Insights from Linked Data (ECHILD) database, which links data from all state schools, and contacts with NHS hospitals in England, and mixed methods (surveys, interviews and

For peer review only - http://bmjopen.bmj.com/site/about/guidelines.xhtml

focus groups with families and service providers, and document analyses) to understand experiences of service users and providers. We will assess two central research questions: i) which factors contribute to variation in SEN provision in England; and ii) what is the impact of SEN provision on health and education outcomes? We will address these research questions for a range of health conditions associated with increased need for SEN provision, which we refer to as *health phenotypes*.

This umbrella protocol sets out the research plan for the HOPE programme to address these two core questions. We describe four parallel work packages (WP), and in brief, the component studies contributing to each WP. The conceptual framework and proposed research questions are illustrated in Figure 2. Separate study protocols for each component study will be pre-registered on NIHR Open Research platform (https://openresearch.nihr.ac.uk/) and follow relevant reporting guidelines from the EQUATOR Network (https://www.equator-network.org/, e.g. analyses using ECHILD will be reported using RECORD guidelines for studies using linked administrative data).[19]

In the first work package (WP1), we define a range of *health phenotypes*, that is health conditions captured in hospital records that are expected to need SEN provision in primary school. We explore how health and education outcomes vary for children with different health phenotypes and compared to unaffected peers. In WP2, we describe how child, social and area-level factors affect variation in SEN provision within phenotypes. In WP3, we apply a range of causal inference methods to address confounding factors (informed by WP2) and possible selection bias to assess the impact of SEN provision on outcomes for children with selected health phenotypes (defined in WP1), also considering timing, duration and level of provision. The ECHILD database contains termly records indicating provision for SEN, but no information about whether or when any provision was actually received, its type or quality. WP4 applies mixed methods to understand geographic variation in local policies and the underlying processes of identification, assessment and provision, and how these processes are experienced by families. Triangulation of findings on outcomes (WP1), variation in (WP2) and impact of (WP3) SEN provision for children with different health phenotypes from ECHILD, with findings on local policies and experiences of SEN provision (WP4) will inform findings for policy, practice and families and methods for future evaluation.

The HOPE research programme started in August 2021 and is expected to end in April 2025. The research programme is ongoing and elements of the programme that have already been completed at the time of publication of this protocol are highlighted in the methods and analyses below.

Methods and analysis

Data sources and study measures for WP1-3

ECHILD database

The ECHILD database links routinely collected administrative data on health and education in England. Currently ECHILD includes all children and young people aged 0–24 years in England who were born between 1 September 1995 and 31 August 2021 (approximately 14.7 million individuals).[20] Health and education datasets were linked by NHS England using a multi-step deterministic linkage algorithm, described in detail elsewhere.[21] Linkage rates were high and increased over time (92% of school pupils born in academic year 1990/91 were linked to a hospital record, compared to 99% of pupils born in 2004/05).[21]

Health data consists of Hospital Episode Statistics (HES), a national database that includes dated information on all National Health Service (NHS) acute hospital care and mortality data (see Figure 3 for details of data coverage by academic year).[22] Nearly all children born in England are born in NHS hospitals (97%) but HES excludes births in private hospitals or at home. Children can be followed from their birth admission through all subsequent NHS hospital contacts.[23,24]

For peer review only - http://bmjopen.bmj.com/site/about/guidelines.xhtml

Education records are collated in the National Pupil Database (NPD) and include information on children's registrations in schools, attainment scores at ages 5, 7 11, 16 and 18 (see Study Glossary in Appendix 2 for details), and number of half-day absences and exclusions in each 13-week term. SEN provision is recorded each term (annually prior to 2005/6) for all children in state-funded education (93% of all children) from the academic year starting in September 2001 onwards (see Figure 3).[25] Education data captures the category of recorded SEN provision (SEN support or EHCP) and main reason for SEN provision. Since 2014/15, reasons for SEN include language or communication, moderate or severe learning disability, autism, sensory impairment, physical disability, or social, emotional or mental health needs (see Appendix 1 for details). We are in the process of enhancing ECHILD with school characteristics, such as type of school (e.g.: mainstream or special) or teacher-pupil ratio, from a range of publicly available data.[26–28]

All analyses of ECHILD across WP1-3 will use shared definitions of study population, SEN provision, and outcomes, as described in more detail below. The analyses of the ECHILD database will be a proof of concept, restricted to children attending primary school and three groups of health phenotypes (described in detail below). Analyses of all age groups, and all possible health phenotypes, types of SEN provision, and possible comparisons are beyond the scope of the HOPE programme, but can be informed by our methods. We will publish our methods and code to enable others to reproduce and extend our analyses using ECHILD.

Study cohort & health phenotypes

Our target population is primary school aged children (enrolled in school in Year 1 aged 5/6 and followed to Year 6 aged 10/11), who were born in an NHS hospital in England and had a birth admission recorded in HES data from September 2002 onwards. We follow up all children from birth and use information about risk factors at birth (such as gestational age, birth weight) and health phenotypes recorded in health data before the start of primary school.[23,24] We can then evaluate exposure to SEN provision at the start of primary school and the impact on subsequent health and education outcomes. We analyse three groups of health phenotypes as we hypothesise that the impact of SEN provision on health and education outcomes will vary for children with different health phenotypes.

Not all children in English primary schools have a birth record in HES. Between 1st September 2009 and 31st August 2017 there were 5,004,354 children entering primary school (recorded in school census in Year 1, aged 5-6 years old), of whom 94% had a linked HES record and 80% had a linked birth record (the linkage rate increased over time, see Appendix Table 1 in Appendix 3). As these numbers are large, we can focus on specific or even uncommon phenotypes.

We will use clusters of coded clinical information in the ECHILD database to define *health phenotypes* that represent health conditions associated with learning impairment or need for additional educational support. We have defined three groups of health phenotypes which capture populations with different levels of need for SEN provision:

a) *Neurodisability and other high-risk conditions*

The first group of health phenotypes comprises neurologic conditions or complex systemic health problems reported to be associated with learning impairment or behaviours that require SEN provision. These include neurodisability such as autism or learning disabilities,[29,30] cerebral palsy,[31] or epilepsy.[32] The list of health phenotypes has been derived from an overview of systematic reviews and population-based observational studies (see Appendix 3 for overview of search terms) and discussions with clinical experts and service providers. We developed coding algorithms for these health phenotypes based on combinations of diagnostic and procedure codes, where possible from previously validated code lists. As part of validation, we will compare the cumulative incidence and mortality rates by age for each health phenotype with external population

For peer review only - http://bmjopen.bmj.com/site/about/guidelines.xhtml

studies (for example, from national surveys and disease registries) and changes over time to assess consistency of recorded diagnoses in hospital records and to further refine the phenotyping algorithm. A detailed phenotyping paper is in preparation.

Preliminary findings for children with neurodisability or high-risk conditions include 50 high-risk health phenotypes recorded in hospital records before the age of 5. These account for approximately 5% of all children starting primary school in 2008/9-2018/19, 10% of children with any recorded SEN provision during primary school and 30% of those with an EHCP. Some of the included conditions (such as autism or learning disability) are likely to be under-reported in hospital records.[33]

b) Major congenital anomalies

The second group includes children with major congenital anomalies (MCAs), as children with MCAs are likely to require support from specialist services and have a diagnosis recorded in hospital admissions records, creating a reliable phenotype defined before entry to primary school among children whose need for SEN provision is likely to vary.[34] We are using a code list of ICD-10 diagnoses developed by EUROCAT – a European Congenital Anomaly Registry (https://eu-rd-platform.jrc.ec.europa.eu/eurocat),[35,36] which groups MCAs into 12 body system groups and includes 25 specific subgroups.

Our preliminary findings suggest that children with MCAs recorded in the first year of life account for 3.5% of the primary school population in 2008/9-2018/19, 5.5% of children with any recorded SEN provision during primary school, and 13.6% of those receiving EHCPs in mainstream school or attending a specialist school.

c) Whole population phenotype: gestational age

Finally, we are using week of gestational age at birth to assess the gradient in impact of SEN provision across the whole population of children, stratified according to different levels of underlying need. This approach is supported by the finding that each week of birth before 40 weeks of gestation is associated with reduced school attainment scores and an increased risk of SEN intervention.[11,37,38] Approximately 4.5% of children in primary school in 2008/9-2018/19 were born preterm (at <37 weeks' gestation), accounting for 6.0% of children with any recorded SEN provision during primary school, and 8.4% of those receiving EHCPs.

Health and education outcomes

We focus on outcomes that can be measured in hospital and education data: unplanned (A&E and unplanned admissions) and planned hospital contacts (planned or elective admissions and outpatient appointments), school attainment (as proxy measure for cognitive function), and rates of school absences (see Box 1 for study measure definitions).

Follow up of outcomes will cease before the onset of the COVID-19 pandemic. COVID-19 had a significant impact on the wellbeing of young people.[39] Lockdowns in England affected children's access to school and the frequency of hospital contacts captured in ECHILD data. Planned and unplanned admissions, and outpatient appointments reduced substantially during the COVID-19 pandemic, with the largest reductions in children with indicators of vulnerability (such as preterm birth, a chronic condition, recorded SEN or social care record).[12,40] School attainment measures were not collected during the pandemic to help reduce the burden on educational and care settings. In the HOPE programme, we will therefore limit our analyses to outcomes recorded before the start of COVID-19 pandemic.

For peer review only - http://bmjopen.bmj.com/site/about/guidelines.xhtml

Recorded SEN provision

Schools record information on children identified as needing SEN provision (SEN support or EHCP) in school censuses returned to the Department for Education (DfE). We refer to this recording as *SEN provision* throughout the protocol, although we acknowledge that an indication of "SEN provision" in educational records does not evidence that SEN provision is actually received or whether it is appropriate, as these data are not recorded by schools.[25]

We categorise SEN provision in a descending hierarchy for a specified time period (e.g., during school Year 1 or across all of primary school; details shown in Box 1), separating any enrolment in a special school or alternative provision (where the vast majority of children have recorded SEN provision), an EHCP in mainstream school, SEN support in mainstream school, and no recorded SEN provision. These categories have been selected due to differences in the presumed type of provision, in the criteria for provision (e.g.: formal assessment is required for EHCP but not for SEN support) and substantial differences in associated costs.[2]

SEN provision changed following Government education reforms in 2014/15, when EHCPs gradually replaced Statements of SEN, and SEN support replaced SEN without Statement (which grouped two levels of provision: School Action and School Action Plus). We will report changes in recorded SEN over time and address potential impacts of these changes in the design of analyses.

Box 1: key study measures derived from ECHILD database

Measures derived from health data:

Accident & Emergency (A&E) department contact rate: defined as the number of days with at least one A&E contact, divided by person-time at risk during the study period (e.g.: time from start of Year 1 until the end of Year 6 or death).

Unplanned/planned admission rate: defined as the number of unplanned or planned hospital admissions in NHS-funded hospitals in England, divided by person-time at risk during the study period. Admissions will be classified as planned/unplanned according to the admission method recorded in the first episode of care. Consecutive admissions with re-admission within one day of discharge (for example, hospital transfers) will be treated as part of the same admission. Time spent in hospital during an admission will be taken out of the person-time at risk as once a child is in hospital they cannot be at risk of a new admission.

Outpatient department (OPD) appointments and attendances: defined as the number of days with at least one OPD contact, divided by person-time at risk during the study period (e.g.: time from start of Year 1 until the end of Year 6 or death).

Measures derived from education data:

Absence rate: schools are required to take attendance registers twice a day, for morning and afternoon sessions. Absence rates are defined in line with the definition used by Department for Education as the total number of absent sessions (including authorised and unauthorised absences) divided by the total number of possible sessions during the study period.

Standardised attainment measures: We derive standardised attainment measures using recorded scores from national tests in reading, writing and maths at the end of Year 2 (aged 7, Key Stage 1) and at the end of Year 6 (aged 11, Key Stage 2, see Appendix 2 for details of Key Stages). Standardised test scores are calculated using mean and standard deviation of the test scores of all pupils in a given academic year. We will present the proportion of children not assessed (i.e.: did not have an assessment record) and average score for those assessed by study population.

For peer review only - http://bmjopen.bmj.com/site/about/guidelines.xhtml

School readiness indicators: we use scores from teacher assessments of children's development across multiple areas of learning, carried out in the final term before Year 1 (Early Year Foundation Stage Profile, EYFSP, see Appendix 2 for details). Standardised EYFSP scores are calculated using mean and standard deviation of the EYFSP scores of all pupils in a given academic year. We will present the percentage of children who were not assessed (i.e.: did not have an assessment record), the proportion of children not reaching Good Level of Development (GLD, defined by Department for Education using a subset of EYFPS scores) and average scores for those assessed by study population.

SEN provision: we use 4 categories (which may be merged for some analyses) in the following descending hierarchy for a specified time period (e.g.: Year 1):

1) Enrolment in specialist provision (including special school or alternative),
2) EHCP (including 'Statement of SEN' or 'EHCP') in mainstream school,
3) SEN support (including 'School Action', 'School Action Plus' or 'SEN support') in mainstream school,
4) No SEN provision.

Data sources for WP4

National online survey

Research for WP4 to-date includes three online surveys aimed at (1) children and young people, (2) parents/carers and (3) service providers (health, education and local authority professionals). The surveys document variation in local experiences of identification and assessment of need, and provision of SEN intervention. Detailed information on survey design and findings will be published separately. In brief, the surveys were developed through a scoping review to identify previous questionnaires and co-designed with stakeholder groups of young people, parents/carers and professionals working in education or health with children who have SEN. Each survey underwent three rounds of extensive piloting with the respective advisory groups from the HOPE study. This helped to: i) ensure that the questions and response options matched the lived experience of participants, ii) test accessibility and usability for respective participants, iii) ensure no technical difficulties existed when completing the survey across multiple device types (e.g smartphone, laptop). Data were collected using REDCap and the surveys were disseminated via social media (Twitter, Instagram, and Facebook), and through professional networks (GOV.UK Notify service, Parent and Carer forums, and stakeholder group contacts). These networks were used to maximise the recruitment of all three groups from each of the nine administrative health regions in England.

In total 1,714 participants took part from across England including: 77 young people aged 11-27 years, 772 parents and carers, and 865 service providers (those working in/closely with education settings, the health services, local authorities). Short summaries of the key findings at the time of submission of this protocol from initial analyses are published on the study website (https://dev.psychiatry.cam.ac.uk/hope-study-health-outcomes-for-young-people-throughout-education/) and more detailed papers are in preparation.

Qualitative studies of children, young people, parent/carer and practitioner experience

We are conducting two qualitative studies (including interviews and focus groups) to explore the experiences and beliefs of children, young people, parents/carers and practitioners about SEN

For peer review only - http://bmjopen.bmj.com/site/about/guidelines.xhtml

provision to assist the interpretation of our quantitative analyses using ECHILD. Both qualitative studies recruited from the survey respondents who agreed to recontact in the national survey described above.

a) *Children, young people and parents / carers*

We conducted semi-structured interviews with children and young people (supported by carer if they wished) and separately with parents / carers. The topic guide covered the identification, assessment and provision of support for their or their child's special educational needs using a time-line approach. 16 interviews with children and young people and 22 with parents / carers were completed between May and June 2023 and will be analysed using a framework approach (see Analysis Plan section for details).[41]

b) *Practitioner working in health and education*

We will conduct three focus groups of up to 10 practitioners on each of the major areas identified by our stakeholder groups (identification, assessment and provision of SEN intervention), providing a total of 9 focus groups involving up to 90 practitioners working in relation to SEN across health, education, local authority or social services. Each area of focus will have its own topic guide and each series of focus groups will be facilitated by a team of two researchers and a parent observer. The latter will provide their opinion of the discussion in focused debriefing sessions after each focus group. Analysis will follow a framework approach,[41] and parent observers will contribute to the interpretation of our findings at a final joint meeting.

Analysis plan

Work package 1: Describing health and education outcomes

We will carry out separate descriptive studies for each of the three groups of health phenotypes. We will estimate rates of planned and unplanned hospital contacts and educational outcomes during primary school for children with and without each health phenotype. We will use appropriate generalised linear models for each outcome (e.g.: Poisson regression for rates, logistic regression for binary outcomes). Findings from these analyses will indicate whether there are differences in outcomes across subgroups within each health phenotype and compared to their peers, to inform analyses in WP3.

Work package 2: Variation in recorded SEN provision

We will use ECHILD to understand variation in SEN provision for children with different phenotypes. We will examine how proportions of children with recorded SEN provision changed over time for children with different health phenotypes (e.g.: MCAs), and the percentage in their variation that is explained by factors at the individual-, school- and local authority-level using appropriate multi-level regression models. These analyses will determine whether the impact of SEN provision (examined in WP3) can be evaluated using natural policy experiment designs (e.g. due to changes in policy over time) and instrumental variable analysis. These analyses will also generate findings on variation in SEN provision according to child level demographic, social and service use characteristics.

Work package 3: Impact of SEN provision on outcomes

We will use a range of biostatistical and econometric methods to explore the impact of SEN provision on health and educational outcomes across selected health phenotypes, and triangulate findings from analyses using different methods (briefly described below). First, we will examine the

For peer review only - http://bmjopen.bmj.com/site/about/guidelines.xhtml

impact of recorded SEN provision at a given point in time, in the first year of compulsory education (Year 1). Second, we will assess the impact of the duration of SEN provision, appropriately controlling for likely time-varying factors that may be affected by SEN provision (informed by findings from WP2) and additionally influence future SEN provision. We anticipate separate studies focussing on specific health phenotypes that represent conditions that are relatively similar in their need for SEN and well characterised in health data. For example, cleft lip and palate has been selected from the MCA phenotypic group as it is reliably denoted by diagnostic and operation codes. We will select a well-defined phenotype within the neurodisability group and we also plan to compare exposure to SEN provision for children within defined strata of gestational age at birth across the whole population.

For all causal analyses, we will use the target trial emulation (TTE) framework to guide the creation of study cohorts that correspond to the specific phenotypes and exposure levels (categories of SEN provision) of interest.[42,43] TTE consists of first designing an ideal pragmatic trial that would address the question of interest, and then emulating it as closely as possible using observational data. The advantage of this approach is the avoidance of biases in the design stage, e.g. immortal time bias and prevalent-case bias, that have affected real-world-data studies in the past.[42] Directed Acyclic Graphs (DAGs) will be used to draw our assumptions about the causal structures influencing what we are studying and to identify relevant confounding variables.[44] An outline of the components of our causal investigations is given in Appendix Table 2 (Appendix 3) where these steps are illustrated using the exemplar of one MCA phenotypes: children born with cleft lip and palate. The study protocol for this study has been registered as a preprint.[45]

As ECHILD comprises observational data, a major challenge for each of our causal investigations will be how to address the bias introduced by confounding by indication, as well as possible selection bias due to incomplete linkage across health and education databases. We will contrast estimates of the impact of SEN provision on health and educational outcomes using a variety of complementary methods that rely on measuring all of the confounders, such as regression adjustment, g-computation, inverse probability weighting of marginal structural models (with different approaches to modelling the propensity score), or econometric methods that try to deal with unmeasured confounding by exploiting natural experiments (such as differences-in-differences or interrupted time series), or instrumental variables.

Work Package 4: Understanding policy variation and lived experiences of SEN provision

We will conduct an overarching synthesis of the findings from the national survey, parent and child / young person interviews and focus groups with practitioners, in relation to the quantitative findings from WP1-3. The planning of this work draws from previous similar triangulation of quantitative and qualitative systematic reviews.[17] We will first create a matrix to demonstrate where these different data sources provide similar or conflicting signals. We will then work inductively from the surveys, focus group and interview findings to generate hypotheses about contextual elements that may influence the recording of data in ECHILD, or health outcomes of SEN provision. This approach draws from “complexity theory” which assumes that any psychosocial intervention must be considered in terms of the larger environment in which it is located.[46] We will also work deductively from the results of analyses in WP1-3 to explore potential relationships between SEN provision and health outcomes, as well as exploring what WP4 findings suggest about factors associated with health outcomes in SEN provision. The aim of this approach is to clarify potential explanations for the findings of WP1-3 and to inform future work in this area. Analysis under the two approaches described above will proceed iteratively and in parallel.

To understand local variation in SEN provision we reviewed publicly available documents on the support available for local children with SEN, referred to as “local offer”. We are assessing the

For peer review only - http://bmjopen.bmj.com/site/about/guidelines.xhtml

quality of available information against 51 criteria outlined within the SEND Code of Practice to determine to what extent Local Authorities in England are providing clear, comprehensive, accessible and up-to-date information about available SEN provision and how to access it. Second, we are examining reports from all of the local area SEND inspections published by the Office for Standards in Education, Children's Services and Skills (OFSTED) over the past seven years to examine how effectively local authorities fulfil responsibilities for children and young people with SEN. By collating these documents and assessing their commonalities and differences, we aim to gain an understanding of variation in good and bad practice in SEN provision.

Public involvement and engagement

The HOPE study was developed in response to consultations about the need for the ECHILD database with parents and charities supporting children with chronic health conditions and their families.[47,48] We have established three stakeholder groups: young people, parents / carers and professionals working in education or health with children who have SEN. We are collaborating with staff in schools to enable young people with additional learning needs or disabilities to contribute to these advisory groups. In addition, we are iteratively presenting our study plans and preliminary results to parents/carers and young people through research advisory groups at UCL and University of Exeter. These consultations are contributing evidence to all four work packages and dissemination of the research. The HOPE Study Steering Committee includes two parents of children with disability and will adhere to NIHR requirements for payment for time and expenses of lay contributors.

Ethics

Existing research ethics approval has been granted for analyses of the ECHILD database for the purposes set out in the HOPE study (20/EE/0180). Data access is also controlled by agreements with NHS Digital and the DfE. The data contain no identifiers or sensitive dates and data can only be used within the Office for National Statistics Secure Research Environment by approved researchers, with strict statistical disclosure controls of all outputs of analyses (e.g. tables or figures). Details are published here in our privacy notice (https://www.ucl.ac.uk/child-health/research/population-policy-and-practice-research-and-teaching-department/cenb-clinical-4#).

Separate ethics approval has been approved for the mixed-methods research (national survey, interviews and focus groups) involving service users (young people and parents) and service providers (PRE:2021.058). Parents consented for their own involvement and also for their child if under the age of 16. Young people aged 16 or over consented and younger children were asked for assent to their participation using a similar process.

Dissemination

We will present preliminary findings to diverse audiences (academics, analysts at DfE and Department of Health and Social Care, and our stakeholder groups as well as other groups of service users and providers) through seminars, question and answer sessions, workshops and consultations during the study. We will incorporate feedback into final outputs, which will include peer reviewed journal articles, the final study report to funder, and short briefing reports and infographics for non-academics published on the study website.

We will publish our methods and code to enable others to reproduce and extend our analyses using ECHILD. ECHILD can be accessed by accredited researchers through application via the ECHILD team (www.ucl.ac.uk/child-health/echild) and the Research Accreditation Panel. Meta-data and code relating to the HOPE study will be signposted on the study website and made available in the ONS secure environment and in code repository (including on ECHILD GitHub page: https://github.com/UCL-ECHILD). We will hold workshops to promote wider use of findings from the HOPE study for causal analyses of education interventions on health. Examples from the HOPE study will be incorporated into short courses on causal methods and on how to use the ECHILD database.

For peer review only - http://bmjopen.bmj.com/site/about/guidelines.xhtml

The HOPE study aims to build the evidence base for fairer and more effective SEN provision and, by informing national and local policy and the public and changing practice, to improve health and education outcomes of children with SEN.

For peer review only

For peer review only - http://bmjopen.bmj.com/site/about/guidelines.xhtml

Figure captions

Figure 1 – Percentage of total children with recorded SEN provision per school year, January 2003 to 2022 (based on DfE statistics)[8,49,50]

Legend:

DfE: Department for Education; EHCPs: education, health and care plans; SEN: special educational needs; SEND: special educational needs or disability;

Figure 2 – Conceptual framework bringing together research questions (RQ) to be addressed by component studies of the HOPE research programme

Legend: ECHILD: Education and Child Health Insights from Linked Data; EHCP: education, health and care plan; SEN: special educational needs;

Figure 3 – Coverage of ECHILD datasets, by academic year with refreshes

Legend:
CiN: Child in Need Census, CLA: Children Looked After Return, HES: ECHILD: Education and Health Insights from Linked Data, Hospital Episode Statistics, NPD: National Pupil Database, ONS: Office for National Statistics.

a Partial coverage of an academic year as NPD social care data and HES data is collated by financial year (1st April to 31st March). b Partial coverage as HES Accident and Emergency data was experimental and did not have full national coverage. c Partial coverage as HES outpatient data was experimental and did not have full national coverage. d: Partial coverage of an academic year as ONS mortality data was first linked to HES in January 1998. e: The Pupil Referral Unit Census was subsumed in the School Census Pupil Level from 2013/14. f: The Early Years Census included 3- and 4-year olds between 2007/08 and 2012/13. From 2013/14, it includes 2-4 year olds. g: not collected to help reduce the burden on educational and care settings during the coronavirus (COVID-19) pandemic. h: Partial coverage as between the 2002/3 and 2005/6 academic years, data only on a 10% sample of children. i: to be included, but not available yet. j: Key Stage 3 assessments ceased after 2012/13. k: data not provided with standard institutional identifiers in 2019/20 – 2020/21, as evaluation of individual institutional performance is not permitted.

For peer review only - http://bmjopen.bmj.com/site/about/guidelines.xhtml

Footnotes

Abbreviations

A&E: accident and emergency department

ADHD: attention deficit hyperactivity disorder

ASD: autistic spectrum disorders

ATNT: Average treatment effect in the non-treated

ATT: Average treatment effect in the treated

CLP: cleft lip and palate

DAG: Directed Acyclic Graph

DfE: Department for Education

ECHILD: Education and Child Health Insights from Linked Data

EHCP: education, health and care plan

HES: Hospital Episode Statistics

IPW: Inverse Probability Weighting

ITE: Interventional treatment effect

MCA: Major Congenital Anomaly

MSM: Marginal Structural Models

NHS: National Health Service

NPD: National Pupil Database

OFSTED: the Office for Standards in Education, Children's Services and Skills

RCT: randomised controlled trial

SEN: special educational needs

TTE: target trial emulation

For peer review only

Author contributions

RG is the principal investigator of the HOPE research programme and KH, TF, LD and BDS lead each of the four work packages, KBH, JD, SL, JR are co-investigators of the programme. AZ and RG produced the first draft of this manuscript, AZ, KL and ML created figures for the manuscript. AZ, KL, VN, AS, ML are responsible for preparing ECHILD data for analyses. JM, IW, SB, JS, KBH and TF are responsible for conceptualisation and implementation of mixed methods studies. BDS, LD, KL, VN, and LK are responsible for design and implementation of quantitative causal inference analyses using ECHILD database. AZ, KH, RG, KL are responsible for design and implementation of descriptive analyses using ECHILD database, with clinical input from JD, SL, JR on defining study populations. JS, JM, IW, KB, KBH, TF, RG, KH, AZ, VN were involved in public engagement activities to inform the study. All authors edited the manuscript. All authors have contributed to and are responsible for the final design of the study. All authors read and approved the final manuscript.

For peer review only - http://bmjopen.bmj.com/site/about/guidelines.xhtml

Acknowledgments

We would like to acknowledge the contribution of members of the HOPE study steering committee.

We are grateful to the Office for National Statistics (ONS) for providing the trusted research environment for the ECHILD Database. We gratefully acknowledge all children and families whose de-identified data are used in this research.

The ECHILD Database uses data from the Department for Education (DfE). The DfE does not accept responsibility for any inferences or conclusions derived by the authors. This work also uses data provided by patients and collected by the National Health Service as part of their care and support. Source data can also be accessed by researchers by applying to NHS Digital. This work was produced using statistical data accessed via the ONS Secure Research Service. The use of this data in this work does not imply the endorsement of the ONS in relation to the interpretation or analysis of the statistical data. This work uses research datasets which may not exactly reproduce National Statistics aggregates. This presentation has been cleared for publication by ONS (STATS17409, STATS15494).

Competing Interests

We declare no competing interests.

Funding

This study/project is funded by the National Institute for Health Research (NIHR) under its Programme Grants for Applied Research Programme (Grant Reference number NIHR202025). The views expressed are those of the author(s) and not necessarily those of the NIHR or the Department of Health and Social Care.

For peer review only

For peer review only - http://bmjopen.bmj.com/site/about/guidelines.xhtml

References

1 Department for Education. Special educational needs and disability code of practice: 0 to 25 years. Published Online First: 2015. doi: DFE-00205-2013

2 The National Audit Office. Support for pupils with special educational needs and disabilities in England. Published Online First: 2019.

3 Jay MA, Gilbert R. Special educational needs, social care and health. *Arch Dis Child*. 2021;106:83–5.

4 Hutchinson, Jo. Identifying pupils with special educational needs and disabilities. Published Online First: 2021.

5 Roman-Urrestarazu A, van Kessel R, Allison C, *et al.* Association of Race/Ethnicity and Social Disadvantage With Autism Prevalence in 7 Million School Children in England. *JAMA Pediatr*. 2021;175:e210054.

6 Strand S, Lindorff A. Ethnic disproportionality in the identification of Special Educational Needs (SEN) in England: Extent, causes and consequences.

7 Department for Education. Special educational needs in England, Academic Year 2021/22. 2022. https://explore-education-statistics.service.gov.uk/find-statistics/special-educational-needs-in-england#dataBlock-678da1e3-97e6-42f1-165e-08da47b0392d-tables (accessed 23 January 2023)

8 Department for Education. 01 - Pupils in all schools, by type of SEN provision - including independent schools and general hospital schools - 2016 to 2022. https://explore-education-statistics.service.gov.uk/ (accessed 23 January 2023)

9 School funding statistics, Financial year 2022-23. https://explore-education-statistics.service.gov.uk/find-statistics/school-funding-statistics (accessed 16 June 2023)

10 Barnes M, Harrison E. The wellbeing of secondary school pupils with special educational needs: Reserach report. *Dep Educ*. Published Online First: 2017.

11 Libuy N, Gilbert R, Mc Grath-Lone L, *et al.* Gestational age at birth, chronic conditions and school outcomes: a population-based data linkage study of children born in England. *Int J Epidemiol*. 2022;dyac105.

12 Mc Grath-Lone L, Etoori D, Gilbert R, *et al.* Changes in adolescents' planned hospital care during the COVID-19 pandemic: analysis of linked administrative data. *Arch Dis Child*. 2022;107:e29–e29.

13 Cullen MA, Lindsay G, Hastings R, *et al.* Special Educational Needs in Mainstream Schools: Evidence Review. Education Endowment Foundation 2020. https://eric.ed.gov/?id=ED612232 (accessed 4 January 2023)

14 Carroll J, Bradley L, Hannant P, *et al.* SEN support: A rapid evidence assessment. *Dep Educ*. 2017.

For peer review only

For peer review only - http://bmjopen.bmj.com/site/about/guidelines.xhtml

15 Moore DA, Russell AE, Matthews J, *et al.* School-based interventions for attention-deficit/hyperactivity disorder: A systematic review with multiple synthesis methods. *Rev Educ*. 2018;6:209–63.

16 Moore D, Benham-Clarke S, Kenchington R, *et al.* Improving Behaviour in Schools: Evidence Review. Education Endowment Foundation 2019. https://eric.ed.gov/?id=ED612210 (accessed 5 January 2023)

17 Richardson M, Moore DA, Gwernan-Jones R, *et al.* Non-pharmacological interventions for attention-deficit/hyperactivity disorder (ADHD) delivered in school settings: systematic reviews of quantitative and qualitative research. *Health Technol Assess Winch Engl*. 2015;19:1–470.

18 John A, Friedmann Y, DelPozo-Banos M, *et al.* Association of school absence and exclusion with recorded neurodevelopmental disorders, mental disorders, or self-harm: a nationwide, retrospective, electronic cohort study of children and young people in Wales, UK. *Lancet Psychiatry*. 2022;9:23–34.

19 Benchimol EI, Smeeth L, Guttmann A, *et al.* The REporting of studies Conducted using Observational Routinely-collected health Data (RECORD) Statement. *PLOS Med*. 2015;12:e1001885.

20 Mc Grath-Lone L, Libuy N, Harron K, *et al.* Data Resource Profile: The Education and Child Health Insights from Linked Data (ECHILD) Database. *Int J Epidemiol*. 2022;51:17–17f.

21 Libuy N, Harron K, Gilbert R, *et al.* Linking education and hospital data in England: linkage process and quality. *Int J Popul Data Sci*. 2021;6. doi: 10.23889/ijpds.v6i1.1671

22 Herbert A, Wijlaars L, Zylbersztejn A, *et al.* Data Resource Profile: Hospital Episode Statistics Admitted Patient Care (HES APC). *Int J Epidemiol*. 2017;46:1093–1093i.

23 Zylbersztejn A, Gilbert R, Hardelid P. Developing a national birth cohort for child health research using a hospital admissions database in England: The impact of changes to data collection practices. *PLOS ONE*. 2020;15:e0243843.

24 Harron K, Gilbert R, Cromwell D, *et al.* Linking Data for Mothers and Babies in De-Identified Electronic Health Data. *PLOS ONE*. 2016;11:e0164667.

25 Jay MA, Grath-Lone LM, Gilbert R. Data Resource: the National Pupil Database (NPD). *Int J Popul Data Sci*. 2019;4. doi: 10.23889/ijpds.v4i1.1101

26 Department for Education. Get Information about Schools (GIAS). https://www.get-information-schools.service.gov.uk/ (accessed 5 January 2023)

27 Department for Education. Statistics: school workforce. GOV.UK. 2023. https://www.gov.uk/government/collections/statistics-school-workforce (accessed 2 October 2023)

28 Department for Education. School and college performance data in England. 2022. https://www.find-school-performance-data.service.gov.uk/ (accessed 2 October 2023)

29 Fleming M, Salim EE, Mackay DF, *et al.* Neurodevelopmental multimorbidity and educational outcomes of Scottish schoolchildren: A population-based record linkage cohort study. *PLoS Med*. 2020;17:e1003290.

For peer review only - http://bmjopen.bmj.com/site/about/guidelines.xhtml

30 Fleming M, Fitton CA, Steiner MFC, *et al.* Educational and Health Outcomes of Children Treated for Attention-Deficit/Hyperactivity Disorder. *JAMA Pediatr*. 2017;171:e170691.

31 Reid SM, Meehan EM, Arnup SJ, *et al.* Intellectual disability in cerebral palsy: a population-based retrospective study. *Dev Med Child Neurol*. 2018;60:687–94.

32 Fleming M, Fitton CA, Steiner MFC, *et al.* Educational and health outcomes of children and adolescents receiving antiepileptic medication: Scotland-wide record linkage study of 766 244 schoolchildren. *BMC Public Health*. 2019;19:595.

33 Zylbersztejn A, Stilwell PA, Zhu H, *et al.* Trends in hospital admissions during transition from paediatric to adult services for young people with learning disabilities or autism: population-based cohort study. *Lancet Reg Health – Eur*. 2023;24. doi: 10.1016/j.lanepe.2022.100531

34 Peppa M. *The safety of influenza vaccination in pregnancy: Examining major congenital malformations as potential adverse outcomes using UK electronic health records*. 2020. https://doi.org/10.17037/PUBS.04658569

35 Kinsner-Ovaskainen A, Lanzoni M, Garne E, *et al.* A sustainable solution for the activities of the European network for surveillance of congenital anomalies: EUROCAT as part of the EU Platform on Rare Diseases Registration. *Eur J Med Genet*. 2018;61:513–7.

36 Boyd PA, Haeusler M, Barisic I, *et al.* Paper 1: The EUROCAT network—organization and processes†. *Birt Defects Res A Clin Mol Teratol*. 2011;91:S2–15.

37 Burger RJ, Roseboom TJ, Ganzevoort W, *et al.* Gestational age and socio demographic factors associated with school performance at the age of 12 years, a population based study. *Paediatr Perinat Epidemiol*. ;n/a. doi: 10.1111/ppe.12990

38 MacKay DF, Smith GCS, Dobbie R, *et al.* Gestational Age at Delivery and Special Educational Need: Retrospective Cohort Study of 407,503 Schoolchildren. *PLOS Med*. 2010;7:e1000289.

39 Office for Health Improvement and Disparities. 4. Children and young people. *COVID-19 mental health and wellbeing surveillance: report*. 2022. https://www.gov.uk/government/publications/covid-19-mental-health-and-wellbeing-surveillance-report/7-children-and-young-people (accessed 1 February 2023)

40 Etoori D, Harron KL, Mc Grath-Lone L, *et al.* Reductions in hospital care among clinically vulnerable children aged 0–4 years during the COVID-19 pandemic. *Arch Dis Child*. 2022;107:e31–e31.

41 Gale NK, Heath G, Cameron E, *et al.* Using the framework method for the analysis of qualitative data in multi-disciplinary health research. *BMC Med Res Methodol*. 2013;13:117.

42 Hernán MA, Robins JM. Using Big Data to Emulate a Target Trial When a Randomized Trial Is Not Available. *Am J Epidemiol*. 2016;183:758–64.

43 Hernán MA, Wang W, Leaf DE. Target Trial Emulation: A Framework for Causal Inference From Observational Data. *JAMA*. 2022;328:2446–7.

44 Greenland S, Pearl J, Robins JM. Causal Diagrams for Epidemiologic Research. *Epidemiology*. 1999;10:37.

For peer review only - http://bmjopen.bmj.com/site/about/guidelines.xhtml

45 Nguyen VG, Zylbergsztejn A, Harron K, *et al.* SEN support from the start of school and its impact on unplanned hospital utilisation in children with cleft lip and palate: a demonstration target trial emulation protocol using ECHILD. 2022;2022.04.01.22273280. https://doi.org/10.1101/2022.04.01.22273280

46 Montuori A. The Complexity of Transdisciplinary Literature Reviews. *Complicity Int J Complex Educ*. 2013;10. doi: 10.29173/cmplct20399

47 UCL. The ECHILD project website. 2020. https://www.ucl.ac.uk/child-health/research/population-policy-and-practice-research-and-teaching-department/cenb-clinical-20 (accessed 19 January 2023)

48 Waind E, Mc Grath-Lone L. The Education & Child Health Insights from Linked Data (ECHILD) Database Key messages from stakeholders. *ADR UK*. Published Online First: 2021.

49 Department for Education. Special educational needs in England: January 2015. GOV.UK. 2015. https://www.gov.uk/government/statistics/special-educational-needs-in-england-january-2015 (accessed 21 September 2023)

50 Ofsted. *The special educational needs and disability review. A statement is not enough*. 2010. https://www.gov.uk/government/publications/special-educational-needs-and-disability-review

For peer review only

For peer review only - http://bmjopen.bmj.com/site/about/guidelines.xhtml

Figure 1 – Percentage of total children with recorded SEN provision per school year, January 2003 to 2022 (based on DfE statistics)[8,45,46]

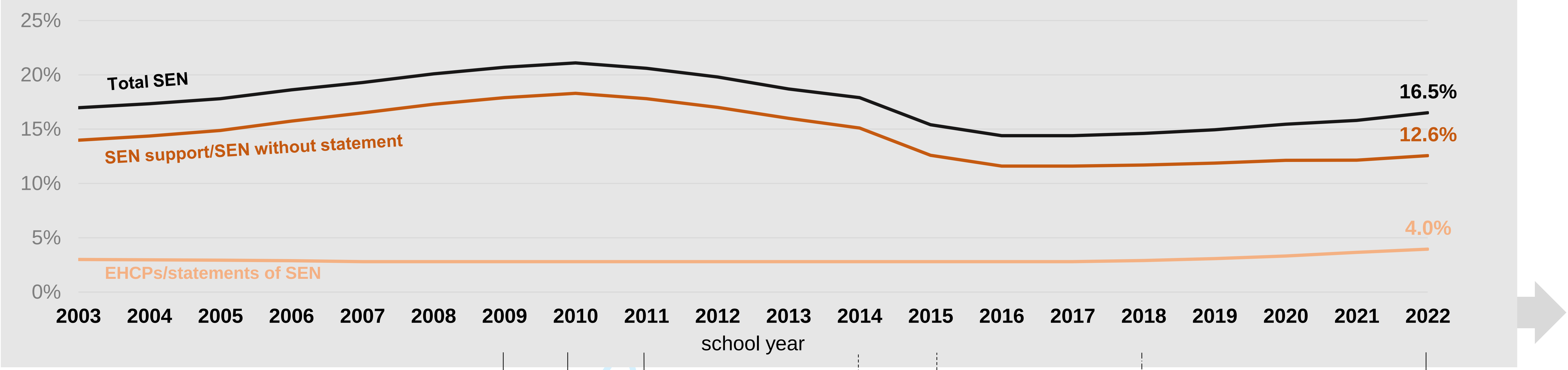

The Special Educational Needs and Disability Act 2001: legislation on rights of disabled and special educational needs (SEN) children in compulsory and post-16 education, training and other student services

Special Educational Needs Code of Practice: statutory guidance for Local Authorities, maintained schools, early years settings and others on carrying out their statutory duties to identify, assess and make provision for children's special educational needs (SEN); effective from **1 January 2002**

2009: Lamb enquiry
UK Government commissioned enquiry focusing on improving parental confidence in the SEN system

2010: Special education needs and disability review: a statement is not enough
The Office for Standards in Education, Children's Services and Skills (Ofsted) was commissioned by UK Government to evaluate how well the legislative framework serves children with SEN. Review identified wide variation in the identification of SEN and provision across schools and local authorities and argued that there is an over identification of SEN in lieu of good teaching.

2010: Salt review
Independent review of teaching for children with severe/profound and multiple learning disabilities

2011: Support and Aspiration: A new approach to SEND

Consultation and subsequent green paper (2012). Confirmed a focus on early identification and a better experience for parents with less 'fighting for' support, and better coordination between health, social care and education services.

2014: Children and Families Act

Legislation on rights of children, families, and people with special educational needs or disabilities

2015: SEND Code of Practice: 0 to 25 years

statutory guidance for organisations working with young people with SEND. Introduced Education, Health and Care Plans (EHCPs) instead of Statements of SEN.

2018: Deadline for Local authorities to **replace statements of SEN with EHCPs**

2022: SEND review: right support, right place, right time

Green paper proposing the establishment of "a new national SEND and alternative provision system setting nationally consistent standards for how needs are identified and met at every stage of a child's journey across education, health and care"

DfE: Department for Education; EHCPs: education, health and care plans; SEN: special educational needs; SEND: special educational needs or disability;

Figure 2 – Conceptual framework bringing together research questions to be addressed by each work package (WP) of the HOPE research programme

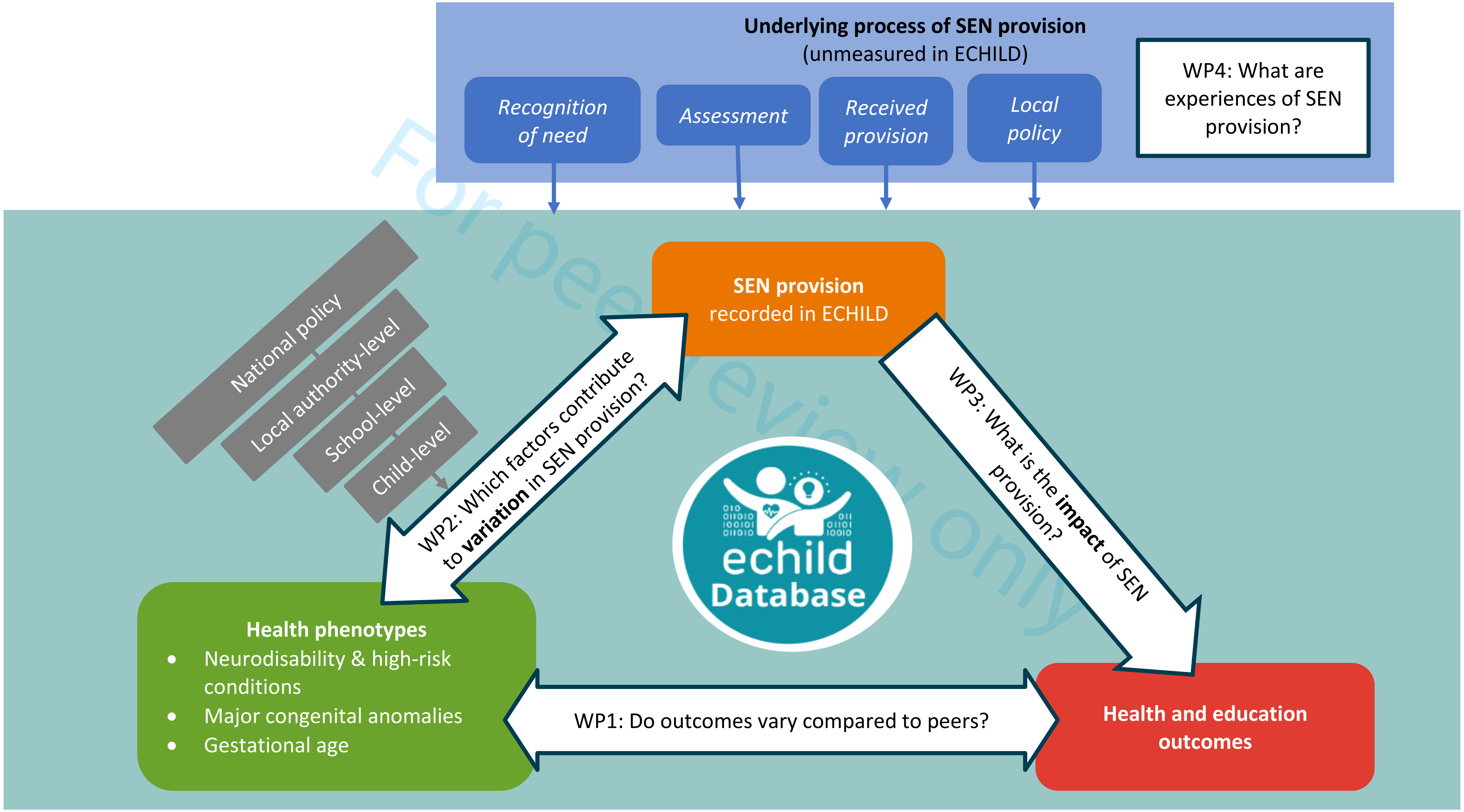

ECHILD: Education and Child Health Insights from Linked Data; EHCP: education, health and care plan; SEN: special educational needs;

For peer review only - http://bmjopen.bmj.com/site/about/guidelines.xhtml

Figure 3 – Coverage of ECHILD datasets, by academic year with refreshes

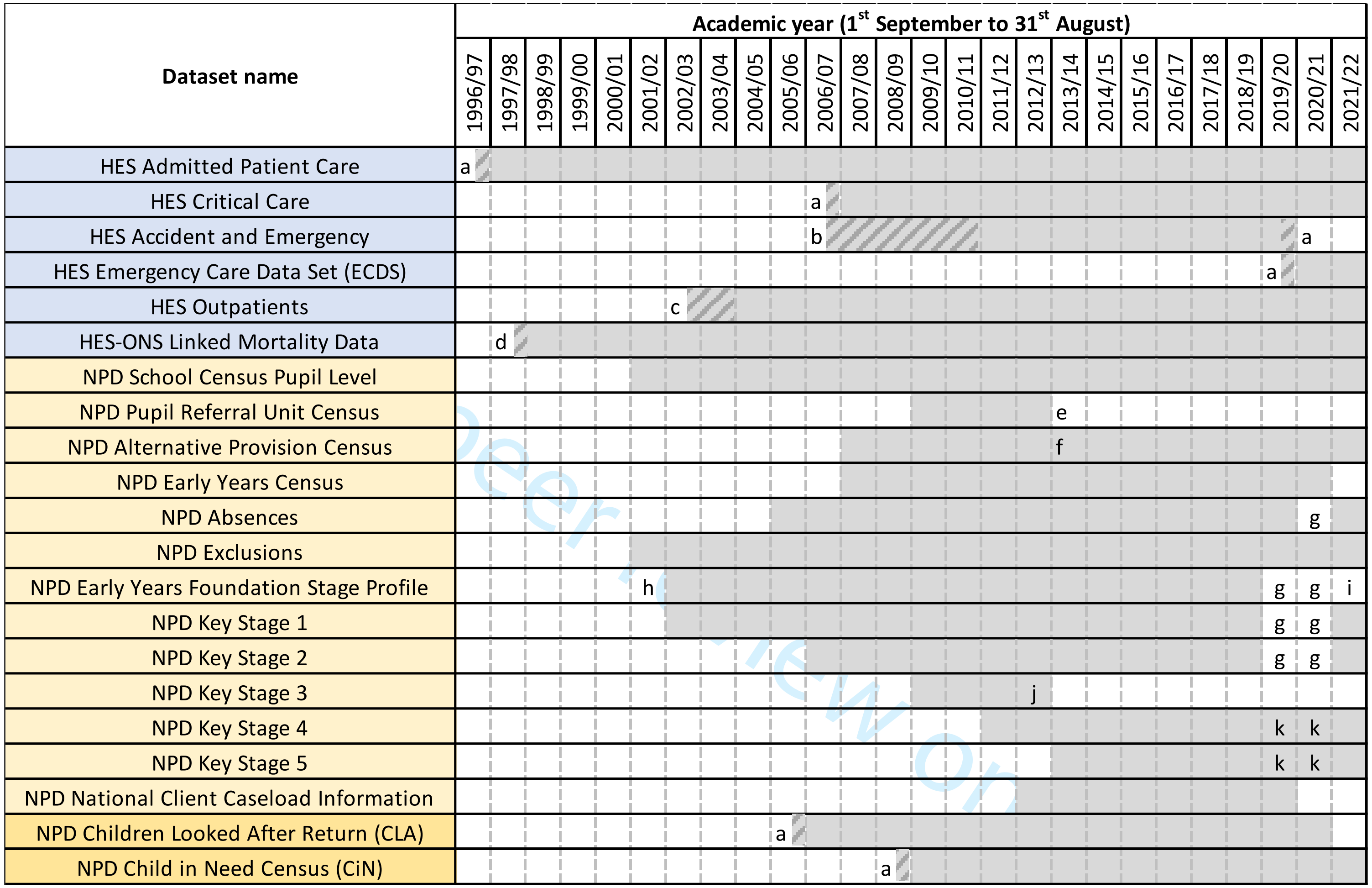

CiN: Child in Need Census, CLA: Children Looked After Return, HES: ECHILD: Education and Health Insights from Linked Data, Hospital Episode Statistics, NPD: National Pupil Database, ONS: Office for National Statistics.

a Partial coverage of an academic year as NPD social care data and HES data is collated by financial year (1st April to 31st March). b Partial coverage as HES Accident and Emergency data was experimental and did not have full national coverage. c Partial coverage as HES outpatient data was experimental and did not have full national coverage. d: Partial coverage of an academic year as ONS mortality data was first linked to HES in January 1998. e: The Pupil Referral Unit Census was subsumed in the School Census Pupil Level from 2013/14. f: The Early Years Census included 3- and 4-year olds between 2007/08 and 2012/13. From 2013/14, it includes 2-4 year olds. g: not collected to help reduce the burden on educational and care settings during the coronavirus (COVID-19) pandemic. h: Partial coverage as between the 2002/3 and 2005/6 academic years, data only on a 10% sample of children. i: to be included, but not available yet. j: Key Stage 3 assessments ceased after 2012/13. k: data not provided with standard institutional identifiers in 2019/20 – 2020/21, as evaluation of individual institutional performance is not permitted.

For peer review only - http://bmjopen.bmj.com/site/about/guidelines.xhtml

Appendix 1: Overview of Special Educational Needs provision in England

National policies in the UK and in many high-income countries require schools to make adaptations to meet the needs of children who have health, learning or behavioural problems, that impact their ability to learn; these children are referred to collectively as having special educational needs (SEN). In England, children with SEN "*have a significantly greater difficulty in learning than the majority of others of the same age, or have a disability which prevents them from making use of facilities generally provided by mainstream schools*" (see the study glossary in Appendix 2 below).[1]

There are four broad areas of SEN that state-funded schools are required to support: communication and social interactions, cognition and learning, social, emotional and mental health and sensory or physical disabilities (see Box 1). Interventions and adjustments in schools for children with SEN, referred to as *SEN provision*, are intended to improve inclusion and participation in education and support children's health and wellbeing.[1] Approximately 7% of children in England attend private schools each year,[2] which do not have the same legal obligations regarding SEN identification and provision.

*Box 1 – Broad areas of special educational needs (SEN) that schools should plan for, with sub-categories set out by the Special Educational Needs and Disability code of practice in England[1] (at the time of publication)**

Communication and social interactions
- Speech, language and communication needs
- Autistic Spectrum Disorders

Cognition and learning:
- Moderate learning difficulties
- Severe learning difficulties
- Profound and multiple learning difficulties
- Specific learning difficulties (that is learning difficulties affecting one or more specific aspects of learning, such as dyslexia, dyscalculia or dyspraxia)

Social, emotional and mental health*
- Examples could be attention-deficit/hyperactivity disorder, behavioural difficulties, anxiety, depression, eating disorders

Sensory and/or physical disabilities:
- Vision impairment
- Hearing impairment
- Multi-sensory impairment
- Physical disability requiring additional ongoing support and/or equipment to access all the opportunities available to their peers

**Note that the sub-categories changed in 2014/15 following reforms to SEN system: "Social, emotional and mental health difficulties" were introduced in 2014/15, while "Behaviour, Emotional & Social Difficulties" were removed.*

Since 2015, approximately one in six children in England are recorded by schools as receiving any SEN provision each year,[3] and one-third of all children have a record of any SEN provision at least once during their time in education.[4,5] There are two categories of SEN provision offered in England,

For peer review only - http://bmjopen.bmj.com/site/about/guidelines.xhtml

SEN support and *Education, Health and Care Plans (EHCPs)*. These categories were introduced following Government education reforms in 2014/15, replacing the older categories of *School Action/School Action Plus* (together referred to as *SEN without Statement*) and *Statements of SEN*, respectively (see study glossary in Appendix 2 for details).

The majority of pupils with any recorded SEN provision receive *SEN support*. SEN support is arranged and funded by the schools and can include short-term interventions such as speech or language therapy or extra support for reading. The first assessment for SEN support is usually carried out by the school's teachers, Special Educational Needs Coordinator (SENCo), or after class teachers, who seek to identify children making less than expected educational progress or with additional social needs relative to their peers. In 2018/19 (the last academic year before the COVID-19 pandemic), 11.9% of pupils had SEN support recorded, with the vast majority provided in mainstream schools.[6]

A smaller proportion of children receive an EHCP, which involves additional and more intensive provision arranged and partly funded by local authorities for children whose needs cannot be fully met by SEN support.[3,7] Support may range from extra help by a part-time teaching assistant to full-time care by multiple staff in a special school.[1] An assessment for an EHCP can be requested by parents, schools or health or social care professionals. The assessment is carried out by the local authority, who are required to fill in a legal document setting out the special measures to be provided to meet the child's needs across education, health and social care.[3,7] In 2018/19, 3.1% of pupils had a record of an EHCP, half of whom were enrolled in a special school (1.6% of all children).[6]

Appendix 2: HOPE study glossary

Confounding: The bias caused by shared causes of exposure and outcome.[8]

Confounder: A variable that can be used to adjust for confounding.[8]

Disability: under the Equality Act 2010 a disability is "a physical or mental impairment which has a long-term and substantial adverse effect on their ability to carry out normal day-to-day activities". Not all children with SEN have a disability, and not all disabled children have SEN, but there is significant overlap.[1,9]

Education, health and care plan, EHCP (known as SEN Statement prior to education reform in 2014/15): more intense provision arranged by local authorities, involving a legal document setting special measures provided by local authorities to meet a child's needs across education, health and social care.[1]

Health phenotypes: health conditions which can be indicated in administrative health records using diagnostic data (such as recorded diagnoses or procedures). In HOPE study we focus on health conditions associated with higher need for additional support for SEN than for their peers

Recorded SEN provision: Schools record information on children identified as needing SEN provision in school censuses returned to the Department for Education (DfE). Recorded SEN indicates identification of a child's primary need using fixed categories (see Box 1) and level of appropriate intervention (either SEN support or EHCP).[2] However, there is no centrally collated data on when and what type of intervention was received. Note that for simplicity we refer to *recorded SEN provision* as *SEN provision* in the protocol, although recorded SEN provision does not evidence that SEN provision is actually received or whether it is appropriate.[2]

Reasonable adjustments: schools have a duty to support pupils with medical conditions and make reasonable adjustments for children with disabilities, including the provision of auxiliary aids and services for disabled children. Not all children with disabilities have SEN, therefore reasonable adjustments are not considered SEN intervention.[1]

For peer review only - http://bmjopen.bmj.com/site/about/guidelines.xhtml

Special educational needs, SEN: a child has SEN if they have a health, learning or behavioural problems, that impact their ability to learn and require for special educational provision to be made for him or her.[1] Disability included in definition of SEN is a disability that prevents the child from using facilities generally provided for their peers in mainstream settings.[1]

Special educational needs or disability (SEND): Children with disabilities do not inevitably have SEN, but sometimes the term SEND is used to include children whose disabilities do impact their ability to access the mainstream school curriculum. The term SEND is also used by government departments in England to encompass all children with SEN, disabilities, or both.

SEN support (known as School Action and School Action Plus prior to education reform in 2014/15): SEN provisions arranged and funded by the school and provided almost entirely in mainstream schools. Prior to education reform in 2014/15, children could receive support as part of School Action or more intense support as part of School Action Plus. We consider all of these categories together for all analyses in the HOPE study.

SEN provision: provision different from or additional to that normally available to pupils of the same age[1]

Stages of the national curriculum[10]

The typical educational journey in England is segmented into a variety of blocks called "key stages", during which children are expected to learn a set of subjects. At the end of each key stage, there is an assessment of child's performance.

Early Years Foundation Stage (EYFS): covers ages 3 to 5. At the end of EYFS (final term of *reception* i.e., the year when a child turns 5 years old) children are assessed by class teacher on the basis of classroom observations. Assessed areas currently include communication, physical, personal, social and emotional development, literacy, mathematics, understanding the world, and expressive arts and designs (note that assessment has changed over time).

Key Stage 1: covers Year 1 (age 5 to 6) and Year 2 (age 6 to 7). In Year 1 there is a phonics screening check, whilst in Year 2 children take national tests in English reading and maths, and are assessed in maths, science, and English reading and writing by teachers.

Key Stage 2: covers Year 3 (age 7 to 8) to Year 6 (age 10 to 11). At the end of Key Stage 2, children take national tests in English reading, maths, and grammar, punctuation and spelling, and are assessed by teachers in English writing and science.

Key Stage 3: covers Years 7 (Age 11 to 12) to 9 (age 13 to 14). Year 7 is considered the start of "secondary" school. After 2008, the national curriculum does not require any assessments. Before, 2008 there were Standardised Assessment Tests (SATs).

Key Stage 4: covers educational Years 10 (age 14 to 15) and 11 (age 15 to 16). For most students, this includes being examined using the (International) General Certificate of Secondary Education (GCSE). Special schools provide NCFE qualifications which are highly reputable vocational and work-related courses, designed to accommodate the needs of employers for immediate full-time employment and allow students to progress to higher education to degree level.

For peer review only - http://bmjopen.bmj.com/site/about/guidelines.xhtml

Appendix 3 - Additional Results & Methods

Appendix Table 1 – Overview of number of children entering primary school (Year 1) captured in ECHILD

Academic year	All children in school in Year 1 (recorded in NPD)	All children in school in Year 1 linked to a HES record	All children in school in Year 1 linked to a birth admission in HES
2009/10	574,833	543,135 (94.5%)	449,166 (78.1%)
2010/11	587,163	553,705 (94.3%)	474,295 (80.8%)
2011/12	600,455	565,764 (94.2%)	489,041 (81.4%)
2012/13	620,754	584,215 (94.1%)	498,376 (80.3%)
2013/14	647,299	605,217 (93.5%)	504,161 (77.9%)
2014/15	645,292	601,087 (93.1%)	527,385 (81.7%)
2015/16	659,361	613,953 (93.1%)	556,171 (84.3%)
2016/17	669,197	623,097 (93.1%)	570,284 (85.2%)
total	5,004,354	4,690,173 (93.7%)	4,068,879 (81.3%)

HES=Hospital Episode Statistics, NPD=National Pupil Database

Search terms for literature review for health phenotype definition

("cognitive impairment*" OR "cognitive delay*" OR "learning disabilit*" OR "intellectual disabilit*" OR "*developmental disability* " OR "developmental delay*" OR "special education" OR "special educational needs" OR "special educational need" OR "additional learning need*" OR "special school*" OR "additional learning support") AND ("systematic review" OR "meta-analysis" OR "meta analysis" OR "cohort study" OR "observational study" OR "population based" OR "population-based" OR "register study" OR "registry") AND (child* or adolescen* or pupil* or teeange* or "school student*") AND (congenital or "birth defect*" or chromosomal or "chronic disease" or "chronic condition*" or "long term condition*" or "long term disease" or "life limiting" or "end stage" or palliative or "liver disease" or asthma or cancer or malignan* or diabetes or obes* or encephalpath* or "cystic fibrosis" or "renal disease" or "kidney disease" or "heart disease" or cardiomyopathy or endocarditis or "lung disease" or "liver disease" or epilepsy or epileptic* or seizure* or "sickle cell" or "physical condition" or or illness or "brain injury" or "brain trauma" or cardiovascular or stroke or preterm or "birth weight" or birthweight or neonatal or gestation* or asphyxia or "spinal injury" or hydrocephalus or "nervous system" or autoimmune or eczema or arthritis or psoriasis or infection or medication)

For peer review only

Appendix Table 2 – Roadmap for causal investigations in HOPE, with an exemplar of cleft lip and palate

	**Steps in the design of the study:**	**Exemplar: *Children with cleft lip and palate (CLP) abnormalities***
**1.**	Articulate the scientific question and specify the background knowledge:	*Does special educational needs (SEN) provision improve the health and educational outcomes of children with CLP?*
	a) define the population of interest	Children with CLP identified in HES before age 5, born between 2003 and 2012, who started compulsory education between 2008 and 2018, with linked HES and NPD data
	b) specify the outcomes	• Number of days in contact with an accident and emergency department by Year 6<br>• Number of unplanned school absences by Year 6
	c) specify the intervention ("exposure")	• SEN support vs no recorded SEN provision<br>• SEN support vs EHCP
	d) draw assumptions regarding exposure, outcome and their common causes	*Draw DAG that includes unmeasured variables (as relevant) and identifies a minimum set of confounding variables*
	e) translate the causal question in terms of a contrast of means of potential outcomes*	*What are the benefits for the children who did receive SEN provision?*<br>→ Average treatment effect in the treated (ATT)<br><br>*Would other children with CLP benefit from SEN?*<br>→ Average treatment effect in the non-treated (ATNT)<br><br>*What would be the consequence of a new policy that increases the provision of SEN for all children with CLP? Or for those with more severe CLP?*<br>→ Interventional treatment effect (ITE)
**2.**	Can the question be addressed with the data at hand?	
	a) is the exposure well-defined and available in the data?	We have access to two categories of recorded SEN provision (SEN support and EHCP) and treat each of them to represent delivered support (covering a variety of different interventions)
	b) is the exposure suitable/available for everyone in the population of interest?	Some groups of children appear not to be eligible for an EHCP
	c) is there an issue of selection bias?	Missing values affect some of the confounding variables
**3.**	Causal contrasts	

For peer review only - http://bmjopen.bmj.com/site/about/guidelines.xhtml

	a) For which (sub-)population we wish to address the causal question?	Depending on the question:<br>• Children with CLP who have a record of any SEN provision;<br>• Children with CLP who do not have a record of any SEN provision;<br>• All children with CLP;<br>• Children with more severe CLP;
	b) On which scale?	For both outcomes we will examine:<br>– rate ratios<br>– rate differences
**4.**	Estimation:	
	a) Which estimation approach would target the causal contrast we are interested in?	For the ATT and ATNT:<br>• G-computation;<br>• Inverse probability weighting (IPW) of marginal structural models (MSMs), with alternative approaches to specify the propensity score;<br>• Doubly robust methods;<br>• Difference-in-Differences;<br><br>For the ITE:<br>• G-computation<br>• IPW of MSMs, with alternative approaches to specify the propensity score<br>• Doubly robust methods
	b) Are the assumptions invoked by alternative estimation approaches defensible?	• Unmeasured confounding may be at play;<br>• Parametric models used may be misspecified but robust methods can be used;
**5.**	Interpretation	
	a) Are the results comparable? And why not if not?	If comparable, results are more robust to misspecification and unmeasured confounding
	b) Triangulating results and compare with external evidence	Current evidence on impact is limited

**Potential outcomes are the outcomes that would occur under intervention on the exposure ATNT= Average treatment effect in the non-treated, ATT=Average treatment effect in the treated, CLP= cleft lip and palate, DAG=Directed Acyclic Graph, EHCP=Education, Health and Care Plan, HES=Hospital Episode Statistics, IPW=Inverse Probability Weighting, ITE=Interventional treatment effect, MSM=Marginal Structural Models, NPD=National Pupil Database, SEN=Special Educational Needs*

For peer review only - http://bmjopen.bmj.com/site/about/guidelines.xhtml

References

1. Department for Education. Special educational needs and disability code of practice: 0 to 25 years. Published online 2015. doi:DFE-00205-2013

2. Jay MA, Grath-Lone LM, Gilbert R. Data Resource: the National Pupil Database (NPD). *International Journal of Population Data Science*. 2019;4(1). doi:10.23889/ijpds.v4i1.1101

3. The National Audit Office. Support for pupils with special educational needs and disabilities in England. Published online 2019. Accessed January 4, 2023. https://www.nao.org.uk/wp-content/uploads/2019/09/Support-for-pupils-with-special-education-needs.pdf

4. Jay MA, Gilbert R. Special educational needs, social care and health. *Archives of Disease in Childhood*. 2021;106(1):83-85. doi:10.1136/archdischild-2019-317985

5. Hutchinson, Jo. Identifying pupils with special educational needs and disabilities. Published online 2021. Accessed October 5, 2021. https://epi.org.uk/wp-content/uploads/2021/03/SEND-Indentification_2021-EPI.pdf

6. Department for Education. 01 - Pupils in all schools, by type of SEN provision - including independent schools and general hospital schools - 2016 to 2022. Accessed January 23, 2023. https://explore-education-statistics.service.gov.uk/

7. Department for Education. Special educational needs in England, Academic Year 2021/22. Published June 16, 2022. Accessed January 23, 2023. https://explore-education-statistics.service.gov.uk/find-statistics/special-educational-needs-in-england#dataBlock-678da1e3-97e6-42f1-165e-08da47b0392d-tables

8. Hernan MA, Robins JM. *Causal Inference: What If*. Boca Raton: Chapman & Hall/CRC.; 2020.

9. Cullen MA, Lindsay G, Hastings R, Denne L, Stanford C. *Special Educational Needs in Mainstream Schools: Evidence Review*. Education Endowment Foundation; 2020. Accessed January 4, 2023. https://eric.ed.gov/?id=ED612232

10. Department for Education. The national curriculum. Accessed July 4, 2023. https://www.gov.uk/national-curriculum

For peer review only - http://bmjopen.bmj.com/site/about/guidelines.xhtml

BMJ Open

Evaluation of variation in special educational needs provision and its impact on health and education using administrative records for England: umbrella protocol for a mixed methods research programme

Journal:	*BMJ Open*
Manuscript ID	bmjopen-2023-072531.R3
Article Type:	Protocol
Date Submitted by the Author:	06-Oct-2023

Complete List of Authors:	Zylbersztejn, Ania; UCL Great Ormond Street Institute of Child Health Population Policy and Practice<br>Lewis, Kate; UCL Great Ormond Street Institute of Child Health Population Policy and Practice<br>Nguyen, Vincent; UCL Great Ormond Street Institute of Child Health Population Policy and Practice<br>Matthews, Jacob; University of Cambridge, Department of Psychiatry<br>Winterburn, Isaac; University of Cambridge, Department of Psychiatry<br>Karwatowska, Lucy; UCL Great Ormond Street Institute of Child Health Population Policy and Practice<br>Barnes, Sarah; University of Cambridge, Department of Psychiatry<br>Lilliman, Matthew; UCL Great Ormond Street Institute of Child Health Population Policy and Practice<br>Saxton, Jennifer; University of Cambridge, Department of Psychiatry<br>Stone, Antony; UCL Great Ormond Street Institute of Child Health Population Policy and Practice<br>Boddy, Kate; University of Exeter Medical School, Department of Health and Community Sciences<br>Downs, Johnny; King's College London, Institute of Psychiatry, Psychology and Neuroscience; South London and Maudsley NHS Foundation Trust<br>Logan, Stuart; University of Exeter Medical School, The Peninsula Childhood Disability Research Unit<br>Rahi, Jugnoo; UCL Great Ormond Street Institute of Child Health Population Policy and Practice; UCL Institute of Ophthalmology<br>Black-Hawkins, Kristine; University of Cambridge, Faculty of Education<br>Dearden, Lorraine; UCL Social Research Institute<br>Ford, Tamsin; University of Cambridge, Psychiatry<br>Harron, Katie; UCL Great Ormond Street Institute of Child Health Population Policy and Practice<br>De Stavola, Bianca ; UCL Great Ormond Street Institute of Child Health Population Policy and Practice<br>Gilbert, Ruth; UCL Great Ormond Street Institute of Child Health Population Policy and Practice

**Primary Subject Heading**:	Epidemiology

Secondary Subject Heading:	Public health, Qualitative research, Research methods, Paediatrics
Keywords:	EPIDEMIOLOGY, Health Equity, PUBLIC HEALTH, QUALITATIVE RESEARCH, STATISTICS & RESEARCH METHODS, Health informatics < BIOTECHNOLOGY & BIOINFORMATICS

SCHOLARONE™
Manuscripts

For peer review only - http://bmjopen.bmj.com/site/about/guidelines.xhtml

Evaluation of variation in special educational needs provision and its impact on health and education using administrative records for England: umbrella protocol for a mixed methods research programme

Ania Zylbersztejn[1], Kate Lewis[1], Vincent Nguyen[1], Jacob Matthews[2], Isaac Winterburn[2], Lucy Karwatowska[1], Sarah Barnes[2], Matthew Lilliman[1], Jennifer Saxton[2], Antony Stone[1], Kate Boddy[3], Johnny Downs[4,5], Stuart Logan[7], Jugnoo Rahi[1,8,9,10,11], Kristine Black-Hawkins[12], Lorraine Dearden[13], Tamsin Ford[2], Katie Harron[1], Bianca De Stavola[1], Ruth Gilbert[1]

[1]Population, Policy and Practice Research and Teaching Department, UCL Great Ormond Street Institute of Child Health, London, UK

[2]Department of Psychiatry, University of Cambridge, Cambridge, UK

[3]Department of Health and Community Sciences, University of Exeter Medical School

[4]Institute of Psychiatry, Psychology and Neuroscience, Kings College London, London, UK

[5]South London and Maudsley NHS Foundation Trust, London, UK

[7]The Peninsula Childhood Disability Research Unit, University of Exeter Medical School, Exeter, UK

[8]Great Ormond Street Hospital NHS Foundation Trust London, UK

[9]UCL Institute of Ophthalmology, London, UK

[10]NIHR Moorfields Biomedical Research Centre London, UK

[11]Ulverscroft Vision Research Group UCL London, UK

[12]Faculty of Education, University of Cambridge, Cambridge, UK

[13]UCL Social Research Institute, London, UK

Corresponding author: Dr Ania Zylbersztejn,

Population, Policy and Practice Research and Teaching Department, UCL Great Ormond Street Institute of Child Health, 30 Guilford Street, WC1N 1EH, London, UK

ania.zylbersztejn@ucl.ac.uk

Word count: 4500/4000

For peer review only - http://bmjopen.bmj.com/site/about/guidelines.xhtml

Abstract

Introduction: One-third of children in England have special educational needs (SEN) provision recorded during their school career. The proportion of children with SEN provision varies between schools and demographic groups, which may reflect variation in need, inequitable provision and/or system factors. There is scant evidence on whether SEN provision improves health and education outcomes.

Methods: The HOPE research programme uses administrative data from the Education and Health Insights from Linked Data – ECHILD – which contains data from all state schools, and contacts with NHS hospitals in England, to explore variation in SEN provision and its impact on health and education outcomes. This umbrella protocol sets out analyses across four work packages (WP). WP1 defined a range of "*health phenotypes*", that is health conditions expected to need SEN provision in primary school. Next, we describe health and education outcomes (WP1) and individual, school- and area-level factors affecting variation in SEN provision across different phenotypes (WP2). WP3 assesses the impact of SEN provision on health and education outcomes for specific health phenotypes using a range of causal inference methods to account for confounding factors and possible selection bias. In WP4 we review local policies and synthesise findings from surveys, interviews and focus groups of service users and providers to understand factors associated with variation in and experiences of identification, assessment, and provision for SEN. Triangulation of findings on outcomes, variation and impact of SEN provision for different health phenotypes in ECHILD, with experiences of SEN provision will inform interpretation of findings for policy, practice and families and methods for future evaluation.

Ethics and dissemination: Research ethics committees have approved the use of the ECHILD database and, separately, the survey, interviews and focus groups of young people, parents and service providers. These stakeholders will contribute to the design, interpretation and communication of findings.

For peer review only

Strengths and limitations of this study

Strengths:

- ECHILD database comprises longitudinal histories of all hospital contacts funded by the National Health Service (NHS) and state-funded schooling for 14.7 million children in England, enabling exploration of outcomes for different health phenotypes over time, and by geographic area and sociodemographic characteristics.
- We define phenotypes in health data, which are recorded independently from processes in schools that lead to SEN provision and apply different biostatistical and econometric methods to address potential confounding and selection bias.
- We use lived experience evidence from service users and providers to understand varying processes for identifying children who need and are provided with interventions for SEN together with evidence from analyses of ECHILD to strengthen the robustness of our findings and interpretation.

Limitations:

- The provision of SEN is based on organisational and social factors, as well as additional learning needs of children that are not objectively measured in health or education data before intervention.
- The ECHILD database does not capture NHS healthcare outside acute hospital settings, education support at home or in the non-state funded sector, or information on what (if any) SEN provision was received and whether it was appropriate.

For peer review only

For peer review only - http://bmjopen.bmj.com/site/about/guidelines.xhtml

Introduction

National policies in the UK and in many high-income countries require schools to make adaptations to meet the needs of children who have health, learning or behavioural problems, that impact their ability to learn. These children are referred to collectively as having special educational needs (SEN). Interventions and adjustments in schools for children with SEN are referred to as *SEN provision*, and are intended to improve inclusion and participation in education and support children's health and wellbeing (see Appendix 1 for details of SEN provision in England).[1] Since 2015, approximately one in six children in England are recorded as receiving any SEN provision each year (see Figure 1),[2] and one-third of all children have a record of any SEN provision at least once during their time in education.[3,4]

SEN provision across England is widely regarded as inequitable.[2,4–6] The proportion of pupils with SEN support (more common type of provision, arranged and funded by the schools, see Appendix 1) ranged from 7.3% to 17.1% across local authorities in 2018/19. The proportion with Education, Health and Care Plans (EHCPs, which involve additional, more intensive, and higher cost provision for children whose needs cannot be fully met by SEN support, arranged and partly funded by local authorities[2,7]) ranged from 0.8% to 5.0%.[8] Allocation of SEN provision is associated with a variety of factors. According to a recent report, a key factor determining SEN provision is the school, particularly school's previous rates of SEN provision, academy status and previous school inspection outcomes.[4] Other factors include the proportion of academised primary schools and rates of pupils eligible for free school meals at local authority level-and pupil-level factors such as attainment at school entry (age 5), ethnic group, child's first language and contacts with social care.[4–6] The annual proportion of children with recorded SEN provision has declined over time, from 20% in school year ending in August 2010 to 14% in 2016. This change seems partly related to the Children and Families Act in 2014 and Special Educational Needs and Disability Code of Practice implemented in 2015, and to reduced funding to local authorities from 2010 (Figure 1).[2,9]

Compared to their peers, children with recorded SEN provision have higher rates of chronic physical and mental health conditions and hospitalisations, and have lower self-reported wellbeing.[10–12] Recent evidence reviews found that classroom-based SEN interventions improved children's social, emotional wellbeing and reduced challenging behaviour, and contributed to better mental health outcomes.[13,14] For children with attention deficit hyperactivity disorder (ADHD), systematic reviews of randomised, or quasi randomised controlled trials (RCTs) of interventions similar to SEN provision found improvements in behaviour.[10,15–17] However, there is a lack of RCTs or representative observational comparative studies of the impact of SEN provision, as delivered in routine practice, on health outcomes for a range of health conditions.

Robust evidence that SEN provision improves educational outcomes for pupils with SEN is also scarce.[13] There is moderate evidence that SEN provision in primary schools improves literacy difficulties, socio-emotional development and language and communication.[14] A recent evidence review found a weak but positive impact of inclusive education involving additional support for those with additional learning needs on academic outcomes among pupils without SEN provision.[13] Evidence from population-based observational studies suggests that SEN provision is associated with fewer absences and exclusions among children and young people with neurodisability or mental health conditions.[18]

Given the large proportion of children with SEN provision indicated in school records, the high costs of SEN provision, and static school funding per pupil since 2010,[9] evidence is needed to quantify how SEN provision varies across England and to guide effective intervention to groups of children who are most likely to benefit. The Health Outcomes of young People in Education (HOPE) research programme aims to address these gaps through novel proof-of-concept analyses of the Education and Child Health Insights from Linked Data (ECHILD) database, which links data from all state schools, and contacts with NHS hospitals in England, and mixed methods (surveys, interviews and

For peer review only - http://bmjopen.bmj.com/site/about/guidelines.xhtml

focus groups with families and service providers, and document analyses) to understand experiences of service users and providers. We will assess two central research questions: i) which factors contribute to variation in SEN provision in England; and ii) what is the impact of SEN provision on health and education outcomes? We will address these research questions for a range of health conditions associated with increased need for SEN provision, which we refer to as *health phenotypes*.

This umbrella protocol sets out the research plan for the HOPE programme to address these two core questions. We describe four parallel work packages (WP), and in brief, the component studies contributing to each WP. The conceptual framework and proposed research questions are illustrated in Figure 2. Separate study protocols for each component study will be pre-registered on NIHR Open Research platform (https://openresearch.nihr.ac.uk/) and follow relevant reporting guidelines from the EQUATOR Network (https://www.equator-network.org/, e.g. analyses using ECHILD will be reported using RECORD guidelines for studies using linked administrative data).[19]

In the first work package (WP1), we define a range of *health phenotypes*, that is health conditions captured in hospital records that are expected to need SEN provision in primary school. We explore how health and education outcomes vary for children with different health phenotypes and compared to unaffected peers. In WP2, we describe how child, social and area-level factors affect variation in SEN provision within phenotypes. In WP3, we apply a range of causal inference methods to address confounding factors (informed by WP2) and possible selection bias to assess the impact of SEN provision on outcomes for children with selected health phenotypes (defined in WP1), also considering timing, duration and level of provision. The ECHILD database contains termly records indicating provision for SEN, but no information about whether or when any provision was actually received, its type or quality. WP4 applies mixed methods to understand geographic variation in local policies and the underlying processes of identification, assessment and provision, and how these processes are experienced by families. Triangulation of findings on outcomes (WP1), variation in (WP2) and impact of (WP3) SEN provision for children with different health phenotypes from ECHILD, with findings on local policies and experiences of SEN provision (WP4) will inform findings for policy, practice and families and methods for future evaluation.

The HOPE research programme started in August 2021 and is expected to end in April 2025. The research programme is ongoing and elements of the programme that have already been completed at the time of publication of this protocol are highlighted in the methods and analyses below.

Methods and analysis

Data sources and study measures for WP1-3

ECHILD database

The ECHILD database links routinely collected administrative data on health and education in England. Currently ECHILD includes all children and young people aged 0–24 years in England who were born between 1 September 1995 and 31 August 2021 (approximately 14.7 million individuals).[20] Health and education datasets were linked by NHS England using a multi-step deterministic linkage algorithm, described in detail elsewhere.[21] Linkage rates were high and increased over time (92% of school pupils born in academic year 1990/91 were linked to a hospital record, compared to 99% of pupils born in 2004/05).[21]

Health data consists of Hospital Episode Statistics (HES), a national database that includes dated information on all National Health Service (NHS) acute hospital care and mortality data (see Figure 3 for details of data coverage by academic year).[22] Nearly all children born in England are born in NHS hospitals (97%) but HES excludes births in private hospitals or at home. Children can be followed from their birth admission through all subsequent NHS hospital contacts.[23,24]

For peer review only - http://bmjopen.bmj.com/site/about/guidelines.xhtml

Education records are collated in the National Pupil Database (NPD) and include information on children's registrations in schools, attainment scores at ages 5, 7 11, 16 and 18 (see Study Glossary in Appendix 2 for details), and number of half-day absences and exclusions in each 13-week term. SEN provision is recorded each term (annually prior to 2005/6) for all children in state-funded education (93% of all children) from the academic year starting in September 2001 onwards (see Figure 3).[25] Education data captures the category of recorded SEN provision (SEN support or EHCP) and main reason for SEN provision. Since 2014/15, reasons for SEN include language or communication, moderate or severe learning disability, autism, sensory impairment, physical disability, or social, emotional or mental health needs (see Appendix 1 for details). We are in the process of enhancing ECHILD with school characteristics, such as type of school (e.g.: mainstream or special) or teacher-pupil ratio, from a range of publicly available data.[26–28]

All analyses of ECHILD across WP1-3 will use shared definitions of study population, SEN provision, and outcomes, as described in more detail below. The analyses of the ECHILD database will be a proof of concept, restricted to children attending primary school and three groups of health phenotypes (described in detail below). Analyses of all age groups, and all possible health phenotypes, types of SEN provision, and possible comparisons are beyond the scope of the HOPE programme, but can be informed by our methods. We will publish our methods and code to enable others to reproduce and extend our analyses using ECHILD.

Study cohort & health phenotypes

Our target population is primary school aged children (enrolled in school in Year 1 aged 5/6 and followed to Year 6 aged 10/11), who were born in an NHS hospital in England and had a birth admission recorded in HES data from September 2002 onwards. We follow up all children from birth and use information about risk factors at birth (such as gestational age, birth weight) and health phenotypes recorded in health data before the start of primary school.[23,24] We can then evaluate exposure to SEN provision at the start of primary school and the impact on subsequent health and education outcomes. We analyse three groups of health phenotypes as we hypothesise that the impact of SEN provision on health and education outcomes will vary for children with different health phenotypes.

Not all children in English primary schools have a birth record in HES. Between 1st September 2009 and 31st August 2017 there were 5,004,354 children entering primary school (recorded in school census in Year 1, aged 5-6 years old), of whom 94% had a linked HES record and 80% had a linked birth record (the linkage rate increased over time, see Appendix Table 1 in Appendix 3). As these numbers are large, we can focus on specific or even uncommon phenotypes.

We will use clusters of coded clinical information in the ECHILD database to define *health phenotypes* that represent health conditions associated with learning impairment or need for additional educational support. We have defined three groups of health phenotypes which capture populations with different levels of need for SEN provision:

a) *Neurodisability and other high-risk conditions*

The first group of health phenotypes comprises neurologic conditions or complex systemic health problems reported to be associated with learning impairment or behaviours that require SEN provision. These include neurodisability such as autism or learning disabilities,[29,30] cerebral palsy,[31] or epilepsy.[32] The list of health phenotypes has been derived from an overview of systematic reviews and population-based observational studies (see Appendix 3 for overview of search terms) and discussions with clinical experts and service providers. We developed coding algorithms for these health phenotypes based on combinations of diagnostic and procedure codes, where possible from previously validated code lists. As part of validation, we will compare the cumulative incidence and mortality rates by age for each health phenotype with external population

For peer review only - http://bmjopen.bmj.com/site/about/guidelines.xhtml

studies (for example, from national surveys and disease registries) and changes over time to assess consistency of recorded diagnoses in hospital records and to further refine the phenotyping algorithm. A detailed phenotyping paper is in preparation.

Preliminary findings for children with neurodisability or high-risk conditions include 50 high-risk health phenotypes recorded in hospital records before the age of 5. These account for approximately 5% of all children starting primary school in 2008/9-2018/19, 10% of children with any recorded SEN provision during primary school and 30% of those with an EHCP. Some of the included conditions (such as autism or learning disability) are likely to be under-reported in hospital records.[33]

b) Major congenital anomalies

The second group includes children with major congenital anomalies (MCAs), as children with MCAs are likely to require support from specialist services and have a diagnosis recorded in hospital admissions records, creating a reliable phenotype defined before entry to primary school among children whose need for SEN provision is likely to vary.[34] We are using a code list of ICD-10 diagnoses developed by EUROCAT – a European Congenital Anomaly Registry (https://eu-rd-platform.jrc.ec.europa.eu/eurocat),[35,36] which groups MCAs into 12 body system groups and includes 25 specific subgroups.

Our preliminary findings suggest that children with MCAs recorded in the first year of life account for 3.5% of the primary school population in 2008/9-2018/19, 5.5% of children with any recorded SEN provision during primary school, and 13.6% of those receiving EHCPs in mainstream school or attending a specialist school.

c) Whole population phenotype: gestational age

Finally, we are using week of gestational age at birth to assess the gradient in impact of SEN provision across the whole population of children, stratified according to different levels of underlying need. This approach is supported by the finding that each week of birth before 40 weeks of gestation is associated with reduced school attainment scores and an increased risk of SEN intervention.[11,37,38] Approximately 4.5% of children in primary school in 2008/9-2018/19 were born preterm (at <37 weeks' gestation), accounting for 6.0% of children with any recorded SEN provision during primary school, and 8.4% of those receiving EHCPs.

Health and education outcomes

We focus on outcomes that can be measured in hospital and education data: unplanned (A&E and unplanned admissions) and planned hospital contacts (planned or elective admissions and outpatient appointments), school attainment (as proxy measure for cognitive function), and rates of school absences (see Box 1 for study measure definitions).

Follow up of outcomes will cease before the onset of the COVID-19 pandemic. COVID-19 had a significant impact on the wellbeing of young people.[39] Lockdowns in England affected children's access to school and the frequency of hospital contacts captured in ECHILD data. Planned and unplanned admissions, and outpatient appointments reduced substantially during the COVID-19 pandemic, with the largest reductions in children with indicators of vulnerability (such as preterm birth, a chronic condition, recorded SEN or social care record).[12,40] School attainment measures were not collected during the pandemic to help reduce the burden on educational and care settings. In the HOPE programme, we will therefore limit our analyses to outcomes recorded before the start of COVID-19 pandemic.

For peer review only - http://bmjopen.bmj.com/site/about/guidelines.xhtml

Recorded SEN provision

Schools record information on children identified as needing SEN provision (SEN support or EHCP) in school censuses returned to the Department for Education (DfE). We refer to this recording as *SEN provision* throughout the protocol, although we acknowledge that an indication of "SEN provision" in educational records does not evidence that SEN provision is actually received or whether it is appropriate, as these data are not recorded by schools.[25]

We categorise SEN provision in a descending hierarchy for a specified time period (e.g., during school Year 1 or across all of primary school; details shown in Box 1), separating any enrolment in a special school or alternative provision (where the vast majority of children have recorded SEN provision), an EHCP in mainstream school, SEN support in mainstream school, and no recorded SEN provision. These categories have been selected due to differences in the presumed type of provision, in the criteria for provision (e.g.: formal assessment is required for EHCP but not for SEN support) and substantial differences in associated costs.[2]

SEN provision changed following Government education reforms in 2014/15, when EHCPs gradually replaced Statements of SEN, and SEN support replaced SEN without Statement (which grouped two levels of provision: School Action and School Action Plus). We will report changes in recorded SEN over time and address potential impacts of these changes in the design of analyses.

Box 1: key study measures derived from ECHILD database

Measures derived from health data:

Accident & Emergency (A&E) department contact rate: defined as the number of days with at least one A&E contact, divided by person-time at risk during the study period (e.g.: time from start of Year 1 until the end of Year 6 or death).

Unplanned/planned admission rate: defined as the number of unplanned or planned hospital admissions in NHS-funded hospitals in England, divided by person-time at risk during the study period. Admissions will be classified as planned/unplanned according to the admission method recorded in the first episode of care. Consecutive admissions with re-admission within one day of discharge (for example, hospital transfers) will be treated as part of the same admission. Time spent in hospital during an admission will be taken out of the person-time at risk as once a child is in hospital they cannot be at risk of a new admission.

Outpatient department (OPD) appointments and attendances: defined as the number of days with at least one OPD contact, divided by person-time at risk during the study period (e.g.: time from start of Year 1 until the end of Year 6 or death).

Measures derived from education data:

Absence rate: schools are required to take attendance registers twice a day, for morning and afternoon sessions. Absence rates are defined in line with the definition used by Department for Education as the total number of absent sessions (including authorised and unauthorised absences) divided by the total number of possible sessions during the study period.

Standardised attainment measures: We derive standardised attainment measures using recorded scores from national tests in reading, writing and maths at the end of Year 2 (aged 7, Key Stage 1) and at the end of Year 6 (aged 11, Key Stage 2, see Appendix 2 for details of Key Stages). Standardised test scores are calculated using mean and standard deviation of the test scores of all pupils in a given academic year. We will present the proportion of children not assessed (i.e.: did not have an assessment record) and average score for those assessed by study population.

For peer review only - http://bmjopen.bmj.com/site/about/guidelines.xhtml

School readiness indicators: we use scores from teacher assessments of children's development across multiple areas of learning, carried out in the final term before Year 1 (Early Year Foundation Stage Profile, EYFSP, see Appendix 2 for details). Standardised EYFSP scores are calculated using mean and standard deviation of the EYFSP scores of all pupils in a given academic year. We will present the percentage of children who were not assessed (i.e.: did not have an assessment record), the proportion of children not reaching Good Level of Development (GLD, defined by Department for Education using a subset of EYFPS scores) and average scores for those assessed by study population.

SEN provision: we use 4 categories (which may be merged for some analyses) in the following descending hierarchy for a specified time period (e.g.: Year 1):

1) Enrolment in specialist provision (including special school or alternative),
2) EHCP (including 'Statement of SEN' or 'EHCP') in mainstream school,
3) SEN support (including 'School Action', 'School Action Plus' or 'SEN support') in mainstream school,
4) No SEN provision.

Data sources for WP4

National online survey

Research for WP4 to-date includes three online surveys aimed at (1) children and young people, (2) parents/carers and (3) service providers (health, education and local authority professionals). The surveys document variation in local experiences of identification and assessment of need, and provision of SEN intervention. Detailed information on survey design and findings will be published separately. In brief, the surveys were developed through a scoping review to identify previous questionnaires and co-designed with stakeholder groups of young people, parents/carers and professionals working in education or health with children who have SEN. Each survey underwent three rounds of extensive piloting with the respective advisory groups from the HOPE study. This helped to: i) ensure that the questions and response options matched the lived experience of participants, ii) test accessibility and usability for respective participants, iii) ensure no technical difficulties existed when completing the survey across multiple device types (e.g smartphone, laptop). Data were collected using REDCap and the surveys were disseminated via social media (Twitter, Instagram, and Facebook), and through professional networks (GOV.UK Notify service, Parent and Carer forums, and stakeholder group contacts). These networks were used to maximise the recruitment of all three groups from each of the nine administrative health regions in England.

In total 1,714 participants took part from across England including: 77 young people aged 11-27 years, 772 parents and carers, and 865 service providers (those working in/closely with education settings, the health services, local authorities). Short summaries of the key findings at the time of submission of this protocol from initial analyses are published on the study website (https://dev.psychiatry.cam.ac.uk/hope-study-health-outcomes-for-young-people-throughout-education/) and more detailed papers are in preparation.

Qualitative studies of children, young people, parent/carer and practitioner experience

We are conducting two qualitative studies (including interviews and focus groups) to explore the experiences and beliefs of children, young people, parents/carers and practitioners about SEN

For peer review only - http://bmjopen.bmj.com/site/about/guidelines.xhtml

provision to assist the interpretation of our quantitative analyses using ECHILD. Both qualitative studies recruited from the survey respondents who agreed to recontact in the national survey described above.

a) Children, young people and parents / carers

We conducted semi-structured interviews with children and young people (supported by carer if they wished) and separately with parents / carers. The topic guide covered the identification, assessment and provision of support for their or their child's special educational needs using a time-line approach. 16 interviews with children and young people and 22 with parents / carers were completed between May and June 2023 and will be analysed using a framework approach (see Analysis Plan section for details).[41]

b) Practitioner working in health and education

We will conduct three focus groups of up to 10 practitioners on each of the major areas identified by our stakeholder groups (identification, assessment and provision of SEN intervention), providing a total of 9 focus groups involving up to 90 practitioners working in relation to SEN across health, education, local authority or social services. Each area of focus will have its own topic guide and each series of focus groups will be facilitated by a team of two researchers and a parent observer. The latter will provide their opinion of the discussion in focused debriefing sessions after each focus group. Analysis will follow a framework approach,[41] and parent observers will contribute to the interpretation of our findings at a final joint meeting.

Analysis plan

Work package 1: Describing health and education outcomes

We will carry out separate descriptive studies for each of the three groups of health phenotypes. We will estimate rates of planned and unplanned hospital contacts and educational outcomes during primary school for children with and without each health phenotype. We will use appropriate generalised linear models for each outcome (e.g.: Poisson regression for rates, logistic regression for binary outcomes). Findings from these analyses will indicate whether there are differences in outcomes across subgroups within each health phenotype and compared to their peers, to inform analyses in WP3.

Work package 2: Variation in recorded SEN provision

We will use ECHILD to understand variation in SEN provision for children with different phenotypes. We will examine how proportions of children with recorded SEN provision changed over time for children with different health phenotypes (e.g.: MCAs), and the percentage in their variation that is explained by factors at the individual-, school- and local authority-level using appropriate multi-level regression models. These analyses will determine whether the impact of SEN provision (examined in WP3) can be evaluated using natural policy experiment designs (e.g. due to changes in policy over time) and instrumental variable analysis. These analyses will also generate findings on variation in SEN provision according to child level demographic, social and service use characteristics.

Work package 3: Impact of SEN provision on outcomes

We will use a range of biostatistical and econometric methods to explore the impact of SEN provision on health and educational outcomes across selected health phenotypes, and triangulate findings from analyses using different methods (briefly described below). First, we will examine the

For peer review only - http://bmjopen.bmj.com/site/about/guidelines.xhtml

impact of recorded SEN provision at a given point in time, in the first year of compulsory education (Year 1). Second, we will assess the impact of the duration of SEN provision, appropriately controlling for likely time-varying factors that may be affected by SEN provision (informed by findings from WP2) and additionally influence future SEN provision. We anticipate separate studies focussing on specific health phenotypes that represent conditions that are relatively similar in their need for SEN and well characterised in health data. For example, cleft lip and palate has been selected from the MCA phenotypic group as it is reliably denoted by diagnostic and operation codes. We will select a well-defined phenotype within the neurodisability group and we also plan to compare exposure to SEN provision for children within defined strata of gestational age at birth across the whole population.

For all causal analyses, we will use the target trial emulation (TTE) framework to guide the creation of study cohorts that correspond to the specific phenotypes and exposure levels (categories of SEN provision) of interest.[42,43] TTE consists of first designing an ideal pragmatic trial that would address the question of interest, and then emulating it as closely as possible using observational data. The advantage of this approach is the avoidance of biases in the design stage, e.g. immortal time bias and prevalent-case bias, that have affected real-world-data studies in the past.[42] Directed Acyclic Graphs (DAGs) will be used to draw our assumptions about the causal structures influencing what we are studying and to identify relevant confounding variables.[44] An outline of the components of our causal investigations is given in Appendix Table 2 (Appendix 3) where these steps are illustrated using the exemplar of one MCA phenotypes: children born with cleft lip and palate. The study protocol for this study has been registered as a preprint.[45]

As ECHILD comprises observational data, a major challenge for each of our causal investigations will be how to address the bias introduced by confounding by indication, as well as possible selection bias due to incomplete linkage across health and education databases. We will contrast estimates of the impact of SEN provision on health and educational outcomes using a variety of complementary methods that rely on measuring all of the confounders, such as regression adjustment, g-computation, inverse probability weighting of marginal structural models (with different approaches to modelling the propensity score), or econometric methods that try to deal with unmeasured confounding by exploiting natural experiments (such as differences-in-differences or interrupted time series), or instrumental variables.

Work Package 4: Understanding policy variation and lived experiences of SEN provision

We will conduct an overarching synthesis of the findings from the national survey, parent and child / young person interviews and focus groups with practitioners, in relation to the quantitative findings from WP1-3. The planning of this work draws from previous similar triangulation of quantitative and qualitative systematic reviews.[17] We will first create a matrix to demonstrate where these different data sources provide similar or conflicting signals. We will then work inductively from the surveys, focus group and interview findings to generate hypotheses about contextual elements that may influence the recording of data in ECHILD, or health outcomes of SEN provision. This approach draws from “complexity theory” which assumes that any psychosocial intervention must be considered in terms of the larger environment in which it is located.[46] We will also work deductively from the results of analyses in WP1-3 to explore potential relationships between SEN provision and health outcomes, as well as exploring what WP4 findings suggest about factors associated with health outcomes in SEN provision. The aim of this approach is to clarify potential explanations for the findings of WP1-3 and to inform future work in this area. Analysis under the two approaches described above will proceed iteratively and in parallel.

To understand local variation in SEN provision we reviewed publicly available documents on the support available for local children with SEN, referred to as “local offer”. We are assessing the

For peer review only - http://bmjopen.bmj.com/site/about/guidelines.xhtml

quality of available information against 51 criteria outlined within the SEND Code of Practice to determine to what extent Local Authorities in England are providing clear, comprehensive, accessible and up-to-date information about available SEN provision and how to access it. Second, we are examining reports from all of the local area SEND inspections published by the Office for Standards in Education, Children's Services and Skills (OFSTED) over the past seven years to examine how effectively local authorities fulfil responsibilities for children and young people with SEN. By collating these documents and assessing their commonalities and differences, we aim to gain an understanding of variation in good and bad practice in SEN provision.

Patient and public involvement

The HOPE study was developed in response to consultations about the need for the ECHILD database with parents and charities supporting children with chronic health conditions and their families.[47,48] We have established three stakeholder groups: young people, parents / carers and professionals working in education or health with children who have SEN. We are collaborating with staff in schools to enable young people with additional learning needs or disabilities to contribute to these advisory groups. In addition, we are iteratively presenting our study plans and preliminary results to parents/carers and young people through research advisory groups at UCL and University of Exeter. These consultations are contributing evidence to all four work packages and dissemination of the research. The HOPE Study Steering Committee includes two parents of children with disability and will adhere to NIHR requirements for payment for time and expenses of lay contributors.

Ethics

Existing research ethics approval has been granted for analyses of the ECHILD database for the purposes set out in the HOPE study (20/EE/0180). Data access is also controlled by agreements with NHS Digital and the DfE. The data contain no identifiers or sensitive dates and data can only be used within the Office for National Statistics Secure Research Environment by approved researchers, with strict statistical disclosure controls of all outputs of analyses (e.g. tables or figures). Details are published here in our privacy notice (https://www.ucl.ac.uk/child-health/research/population-policy-and-practice-research-and-teaching-department/cenb-clinical-4#).

Separate ethics approval has been approved for the mixed-methods research (national survey, interviews and focus groups) involving service users (young people and parents) and service providers (PRE:2021.058). Parents consented for their own involvement and also for their child if under the age of 16. Young people aged 16 or over consented and younger children were asked for assent to their participation using a similar process.

Dissemination

We will present preliminary findings to diverse audiences (academics, analysts at DfE and Department of Health and Social Care, and our stakeholder groups as well as other groups of service users and providers) through seminars, question and answer sessions, workshops and consultations during the study. We will incorporate feedback into final outputs, which will include peer reviewed journal articles, the final study report to funder, and short briefing reports and infographics for non-academics published on the study website.

We will publish our methods and code to enable others to reproduce and extend our analyses using ECHILD. ECHILD can be accessed by accredited researchers through application via the ECHILD team (www.ucl.ac.uk/child-health/echild) and the Research Accreditation Panel. Meta-data and code relating to the HOPE study will be signposted on the study website and made available in the ONS secure environment and in code repository (including on ECHILD GitHub page: https://github.com/UCL-ECHILD). We will hold workshops to promote wider use of findings from the HOPE study for causal analyses of education interventions on health. Examples from the HOPE study will be incorporated into short courses on causal methods and on how to use the ECHILD database.

For peer review only - http://bmjopen.bmj.com/site/about/guidelines.xhtml

The HOPE study aims to build the evidence base for fairer and more effective SEN provision and, by informing national and local policy and the public and changing practice, to improve health and education outcomes of children with SEN.

For peer review only

For peer review only - http://bmjopen.bmj.com/site/about/guidelines.xhtml

Figure captions

Figure 1 – Percentage of total children with recorded SEN provision per school year, January 2003 to 2022 (based on DfE statistics)[8,49,50]

Legend:

DfE: Department for Education; EHCPs: education, health and care plans; SEN: special educational needs; SEND: special educational needs or disability;

Figure 2 – Conceptual framework bringing together research questions (RQ) to be addressed by component studies of the HOPE research programme

Legend: ECHILD: Education and Child Health Insights from Linked Data; EHCP: education, health and care plan; SEN: special educational needs;

Figure 3 – Coverage of ECHILD datasets, by academic year with refreshes

Legend:
CiN: Child in Need Census, CLA: Children Looked After Return, HES: ECHILD: Education and Health Insights from Linked Data, Hospital Episode Statistics, NPD: National Pupil Database, ONS: Office for National Statistics.

a Partial coverage of an academic year as NPD social care data and HES data is collated by financial year (1st April to 31st March). b Partial coverage as HES Accident and Emergency data was experimental and did not have full national coverage. c Partial coverage as HES outpatient data was experimental and did not have full national coverage. d: Partial coverage of an academic year as ONS mortality data was first linked to HES in January 1998. e: The Pupil Referral Unit Census was subsumed in the School Census Pupil Level from 2013/14. f: The Early Years Census included 3- and 4-year olds between 2007/08 and 2012/13. From 2013/14, it includes 2-4 year olds. g: not collected to help reduce the burden on educational and care settings during the coronavirus (COVID-19) pandemic. h: Partial coverage as between the 2002/3 and 2005/6 academic years, data only on a 10% sample of children. i: to be included, but not available yet. j: Key Stage 3 assessments ceased after 2012/13. k: data not provided with standard institutional identifiers in 2019/20 – 2020/21, as evaluation of individual institutional performance is not permitted.

For peer review only

Footnotes

Abbreviations

A&E: accident and emergency department

ADHD: attention deficit hyperactivity disorder

ASD: autistic spectrum disorders

ATNT: Average treatment effect in the non-treated

ATT: Average treatment effect in the treated

CLP: cleft lip and palate

DAG: Directed Acyclic Graph

DfE: Department for Education

ECHILD: Education and Child Health Insights from Linked Data

EHCP: education, health and care plan

HES: Hospital Episode Statistics

IPW: Inverse Probability Weighting

ITE: Interventional treatment effect

MCA: Major Congenital Anomaly

MSM: Marginal Structural Models

NHS: National Health Service

NPD: National Pupil Database

OFSTED: the Office for Standards in Education, Children's Services and Skills

RCT: randomised controlled trial

SEN: special educational needs

TTE: target trial emulation

Author contributions

RG is the principal investigator of the HOPE research programme and KH, TF, LD and BDS lead each of the four work packages, KBH, JD, SL, JR are co-investigators of the programme. AZ and RG produced the first draft of this manuscript, AZ, KL and ML created figures for the manuscript. AZ, KL, VN, AS, ML are responsible for preparing ECHILD data for analyses. JM, IW, SB, JS, KBH and TF are responsible for conceptualisation and implementation of mixed methods studies. BDS, LD, KL, VN, and LK are responsible for design and implementation of quantitative causal inference analyses using ECHILD database. AZ, KH, RG, KL are responsible for design and implementation of descriptive analyses using ECHILD database, with clinical input from JD, SL, JR on defining study populations. JS, JM, IW, KB, KBH, TF, RG, KH, AZ, VN were involved in public engagement activities to inform the study. All authors edited the manuscript. All authors have contributed to and are responsible for the final design of the study. All authors read and approved the final manuscript.

For peer review only - http://bmjopen.bmj.com/site/about/guidelines.xhtml
For peer review only

Acknowledgments

We would like to acknowledge the contribution of members of the HOPE study steering committee. We gratefully acknowledge all children and families whose de-identified data are used in this research.

We are grateful to the Office for National Statistics (ONS) for providing the trusted research environment for the ECHILD Database. ONS agrees that the figures and descriptions of results in the attached document may be published. This does not imply ONS' acceptance of the validity of the methods used to obtain these figures, or of any analysis of the results.

The ECHILD Database uses data from the Department for Education (DfE). The DfE does not accept responsibility for any inferences or conclusions derived by the authors. This work also uses data provided by patients and collected by the National Health Service as part of their care and support. Source data can also be accessed by researchers by applying to NHS Digital. This work was produced using statistical data accessed via the ONS Secure Research Service. The use of this data in this work does not imply the endorsement of the ONS in relation to the interpretation or analysis of the statistical data. This work uses research datasets which may not exactly reproduce National Statistics aggregates.

Competing Interests

We declare no competing interests.

Funding

This study/project is funded by the National Institute for Health Research (NIHR) under its Programme Grants for Applied Research Programme (Grant Reference number NIHR202025). The views expressed are those of the author(s) and not necessarily those of the NIHR or the Department of Health and Social Care.

For peer review only

References

1 Department for Education. Special educational needs and disability code of practice: 0 to 25 years. Published Online First: 2015. doi: DFE-00205-2013

2 The National Audit Office. Support for pupils with special educational needs and disabilities in England. Published Online First: 2019.

3 Jay MA, Gilbert R. Special educational needs, social care and health. *Arch Dis Child*. 2021;106:83–5.

4 Hutchinson, Jo. Identifying pupils with special educational needs and disabilities. Published Online First: 2021.

5 Roman-Urrestarazu A, van Kessel R, Allison C, *et al.* Association of Race/Ethnicity and Social Disadvantage With Autism Prevalence in 7 Million School Children in England. *JAMA Pediatr*. 2021;175:e210054.

6 Strand S, Lindorff A. Ethnic disproportionality in the identification of Special Educational Needs (SEN) in England: Extent, causes and consequences.

7 Department for Education. Special educational needs in England, Academic Year 2021/22. 2022. https://explore-education-statistics.service.gov.uk/find-statistics/special-educational-needs-in-england#dataBlock-678da1e3-97e6-42f1-165e-08da47b0392d-tables (accessed 23 January 2023)

8 Department for Education. 01 - Pupils in all schools, by type of SEN provision - including independent schools and general hospital schools - 2016 to 2022. https://explore-education-statistics.service.gov.uk/ (accessed 23 January 2023)

9 School funding statistics, Financial year 2022-23. https://explore-education-statistics.service.gov.uk/find-statistics/school-funding-statistics (accessed 16 June 2023)

10 Barnes M, Harrison E. The wellbeing of secondary school pupils with special educational needs: Reserach report. *Dep Educ*. Published Online First: 2017.

11 Libuy N, Gilbert R, Mc Grath-Lone L, *et al.* Gestational age at birth, chronic conditions and school outcomes: a population-based data linkage study of children born in England. *Int J Epidemiol*. 2022;dyac105.

12 Mc Grath-Lone L, Etoori D, Gilbert R, *et al.* Changes in adolescents' planned hospital care during the COVID-19 pandemic: analysis of linked administrative data. *Arch Dis Child*. 2022;107:e29–e29.

13 Cullen MA, Lindsay G, Hastings R, *et al.* Special Educational Needs in Mainstream Schools: Evidence Review. Education Endowment Foundation 2020. https://eric.ed.gov/?id=ED612232 (accessed 4 January 2023)

14 Carroll J, Bradley L, Hannant P, *et al.* SEN support: A rapid evidence assessment. *Dep Educ*. 2017.

For peer review only - http://bmjopen.bmj.com/site/about/guidelines.xhtml

15 Moore DA, Russell AE, Matthews J, *et al.* School-based interventions for attention-deficit/hyperactivity disorder: A systematic review with multiple synthesis methods. *Rev Educ*. 2018;6:209–63.

16 Moore D, Benham-Clarke S, Kenchington R, *et al.* Improving Behaviour in Schools: Evidence Review. Education Endowment Foundation 2019. https://eric.ed.gov/?id=ED612210 (accessed 5 January 2023)

17 Richardson M, Moore DA, Gwernan-Jones R, *et al.* Non-pharmacological interventions for attention-deficit/hyperactivity disorder (ADHD) delivered in school settings: systematic reviews of quantitative and qualitative research. *Health Technol Assess Winch Engl*. 2015;19:1–470.

18 John A, Friedmann Y, DelPozo-Banos M, *et al.* Association of school absence and exclusion with recorded neurodevelopmental disorders, mental disorders, or self-harm: a nationwide, retrospective, electronic cohort study of children and young people in Wales, UK. *Lancet Psychiatry*. 2022;9:23–34.

19 Benchimol EI, Smeeth L, Guttmann A, *et al.* The REporting of studies Conducted using Observational Routinely-collected health Data (RECORD) Statement. *PLOS Med*. 2015;12:e1001885.

20 Mc Grath-Lone L, Libuy N, Harron K, *et al.* Data Resource Profile: The Education and Child Health Insights from Linked Data (ECHILD) Database. *Int J Epidemiol*. 2022;51:17–17f.

21 Libuy N, Harron K, Gilbert R, *et al.* Linking education and hospital data in England: linkage process and quality. *Int J Popul Data Sci*. 2021;6. doi: 10.23889/ijpds.v6i1.1671

22 Herbert A, Wijlaars L, Zylbersztejn A, *et al.* Data Resource Profile: Hospital Episode Statistics Admitted Patient Care (HES APC). *Int J Epidemiol*. 2017;46:1093–1093i.

23 Zylbersztejn A, Gilbert R, Hardelid P. Developing a national birth cohort for child health research using a hospital admissions database in England: The impact of changes to data collection practices. *PLOS ONE*. 2020;15:e0243843.

24 Harron K, Gilbert R, Cromwell D, *et al.* Linking Data for Mothers and Babies in De-Identified Electronic Health Data. *PLOS ONE*. 2016;11:e0164667.

25 Jay MA, Grath-Lone LM, Gilbert R. Data Resource: the National Pupil Database (NPD). *Int J Popul Data Sci*. 2019;4. doi: 10.23889/ijpds.v4i1.1101

26 Department for Education. Get Information about Schools (GIAS). https://www.get-information-schools.service.gov.uk/ (accessed 5 January 2023)

27 Department for Education. Statistics: school workforce. GOV.UK. 2023. https://www.gov.uk/government/collections/statistics-school-workforce (accessed 2 October 2023)

28 Department for Education. School and college performance data in England. 2022. https://www.find-school-performance-data.service.gov.uk/ (accessed 2 October 2023)

29 Fleming M, Salim EE, Mackay DF, *et al.* Neurodevelopmental multimorbidity and educational outcomes of Scottish schoolchildren: A population-based record linkage cohort study. *PLoS Med*. 2020;17:e1003290.

For peer review only - http://bmjopen.bmj.com/site/about/guidelines.xhtml

30 Fleming M, Fitton CA, Steiner MFC, *et al.* Educational and Health Outcomes of Children Treated for Attention-Deficit/Hyperactivity Disorder. *JAMA Pediatr*. 2017;171:e170691.

31 Reid SM, Meehan EM, Arnup SJ, *et al.* Intellectual disability in cerebral palsy: a population-based retrospective study. *Dev Med Child Neurol*. 2018;60:687–94.

32 Fleming M, Fitton CA, Steiner MFC, *et al.* Educational and health outcomes of children and adolescents receiving antiepileptic medication: Scotland-wide record linkage study of 766 244 schoolchildren. *BMC Public Health*. 2019;19:595.

33 Zylbersztejn A, Stilwell PA, Zhu H, *et al.* Trends in hospital admissions during transition from paediatric to adult services for young people with learning disabilities or autism: population-based cohort study. *Lancet Reg Health – Eur*. 2023;24. doi: 10.1016/j.lanepe.2022.100531

34 Peppa M. *The safety of influenza vaccination in pregnancy: Examining major congenital malformations as potential adverse outcomes using UK electronic health records*. 2020. https://doi.org/10.17037/PUBS.04658569

35 Kinsner-Ovaskainen A, Lanzoni M, Garne E, *et al.* A sustainable solution for the activities of the European network for surveillance of congenital anomalies: EUROCAT as part of the EU Platform on Rare Diseases Registration. *Eur J Med Genet*. 2018;61:513–7.

36 Boyd PA, Haeusler M, Barisic I, *et al.* Paper 1: The EUROCAT network—organization and processes†. *Birt Defects Res A Clin Mol Teratol*. 2011;91:S2–15.

37 Burger RJ, Roseboom TJ, Ganzevoort W, *et al.* Gestational age and socio demographic factors associated with school performance at the age of 12 years, a population based study. *Paediatr Perinat Epidemiol*. ;n/a. doi: 10.1111/ppe.12990

38 MacKay DF, Smith GCS, Dobbie R, *et al.* Gestational Age at Delivery and Special Educational Need: Retrospective Cohort Study of 407,503 Schoolchildren. *PLOS Med*. 2010;7:e1000289.

39 Office for Health Improvement and Disparities. 4. Children and young people. *COVID-19 mental health and wellbeing surveillance: report*. 2022. https://www.gov.uk/government/publications/covid-19-mental-health-and-wellbeing-surveillance-report/7-children-and-young-people (accessed 1 February 2023)

40 Etoori D, Harron KL, Mc Grath-Lone L, *et al.* Reductions in hospital care among clinically vulnerable children aged 0–4 years during the COVID-19 pandemic. *Arch Dis Child*. 2022;107:e31–e31.

41 Gale NK, Heath G, Cameron E, *et al.* Using the framework method for the analysis of qualitative data in multi-disciplinary health research. *BMC Med Res Methodol*. 2013;13:117.

42 Hernán MA, Robins JM. Using Big Data to Emulate a Target Trial When a Randomized Trial Is Not Available. *Am J Epidemiol*. 2016;183:758–64.

43 Hernán MA, Wang W, Leaf DE. Target Trial Emulation: A Framework for Causal Inference From Observational Data. *JAMA*. 2022;328:2446–7.

44 Greenland S, Pearl J, Robins JM. Causal Diagrams for Epidemiologic Research. *Epidemiology*. 1999;10:37.

For peer review only

For peer review only - http://bmjopen.bmj.com/site/about/guidelines.xhtml

45 Nguyen VG, Zylbergsztejn A, Harron K, *et al.* SEN support from the start of school and its impact on unplanned hospital utilisation in children with cleft lip and palate: a demonstration target trial emulation protocol using ECHILD. 2022;2022.04.01.22273280. https://doi.org/10.1101/2022.04.01.22273280

46 Montuori A. The Complexity of Transdisciplinary Literature Reviews. *Complicity Int J Complex Educ*. 2013;10. doi: 10.29173/cmplct20399

47 UCL. The ECHILD project website. 2020. https://www.ucl.ac.uk/child-health/research/population-policy-and-practice-research-and-teaching-department/cenb-clinical-20 (accessed 19 January 2023)

48 Waind E, Mc Grath-Lone L. The Education & Child Health Insights from Linked Data (ECHILD) Database Key messages from stakeholders. *ADR UK*. Published Online First: 2021.

49 Department for Education. Special educational needs in England: January 2015. GOV.UK. 2015. https://www.gov.uk/government/statistics/special-educational-needs-in-england-january-2015 (accessed 21 September 2023)

50 Ofsted. *The special educational needs and disability review. A statement is not enough*. 2010. https://www.gov.uk/government/publications/special-educational-needs-and-disability-review

For peer review only

For peer review only - http://bmjopen.bmj.com/site/about/guidelines.xhtml

Figure 1 – Percentage of total children with recorded SEN provision per school year, January 2003 to 2022 (based on DfE statistics)[8,45,46]

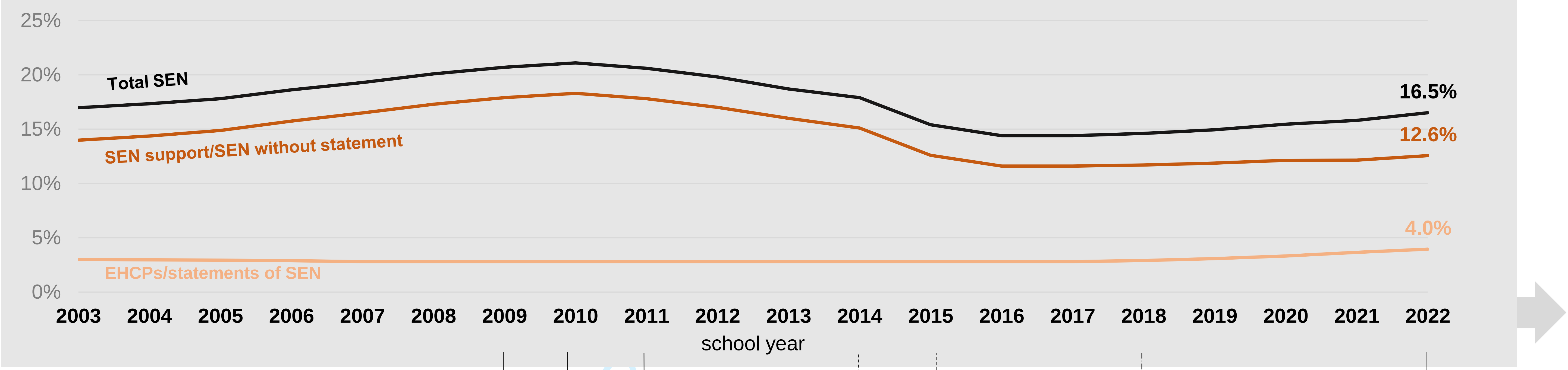

The Special Educational Needs and Disability Act 2001: legislation on rights of disabled and special educational needs (SEN) children in compulsory and post-16 education, training and other student services

Special Educational Needs Code of Practice: statutory guidance for Local Authorities, maintained schools, early years settings and others on carrying out their statutory duties to identify, assess and make provision for children's special educational needs (SEN); effective from **1 January 2002**

2009: Lamb enquiry
UK Government commissioned enquiry focusing on improving parental confidence in the SEN system

2010: Special education needs and disability review: a statement is not enough
The Office for Standards in Education, Children's Services and Skills (Ofsted) was commissioned by UK Government to evaluate how well the legislative framework serves children with SEN. Review identified wide variation in the identification of SEN and provision across schools and local authorities and argued that there is an over identification of SEN in lieu of good teaching.

2010: Salt review
Independent review of teaching for children with severe/profound and multiple learning disabilities

2011: Support and Aspiration: A new approach to SEND

Consultation and subsequent green paper (2012). Confirmed a focus on early identification and a better experience for parents with less 'fighting for' support, and better coordination between health, social care and education services.

2014: Children and Families Act

Legislation on rights of children, families, and people with special educational needs or disabilities

2015: SEND Code of Practice: 0 to 25 years

statutory guidance for organisations working with young people with SEND. Introduced Education, Health and Care Plans (EHCPs) instead of Statements of SEN.

2018: Deadline for Local authorities to **replace statements of SEN with EHCPs**

2022: SEND review: right support, right place, right time

Green paper proposing the establishment of "a new national SEND and alternative provision system setting nationally consistent standards for how needs are identified and met at every stage of a child's journey across education, health and care"

DfE: Department for Education; EHCPs: education, health and care plans; SEN: special educational needs; SEND: special educational needs or disability;

Figure 2 – Conceptual framework bringing together research questions to be addressed by each work package (WP) of the HOPE research programme

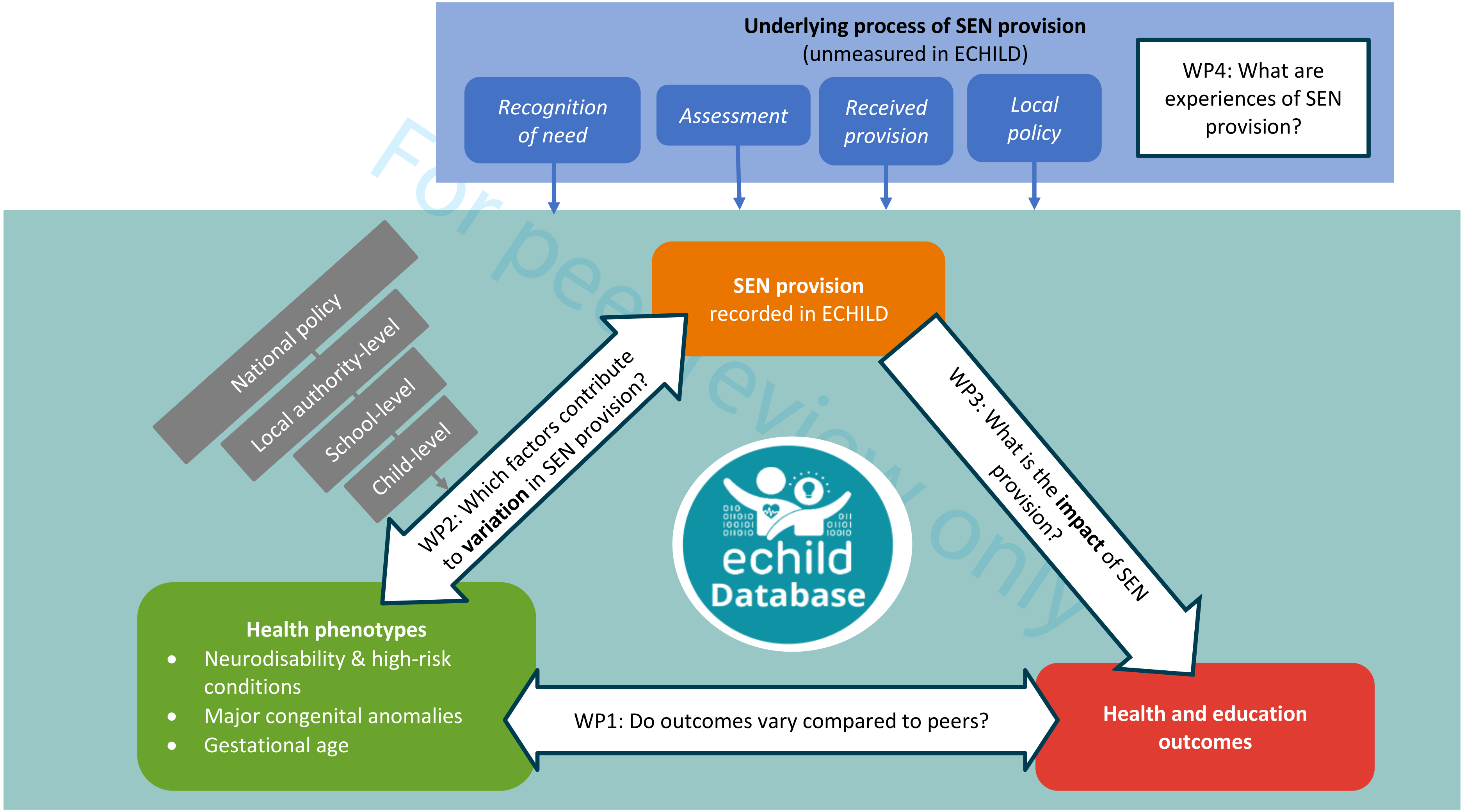

ECHILD: Education and Child Health Insights from Linked Data; EHCP: education, health and care plan; SEN: special educational needs;

Figure 3 – Coverage of ECHILD datasets, by academic year with refreshes

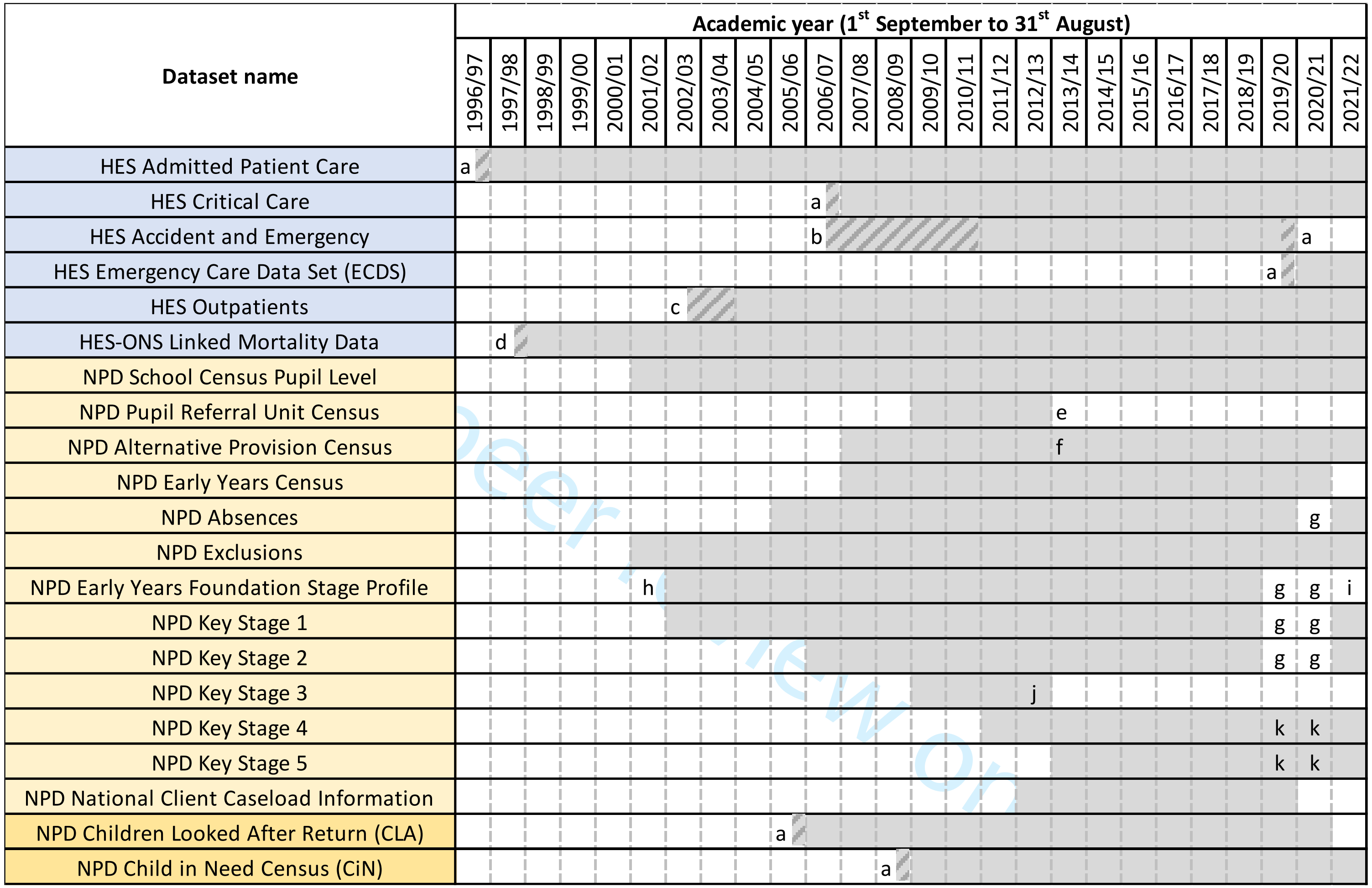

CiN: Child in Need Census, CLA: Children Looked After Return, HES: ECHILD: Education and Health Insights from Linked Data, Hospital Episode Statistics, NPD: National Pupil Database, ONS: Office for National Statistics.

a Partial coverage of an academic year as NPD social care data and HES data is collated by financial year (1st April to 31st March). b Partial coverage as HES Accident and Emergency data was experimental and did not have full national coverage. c Partial coverage as HES outpatient data was experimental and did not have full national coverage. d: Partial coverage of an academic year as ONS mortality data was first linked to HES in January 1998. e: The Pupil Referral Unit Census was subsumed in the School Census Pupil Level from 2013/14. f: The Early Years Census included 3- and 4-year olds between 2007/08 and 2012/13. From 2013/14, it includes 2-4 year olds. g: not collected to help reduce the burden on educational and care settings during the coronavirus (COVID-19) pandemic. h: Partial coverage as between the 2002/3 and 2005/6 academic years, data only on a 10% sample of children. i: to be included, but not available yet. j: Key Stage 3 assessments ceased after 2012/13. k: data not provided with standard institutional identifiers in 2019/20 – 2020/21, as evaluation of individual institutional performance is not permitted.

Appendix 1: Overview of Special Educational Needs provision in England

National policies in the UK and in many high-income countries require schools to make adaptations to meet the needs of children who have health, learning or behavioural problems, that impact their ability to learn; these children are referred to collectively as having special educational needs (SEN). In England, children with SEN "*have a significantly greater difficulty in learning than the majority of others of the same age, or have a disability which prevents them from making use of facilities generally provided by mainstream schools*" (see the study glossary in Appendix 2 below).[1]

There are four broad areas of SEN that state-funded schools are required to support: communication and social interactions, cognition and learning, social, emotional and mental health and sensory or physical disabilities (see Box 1). Interventions and adjustments in schools for children with SEN, referred to as *SEN provision*, are intended to improve inclusion and participation in education and support children's health and wellbeing.[1] Approximately 7% of children in England attend private schools each year,[2] which do not have the same legal obligations regarding SEN identification and provision.

*Box 1 – Broad areas of special educational needs (SEN) that schools should plan for, with sub-categories set out by the Special Educational Needs and Disability code of practice in England[1] (at the time of publication)**

Communication and social interactions
- Speech, language and communication needs
- Autistic Spectrum Disorders

Cognition and learning:
- Moderate learning difficulties
- Severe learning difficulties
- Profound and multiple learning difficulties
- Specific learning difficulties (that is learning difficulties affecting one or more specific aspects of learning, such as dyslexia, dyscalculia or dyspraxia)

Social, emotional and mental health*
- Examples could be attention-deficit/hyperactivity disorder, behavioural difficulties, anxiety, depression, eating disorders

Sensory and/or physical disabilities:
- Vision impairment
- Hearing impairment
- Multi-sensory impairment
- Physical disability requiring additional ongoing support and/or equipment to access all the opportunities available to their peers

**Note that the sub-categories changed in 2014/15 following reforms to SEN system: "Social, emotional and mental health difficulties" were introduced in 2014/15, while "Behaviour, Emotional & Social Difficulties" were removed.*

Since 2015, approximately one in six children in England are recorded by schools as receiving any SEN provision each year,[3] and one-third of all children have a record of any SEN provision at least once during their time in education.[4,5] There are two categories of SEN provision offered in England,

For peer review only - http://bmjopen.bmj.com/site/about/guidelines.xhtml

SEN support and *Education, Health and Care Plans (EHCPs)*. These categories were introduced following Government education reforms in 2014/15, replacing the older categories of *School Action/School Action Plus* (together referred to as *SEN without Statement*) and *Statements of SEN*, respectively (see study glossary in Appendix 2 for details).

The majority of pupils with any recorded SEN provision receive *SEN support*. SEN support is arranged and funded by the schools and can include short-term interventions such as speech or language therapy or extra support for reading. The first assessment for SEN support is usually carried out by the school's teachers, Special Educational Needs Coordinator (SENCo), or after class teachers, who seek to identify children making less than expected educational progress or with additional social needs relative to their peers. In 2018/19 (the last academic year before the COVID-19 pandemic), 11.9% of pupils had SEN support recorded, with the vast majority provided in mainstream schools.[6]

A smaller proportion of children receive an EHCP, which involves additional and more intensive provision arranged and partly funded by local authorities for children whose needs cannot be fully met by SEN support.[3,7] Support may range from extra help by a part-time teaching assistant to full-time care by multiple staff in a special school.[1] An assessment for an EHCP can be requested by parents, schools or health or social care professionals. The assessment is carried out by the local authority, who are required to fill in a legal document setting out the special measures to be provided to meet the child's needs across education, health and social care.[3,7] In 2018/19, 3.1% of pupils had a record of an EHCP, half of whom were enrolled in a special school (1.6% of all children).[6]

Appendix 2: HOPE study glossary

Confounding: The bias caused by shared causes of exposure and outcome.[8]

Confounder: A variable that can be used to adjust for confounding.[8]

Disability: under the Equality Act 2010 a disability is "a physical or mental impairment which has a long-term and substantial adverse effect on their ability to carry out normal day-to-day activities". Not all children with SEN have a disability, and not all disabled children have SEN, but there is significant overlap.[1,9]

Education, health and care plan, EHCP (known as SEN Statement prior to education reform in 2014/15): more intense provision arranged by local authorities, involving a legal document setting special measures provided by local authorities to meet a child's needs across education, health and social care.[1]

Health phenotypes: health conditions which can be indicated in administrative health records using diagnostic data (such as recorded diagnoses or procedures). In HOPE study we focus on health conditions associated with higher need for additional support for SEN than for their peers

Recorded SEN provision: Schools record information on children identified as needing SEN provision in school censuses returned to the Department for Education (DfE). Recorded SEN indicates identification of a child's primary need using fixed categories (see Box 1) and level of appropriate intervention (either SEN support or EHCP).[2] However, there is no centrally collated data on when and what type of intervention was received. Note that for simplicity we refer to *recorded SEN provision* as *SEN provision* in the protocol, although recorded SEN provision does not evidence that SEN provision is actually received or whether it is appropriate.[2]

Reasonable adjustments: schools have a duty to support pupils with medical conditions and make reasonable adjustments for children with disabilities, including the provision of auxiliary aids and services for disabled children. Not all children with disabilities have SEN, therefore reasonable adjustments are not considered SEN intervention.[1]

For peer review only - http://bmjopen.bmj.com/site/about/guidelines.xhtml

Special educational needs, SEN: a child has SEN if they have a health, learning or behavioural problems, that impact their ability to learn and require for special educational provision to be made for him or her.[1] Disability included in definition of SEN is a disability that prevents the child from using facilities generally provided for their peers in mainstream settings.[1]

Special educational needs or disability (SEND): Children with disabilities do not inevitably have SEN, but sometimes the term SEND is used to include children whose disabilities do impact their ability to access the mainstream school curriculum. The term SEND is also used by government departments in England to encompass all children with SEN, disabilities, or both.

SEN support (known as School Action and School Action Plus prior to education reform in 2014/15): SEN provisions arranged and funded by the school and provided almost entirely in mainstream schools. Prior to education reform in 2014/15, children could receive support as part of School Action or more intense support as part of School Action Plus. We consider all of these categories together for all analyses in the HOPE study.

SEN provision: provision different from or additional to that normally available to pupils of the same age[1]

Stages of the national curriculum[10]

The typical educational journey in England is segmented into a variety of blocks called "key stages", during which children are expected to learn a set of subjects. At the end of each key stage, there is an assessment of child's performance.

Early Years Foundation Stage (EYFS): covers ages 3 to 5. At the end of EYFS (final term of *reception* i.e., the year when a child turns 5 years old) children are assessed by class teacher on the basis of classroom observations. Assessed areas currently include communication, physical, personal, social and emotional development, literacy, mathematics, understanding the world, and expressive arts and designs (note that assessment has changed over time).

Key Stage 1: covers Year 1 (age 5 to 6) and Year 2 (age 6 to 7). In Year 1 there is a phonics screening check, whilst in Year 2 children take national tests in English reading and maths, and are assessed in maths, science, and English reading and writing by teachers.

Key Stage 2: covers Year 3 (age 7 to 8) to Year 6 (age 10 to 11). At the end of Key Stage 2, children take national tests in English reading, maths, and grammar, punctuation and spelling, and are assessed by teachers in English writing and science.

Key Stage 3: covers Years 7 (Age 11 to 12) to 9 (age 13 to 14). Year 7 is considered the start of "secondary" school. After 2008, the national curriculum does not require any assessments. Before, 2008 there were Standardised Assessment Tests (SATs).

Key Stage 4: covers educational Years 10 (age 14 to 15) and 11 (age 15 to 16). For most students, this includes being examined using the (International) General Certificate of Secondary Education (GCSE). Special schools provide NCFE qualifications which are highly reputable vocational and work-related courses, designed to accommodate the needs of employers for immediate full-time employment and allow students to progress to higher education to degree level.

For peer review only - http://bmjopen.bmj.com/site/about/guidelines.xhtml

Appendix 3 - Additional Results & Methods

Appendix Table 1 – Overview of number of children entering primary school (Year 1) captured in ECHILD

Academic year	All children in school in Year 1 (recorded in NPD)	All children in school in Year 1 linked to a HES record	All children in school in Year 1 linked to a birth admission in HES
2009/10	574,833	543,135 (94.5%)	449,166 (78.1%)
2010/11	587,163	553,705 (94.3%)	474,295 (80.8%)
2011/12	600,455	565,764 (94.2%)	489,041 (81.4%)
2012/13	620,754	584,215 (94.1%)	498,376 (80.3%)
2013/14	647,299	605,217 (93.5%)	504,161 (77.9%)
2014/15	645,292	601,087 (93.1%)	527,385 (81.7%)
2015/16	659,361	613,953 (93.1%)	556,171 (84.3%)
2016/17	669,197	623,097 (93.1%)	570,284 (85.2%)
total	5,004,354	4,690,173 (93.7%)	4,068,879 (81.3%)

HES=Hospital Episode Statistics, NPD=National Pupil Database

Search terms for literature review for health phenotype definition

("cognitive impairment*" OR "cognitive delay*" OR "learning disabilit*" OR "intellectual disabilit*" OR "*developmental disability* " OR "developmental delay*" OR "special education" OR "special educational needs" OR "special educational need" OR "additional learning need*" OR "special school*" OR "additional learning support") AND ("systematic review" OR "meta-analysis" OR "meta analysis" OR "cohort study" OR "observational study" OR "population based" OR "population-based" OR "register study" OR "registry") AND (child* or adolescen* or pupil* or teeange* or "school student*") AND (congenital or "birth defect*" or chromosomal or "chronic disease" or "chronic condition*" or "long term condition*" or "long term disease" or "life limiting" or "end stage" or palliative or "liver disease" or asthma or cancer or malignan* or diabetes or obes* or encephalpath* or "cystic fibrosis" or "renal disease" or "kidney disease" or "heart disease" or cardiomyopathy or endocarditis or "lung disease" or "liver disease" or epilepsy or epileptic* or seizure* or "sickle cell" or "physical condition" or or illness or "brain injury" or "brain trauma" or cardiovascular or stroke or preterm or "birth weight" or birthweight or neonatal or gestation* or asphyxia or "spinal injury" or hydrocephalus or "nervous system" or autoimmune or eczema or arthritis or psoriasis or infection or medication)

For peer review only

For peer review only - http://bmjopen.bmj.com/site/about/guidelines.xhtml

Appendix Table 2 – Roadmap for causal investigations in HOPE, with an exemplar of cleft lip and palate

	**Steps in the design of the study:**	**Exemplar: *Children with cleft lip and palate (CLP) abnormalities***
**1.**	Articulate the scientific question and specify the background knowledge:	*Does special educational needs (SEN) provision improve the health and educational outcomes of children with CLP?*
	a) define the population of interest	Children with CLP identified in HES before age 5, born between 2003 and 2012, who started compulsory education between 2008 and 2018, with linked HES and NPD data
	b) specify the outcomes	• Number of days in contact with an accident and emergency department by Year 6<br>• Number of unplanned school absences by Year 6
	c) specify the intervention ("exposure")	• SEN support vs no recorded SEN provision<br>• SEN support vs EHCP
	d) draw assumptions regarding exposure, outcome and their common causes	*Draw DAG that includes unmeasured variables (as relevant) and identifies a minimum set of confounding variables*
	e) translate the causal question in terms of a contrast of means of potential outcomes*	*What are the benefits for the children who did receive SEN provision?*<br>→ Average treatment effect in the treated (ATT)<br><br>*Would other children with CLP benefit from SEN?*<br>→ Average treatment effect in the non-treated (ATNT)<br><br>*What would be the consequence of a new policy that increases the provision of SEN for all children with CLP? Or for those with more severe CLP?*<br>→ Interventional treatment effect (ITE)
**2.**	Can the question be addressed with the data at hand?	
	a) is the exposure well-defined and available in the data?	We have access to two categories of recorded SEN provision (SEN support and EHCP) and treat each of them to represent delivered support (covering a variety of different interventions)
	b) is the exposure suitable/available for everyone in the population of interest?	Some groups of children appear not to be eligible for an EHCP
	c) is there an issue of selection bias?	Missing values affect some of the confounding variables
**3.**	Causal contrasts	

For peer review only - http://bmjopen.bmj.com/site/about/guidelines.xhtml

	a) For which (sub-)population we wish to address the causal question?	Depending on the question:<br>• Children with CLP who have a record of any SEN provision;<br>• Children with CLP who do not have a record of any SEN provision;<br>• All children with CLP;<br>• Children with more severe CLP;
	b) On which scale?	For both outcomes we will examine:<br>– rate ratios<br>– rate differences
**4.**	Estimation:	
	a) Which estimation approach would target the causal contrast we are interested in?	For the ATT and ATNT:<br>• G-computation;<br>• Inverse probability weighting (IPW) of marginal structural models (MSMs), with alternative approaches to specify the propensity score;<br>• Doubly robust methods;<br>• Difference-in-Differences;<br><br>For the ITE:<br>• G-computation<br>• IPW of MSMs, with alternative approaches to specify the propensity score<br>• Doubly robust methods
	b) Are the assumptions invoked by alternative estimation approaches defensible?	• Unmeasured confounding may be at play;<br>• Parametric models used may be misspecified but robust methods can be used;
**5.**	Interpretation	
	a) Are the results comparable? And why not if not?	If comparable, results are more robust to misspecification and unmeasured confounding
	b) Triangulating results and compare with external evidence	Current evidence on impact is limited

**Potential outcomes are the outcomes that would occur under intervention on the exposure ATNT= Average treatment effect in the non-treated, ATT=Average treatment effect in the treated, CLP= cleft lip and palate, DAG=Directed Acyclic Graph, EHCP=Education, Health and Care Plan, HES=Hospital Episode Statistics, IPW=Inverse Probability Weighting, ITE=Interventional treatment effect, MSM=Marginal Structural Models, NPD=National Pupil Database, SEN=Special Educational Needs*

For peer review only

For peer review only - http://bmjopen.bmj.com/site/about/guidelines.xhtml

References

1. Department for Education. Special educational needs and disability code of practice: 0 to 25 years. Published online 2015. doi:DFE-00205-2013

2. Jay MA, Grath-Lone LM, Gilbert R. Data Resource: the National Pupil Database (NPD). *International Journal of Population Data Science*. 2019;4(1). doi:10.23889/ijpds.v4i1.1101

3. The National Audit Office. Support for pupils with special educational needs and disabilities in England. Published online 2019. Accessed January 4, 2023. https://www.nao.org.uk/wp-content/uploads/2019/09/Support-for-pupils-with-special-education-needs.pdf

4. Jay MA, Gilbert R. Special educational needs, social care and health. *Archives of Disease in Childhood*. 2021;106(1):83-85. doi:10.1136/archdischild-2019-317985

5. Hutchinson, Jo. Identifying pupils with special educational needs and disabilities. Published online 2021. Accessed October 5, 2021. https://epi.org.uk/wp-content/uploads/2021/03/SEND-Indentification_2021-EPI.pdf

6. Department for Education. 01 - Pupils in all schools, by type of SEN provision - including independent schools and general hospital schools - 2016 to 2022. Accessed January 23, 2023. https://explore-education-statistics.service.gov.uk/

7. Department for Education. Special educational needs in England, Academic Year 2021/22. Published June 16, 2022. Accessed January 23, 2023. https://explore-education-statistics.service.gov.uk/find-statistics/special-educational-needs-in-england#dataBlock-678da1e3-97e6-42f1-165e-08da47b0392d-tables

8. Hernan MA, Robins JM. *Causal Inference: What If*. Boca Raton: Chapman & Hall/CRC.; 2020.

9. Cullen MA, Lindsay G, Hastings R, Denne L, Stanford C. *Special Educational Needs in Mainstream Schools: Evidence Review*. Education Endowment Foundation; 2020. Accessed January 4, 2023. https://eric.ed.gov/?id=ED612232

10. Department for Education. The national curriculum. Accessed July 4, 2023. https://www.gov.uk/national-curriculum

For peer review only

For peer review only - http://bmjopen.bmj.com/site/about/guidelines.xhtml
